# Genetic tool development in marine protists: emerging model organisms for experimental cell biology

**DOI:** 10.1038/s41592-020-0796-x

**Published:** 2020-04-06

**Authors:** Drahomíra Faktorová, R. Ellen R. Nisbet, José A. Fernández Robledo, Elena Casacuberta, Lisa Sudek, Andrew E. Allen, Manuel Ares, Cristina Aresté, Cecilia Balestreri, Adrian C. Barbrook, Patrick Beardslee, Sara Bender, David S. Booth, François-Yves Bouget, Chris Bowler, Susana A. Breglia, Colin Brownlee, Gertraud Burger, Heriberto Cerutti, Rachele Cesaroni, Miguel A. Chiurillo, Thomas Clemente, Duncan B. Coles, Jackie L. Collier, Elizabeth C. Cooney, Kathryn Coyne, Roberto Docampo, Christopher L. Dupont, Virginia Edgcomb, Elin Einarsson, Pía A. Elustondo, Fernan Federici, Veronica Freire-Beneitez, Nastasia J. Freyria, Kodai Fukuda, Paulo A. García, Peter R. Girguis, Fatma Gomaa, Sebastian G. Gornik, Jian Guo, Vladimír Hampl, Yutaka Hanawa, Esteban R. Haro-Contreras, Elisabeth Hehenberger, Andrea Highfield, Yoshihisa Hirakawa, Amanda Hopes, Christopher J. Howe, Ian Hu, Jorge Ibañez, Nicholas A. T. Irwin, Yuu Ishii, Natalia Ewa Janowicz, Adam C. Jones, Ambar Kachale, Konomi Fujimura-Kamada, Binnypreet Kaur, Jonathan Z. Kaye, Eleanna Kazana, Patrick J. Keeling, Nicole King, Lawrence A. Klobutcher, Noelia Lander, Imen Lassadi, Zhuhong Li, Senjie Lin, Jean-Claude Lozano, Fulei Luan, Shinichiro Maruyama, Tamara Matute, Cristina Miceli, Jun Minagawa, Mark Moosburner, Sebastián R. Najle, Deepak Nanjappa, Isabel C. Nimmo, Luke Noble, Anna M. G. Novák Vanclová, Mariusz Nowacki, Isaac Nuñez, Arnab Pain, Angela Piersanti, Sandra Pucciarelli, Jan Pyrih, Joshua S. Rest, Mariana Rius, Deborah Robertson, Albane Ruaud, Iñaki Ruiz-Trillo, Monika A. Sigg, Pamela A. Silver, Claudio H. Slamovits, G. Jason Smith, Brittany N. Sprecher, Rowena Stern, Estienne C. Swart, Anastasios D. Tsaousis, Lev Tsypin, Aaron Turkewitz, Jernej Turnšek, Matus Valach, Valérie Vergé, Peter von Dassow, Tobias von der Haar, Ross F. Waller, Lu Wang, Xiaoxue Wen, Glen Wheeler, April Woods, Huan Zhang, Thomas Mock, Alexandra Z. Worden, Julius Lukeš

**Affiliations:** 1 Institute of Parasitology, Biology Centre, Czech Academy of Sciences and Faculty of Sciences, University of South Bohemia, České Budějovice, Czech Republic; 2 Department of Biochemistry, University of Cambridge, Cambridge, UK; 3 Bigelow Laboratory for Ocean Sciences, East Boothbay, ME USA; 4 Institut de Biologia Evolutiva, CSIC-Universitat Pompeu Fabra, Barcelona, Spain; 5 Monterey Bay Aquarium Research Institute, Moss Landing, CA USA; 6 Integrative Oceanography Division, Scripps Institution of Oceanography, University of California, San Diego, CA USA; 7 Microbial and Environmental Genomics, J. Craig Venter Institute, La Jolla, CA USA; 8 Molecular, Cell and Developmental Biology, University of California, Santa Cruz, CA USA; 9 The Marine Biological Association, Plymouth and School of Ocean and Earth Sciences, University of Southampton, Southampton, UK; 10 School of Biological Sciences, University of Nebraska, Lincoln, NE USA; 11 Gordon and Betty Moore Foundation, Palo Alto, CA USA; 12 Department of Molecular and Cell Biology, University of California, Berkeley, CA USA; 13 Sorbonne Université, CNRS UMR7621, Observatoire Océanologique, Banyuls sur Mer, France; 14 Institut de Biologie de l’Ecole Normale Supérieure (IBENS), Ecole Normale Supérieure, CNRS, INSERM, Université PSL, Paris, France; 15 Centre for Comparative Genomics and Evolutionary Bioinformatics, Dalhousie University, Halifax, Nova Scotia Canada; 16 Department of Biochemistry and Robert-Cedergren Centre for Bioinformatics and Genomics, Université de Montréal, Montreal, Quebec Canada; 17 Institute of Cell Biology, University of Bern, Bern, Switzerland; 18 Center for Tropical and Emerging Global Diseases, University of Georgia, Athens, GA USA; 19 School of Marine and Atmospheric Sciences, Stony Brook University, Stony Brook, NY USA; 20 Department of Botany, University of British Columbia, Vancouver, British Columbia Canada; 21 University of Delaware College of Earth, Ocean and Environment, Lewes, DE USA; 22 Woods Hole Oceanographic Institution, Woods Hole, MA USA; 23 Facultad Ciencias Biológicas, Pontificia Universidad Católica de Chile, Fondo de Desarrollo de Areas Prioritarias, Center for Genome Regulation and Millennium Institute for Integrative Biology (iBio), Santiago de Chile, Chile; 24 School of Biosciences, University of Kent, Canterbury, Kent, UK; 25 Laboratory of Molecular and Evolutionary Parasitology, University of Kent, Kent, UK; 26 Graduate School of Life and Environmental Sciences, University of Tsukuba, Ibaraki, Japan; 27 Department of Mechanical Engineering, Massachusetts Institute of Technology, Boston, MA USA; 28 Department of Organismic and Evolutionary Biology, Harvard University, Cambridge, MA USA; 29 Centre for Organismal Studies, University of Heidelberg, Heidelberg, Germany; 30 Department of Parasitology, Faculty of Science, Charles University, BIOCEV, Vestec, Czech Republic; 31 Faculty of Life and Environmental Sciences, University of Tsukuba, Ibaraki, Japan; 32 School of Environmental Sciences, University of East Anglia, Norwich, UK; 33 Graduate School of Life Sciences, Tohoku University, Sendai, Miyagi Japan; 34 Division of Environmental Photobiology, National Institute for Basic Biology, Okazaki, Aichi Japan; 35 Department of Marine Sciences, University of Connecticut , Groton, CT USA; 36 School of Biosciences and Veterinary Medicine, University of Camerino, Camerino, Italy; 37 Department of Basic Biology, School of Life Science, Graduate University for Advanced Studies, Okazaki, Aichi Japan; 38 Instituto de Biología Molecular y Celular, CONICET, and Facultad de Ciencias Bioquímicas y Farmacéuticas, Universidad Nacional de Rosario, Rosario, Argentina; 39 Center for Genomics and Systems Biology, New York University, New York, NY USA; 40 Biological and Environmental Science and Engineering Division, King Abdullah University of Science and Technology, Thuwal, Saudi Arabia; 41 Center for Zoonosis Control, Global Institution for Collaborative Research and Education, Hokkaido University, Sapporo, Japan; 42 Department of Ecology and Evolution, Stony Brook University, Stony Brook, NY USA; 43 Lasry Center for Biosciences, Clark University, Worcester, MA USA; 44 Departament de Genètica Microbiologia i Estadıśtica, Universitat de Barcelona, Barcelona, Spain; 45 Catalan Institution for Research and Advanced Studies, Barcelona, Spain; 46 Department of Systems Biology, Harvard Medical School, Boston, MA USA; 47 Wyss Institute for Biologically Inspired Engineering, Harvard University, Boston, MA USA; 48 Department of Environmental Biotechnology, Moss Landing Marine Laboratories, Moss Landing, CA USA; 49 Department of Molecular Genetics and Cell Biology, University of Chicago, Chicago, IL USA; 50 Department of Biology, California Institute of Technology, Pasadena, CA USA; 51 Instituto Milenio de Oceanografia de Chile, Concepción, Chile; 52 Institute of Oceanography, Minjiang University, Fuzhou, China; 53 Ocean EcoSystems Biology Unit, Marine Ecology Division, Helmholtz Centre for Ocean Research, Kiel, Germany; 54 Present Address: School of Biosciences, University of Nottingham, Sutton Bonington, UK; 55 Present Address: AGADA Biosciences Inc., Halifax, Nova Scotia Canada; 56 Present Address: Institute de Biologie de l’ENS, Département de biologie, École Normale Supérieure, CNRS, INSERM, Paris, France; 57 Present Address: Max Planck Institute for Developmental Biology, Tübingen, Germany

**Keywords:** Genetic models, Molecular biology

## Abstract

Diverse microbial ecosystems underpin life in the sea. Among these microbes are many unicellular eukaryotes that span the diversity of the eukaryotic tree of life. However, genetic tractability has been limited to a few species, which do not represent eukaryotic diversity or environmentally relevant taxa. Here, we report on the development of genetic tools in a range of protists primarily from marine environments. We present evidence for foreign DNA delivery and expression in 13 species never before transformed and for advancement of tools for eight other species, as well as potential reasons for why transformation of yet another 17 species tested was not achieved. Our resource in genetic manipulation will provide insights into the ancestral eukaryotic lifeforms, general eukaryote cell biology, protein diversification and the evolution of cellular pathways.

## Abstract (web summary)

This Resource describes genetic tools for microbial eukaryotes, providing a roadmap for developing genetically tractable organisms.

## Main

The ocean represents the largest continuous planetary ecosystem, hosting an enormous variety of organisms, which include microscopic biota such as unicellular eukaryotes (protists). Despite their small size, protists play key roles in marine biogeochemical cycles and harbor tremendous evolutionary diversity^1,2^. Notwithstanding their significance for understanding the evolution of life on Earth and their role in marine food webs, as well as driving biogeochemical cycles to maintain habitability, little is known about their cell biology including reproduction, metabolism and signaling^3^. Most of the biological knowledge available is based on comparison of proteins from cultured species to homologs in genetically tractable model taxa^4–7^. A main impediment to understanding the cell biology of these diverse eukaryotes is that protocols for genetic modification are only available for a small number of species^8,9^ that represent neither the most ecologically relevant protists nor the breadth of eukaryotic diversity.

The development of genetic tools requires reliable information about gene organization and regulation of the emergent model species. Over the last decade, genome^4–6^ and transcriptome sequencing initiatives^7^ have resulted in nearly 120 million unigenes being identified in protists^10^, which facilitates the developments of genetic tools used for model species. Insights from these studies enabled the phylogenetically informed approach^7^ for selecting and developing key marine protists into model systems in the Environmental Model Systems (EMS) Project presented herein. Forty-one research groups took part in the EMS Project, a collaborative effort resulting in the development of genetic tools that significantly expand the number of eukaryotic lineages that can be manipulated, and that encompass multiple ecologically important marine protists.

Here, we summarize detailed methodological achievements and analyze results to provide a synthetic ‘transformation roadmap’ for creating new microeukaryotic model systems. Although the organisms reported here are diverse, the paths to overcome difficulties share similarities, highlighting the importance of building a well-connected community to overcome technical challenges and accelerate the development of genetic tools. The 13 emerging model species presented herein, and the collective set of genetic tools from the overall collaborative project, will not only extend our knowledge of marine cell biology, evolution and functional biodiversity, but also serve as platforms to advance protistan biotechnology.

## Results

### Overview of taxa in the EMS initiative

**Fig. 1:**
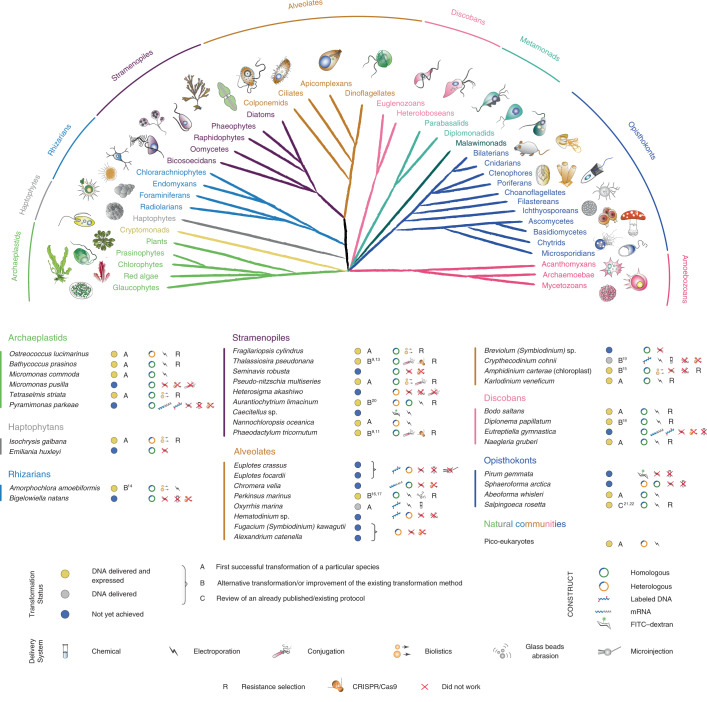
Phylogenetic relationships and transformation status of marine protists. A schematic view of the eukaryotic tree of life with effigies of main representatives. Color-coordinated species we have attempted to genetically modify are listed below. Current transformability status is schematized in circles indicating: DNA delivered and shown to be expressed (yellow, for details see text and Table 1); DNA delivered, but no expression seen (gray) and no successful transformation achieved despite efforts (blue). The details of transformation of species that belong to ‘DNA delivered’ and ‘Not achieved yet’ categories are described in Supplementary Table 5. mRNA, messenger RNA; FITC–dextran, fluorescein isothiocyanate (FITC)-conjugated dextran.

Taxa were selected from multiple eukaryotic supergroups^1,7^ to maximize the potential of cellular biology and to evaluate the numerous unigenes with unknown functions found in marine protists (Fig. 1). Before the EMS initiative, reproducible transformation of marine protists was limited to only a few species such as *Thalassiosira pseudonana*, *Phaeodactylum tricornutum* and *Ostreococcus tauri* (Supplementary Table 1). The EMS initiative included 39 species, specifically, 6 archaeplastids, 2 haptophytes, 2 rhizarians, 9 stramenopiles, 12 alveolates, 4 discobans and 4 opisthokonts (Fig. 1). Most of these taxa were isolated from coastal habitats, the focus area of several culture collections^7^. More than 50% of the selected species are considered photoautotrophs, with another 35% divided between heterotrophic osmotrophs and phagotrophs, the remainder being predatory mixotrophs. Almost 20% of the chosen species are symbionts and/or parasites of marine plants or animals, 5% are associated with detritus and several are responsible for harmful algal blooms (Supplementary Table 2).

**Fig. 2:**
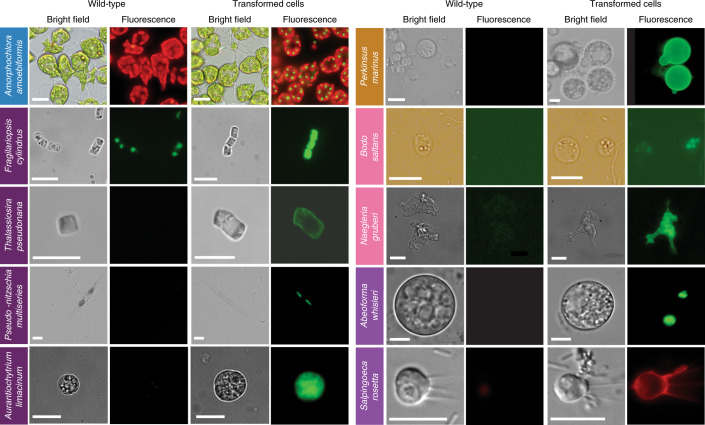
Epifluorescence micrographs of transformed marine protists. Representative images of transformants and wild-type cell lines of ten selected protist species. Colored boxes behind species names refer to phylogenetic supergroup assignments given in Fig. 1. Representative data of at least two independent experiments are shown. The fluorescent images show the expression of individual fluorescent marker genes introduced via transformation for all organisms shown, except in the case of *A. amoebiformis*. For this, red depicts the natural autofluorescence of photosynthetic pigments in the cell, while the additional green spheres in the transformant fluorescence panel shows introduced GFP fluorescence (see Supplementary Fig. 15c for a trace of these different regions in the cell). Scale bars are as follows: 10 µm for *A. amoebiformis*, *T. pseudonana*, *A. limacinum*, *B. saltans*, *N. gruberi*, *A. whisleri* and *S. rosetta*; 15 µm for *P. marinus*; 20 µm for *F. cylindrus* and 100 µm for *P. multiseries*.

**Fig. 3:**
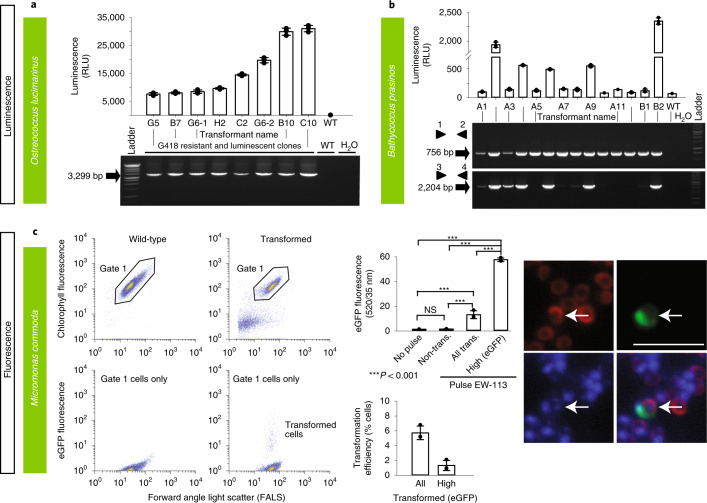
Various methods were used to demonstrate successful transformation in different archaeplastid species: luminescence and fluorescence. **a**–**c**, Luminescence (**a**,**b**) and fluorescence (by FACS and epifluorescence microscopy) (**c**) were used to verify expression of introduced constructs in three archaeplastids: *O. lucimarinus* (**a**), *B. prasinos* (**b**) and *M. commoda* (**c**). For the latter, red in the image depicts the natural autofluorescence of photosynthetic pigments in the cell, while green shows introduced eGFP fluorescence and blue shows the DAPI-stained nucleus; the overlay shows colocalization of eGFP and nucleus signals. See Supplementary Fig. 15d for a trace of these different regions in the cell. NS, not significant; trans., transformed. Representative data of at least two independent experiments are shown. For a detailed figure description see Supplementary Notes 2. Source data

**Fig. 4:**
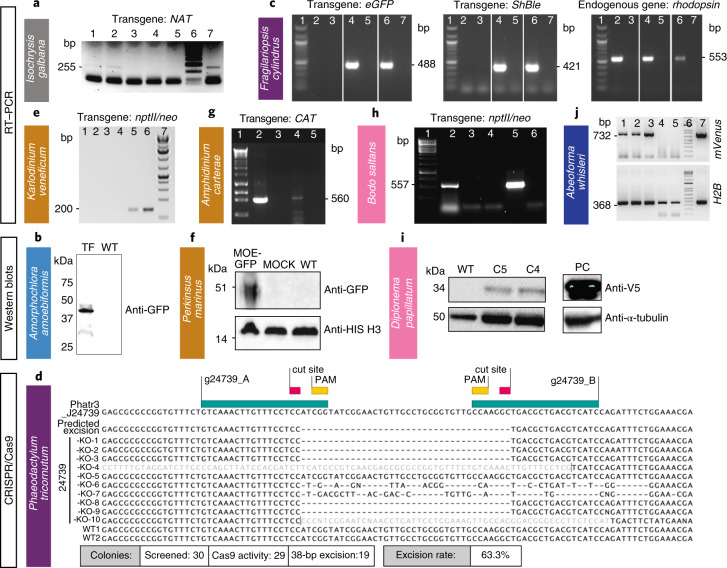
Various methods were used to demonstrate successful transformation in different species: RT–PCR, western blot and sequencing. **a**–**j**, Western blot, RT–PCR or sequencing (in case of Cas9-induced excision by CRISPR) were used to verify expression of introduced constructs in one haptophyte: *I. galbana* (**a**), one rhizarian—*A. amoebiformis* (**b**), two stramenopiles—*F. cylindrus* (**c**) and *P. tricornutum* (**d**), three alveolates—*K. veneficum* (**e**), *P. marinus* (**f**) and *A. carterae* (**g**), two discobans—*B. saltans* (**h**) and *D. papillatum* (**i**) and one opisthokont—*A. whisleri* (**j**). Note that *nptII/neo* is used synonymously with amino 3′-glycosyl phosphotransferase gene (*aph*(3′)) conferring resistance to kanamycin and neomycin. Representative data of at least two independent experiments are shown. For a detailed figure description see Supplementary Notes 2. Source data

**Fig. 5:**
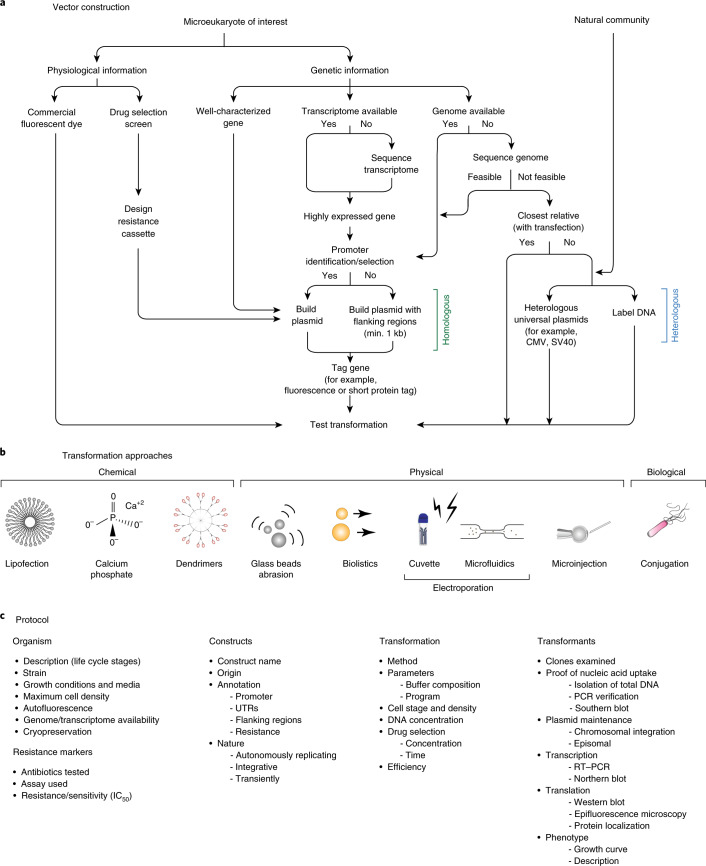
‘Transformation roadmap’ for the creation of genetically tractable protists. **a**, Vector design and construction for microeukaryotes of interest and a natural community. **b**, Transformation approaches. Different symbols represent methods (for example chemical, physical or biological) for introducing DNA/RNA/protein into a living cell. **c**, Protocol. Key methodological steps for successful transformation are listed in an abbreviated form (for particular examples, see Table 1 and text).

While some transformation systems for protists have been developed in the past^8,9,11^, the challenge for this initiative was to develop genetic tools for species that not only require different cultivation conditions but are also phenotypically diverse. It should be noted that not all main lineages were explored. For example, amoebozoans did not feature in this aquatic-focused initiative, in part because they tend to be most important in soils, at least based on current knowledge, and manipulation systems exist for members of this eukaryotic supergroup, such as *Dictyostelium discoideum*^12^. The overall EMS initiative outcomes are summarized in Fig. 1 and Table 1. We provide detailed protocols for 13 taxa, for which no transformation systems have been previously reported (category A) and eight taxa, for which existing protocols^9,11,13–21^ were advanced (category B; Figs. 2, 3 and 4, Table 1, Supplementary Tables 1–5 and Methods). We also review an already published EMS transformation protocol^22^ in one species (category C), and we discuss unsuccessful transformation attempts for 17 additional taxa (Fig. 1 and Methods). Finally, we synthesize our findings in a roadmap for the development of transformation systems in protists (Fig. 5).

**Table 1:** Parameters used for successful transformation as shown in Figs. 2, 3 and 4

Species	Transformation method/device	Cell number (input)	Vector amount (µg)	Promotor	Regulatory elements	Drug selection (µg ml^−1^)	Time selection (d)	Efficiency (%)	Transformation status (stable, S; transient, T)	Reporter	Evidence of transformation	protocols.io link
**Archaeoplastids**												
*Ostreococcus lucimarinus* (RCC802)	Electroporation Genepulser II	1–2 × 10^9^	Plasmid PotLuc; linear; 5	HAPT, Histone H4 *O. tauri*	None	G418 (1,000)	10–21	<0.0001	S	Luc	G418 resist, luminescence, PCR	https://www.protocols.io/view/selection-of-stable-transformants-in-ostreococcus-zj2f4qe; https://www.protocols.io/view/transient-luciferase-expression-in-ostreococcus-ot-hcib2ue; https://www.protocols.io/view/transient-transformation-of-ostreococcus-species-o-g86bzze
*Bathycoccus prasinos* (RCC4222)	Electroporation Genepulser II	1–2 × 10^9^	Fusion PCR; *p*HAPT: pLucpH4:KanM; linear; 5	HAPT, Histone H4 Endogenous	None	G418 (1,000)	10–21	<0.0001	S	Luc	G418 resist, luminescence PCR	10.17504/protocols.io.g86bzze; 10.17504/protocols.io.zj2f4qe; 10.17504/protocols.io.hcib2ue
*Micromonas commoda* (CCMP2709)	Electroporation Lonza-Nucleofector	3 × 10^7^	RPS9pro^Mco^-eGFP-NLS-RPS9ter in pUC05-AMP; circular; 10–20	Endogenous, ribosomal protein S9;	Endogenous, ribosomal protein S9;	NA	2–6	5.6 ± 1.3 (of post-transformation population)	T^a^	eGFP	Per cell eGFP fluorescence, fluorescence microscopy	http://doi.org/10.17504/protocols.io.8p9hvr6
	Electroporation Lonza-Nucleofector	3 × 10^7^	H3pro^Mpo^-LUC-H3ter in pUC05-AMP; circular; 10–20	Histone H3 5′ UTR from *M. polaris*	Histone H3 3′ end formation -histone stem loop from *M. polaris*	NA	3	NA (Luc. assay is bulk, not per cell)	T^a^	NanoLuc	Luminescence	http://doi.org/10.17504/protocols.io.8p8hvrw
*Tetraselmis striata* (KAS-836)	Bio-Rad Biolistics PDS-1000/He biolistics system	2.0 × 10^7^	pACTpro:Ble; linear; 1.0	Actin, *T. striata*	Actin, *T. striata*	Zeocin (150)	21–28		S		Zeocin resist, PCR	http://doi.org/10.17504/protocols.io.hjtb4nn
**Haptophytes**												
*Isochrysis galbana* (CCMP1323)	Biolistics PDS-1000/He	1–2 × 10^6^	pIgNAT; circular; 1.0	Hsp70 *E. huxleyi*	Heterologous	Nourseothricin (80–150)	14	<0.0001	S	None	Nourseothricin resistance, PCR, RT–PCR	https://www.protocols.io/view/biolistic-transformation-of-isochrysis-galbana-2pugdnw; https://www.protocols.io/view/method-for-electroporation-of-isochrysis-galbana-c-hmab42e
**Rhizarians**												
*Amorphochlora (Lotharella) amoebiformis* (CCMP2058)	Electroporation Gene Pulser Xcell	0.5–1 × 10^7^	GFP-Rubisco; circular; 30–50	rbcS1, Endogenous	rbcS1 Endogenous	Manual selection of fluorescent cells^a^	NA	NA	S/T	GFP	Fluorescence, western blot	http://doi.org/10.17504/protocols.io.35hgq36
**Stramenopiles**												
*Fragilariopsis cylindrus* (CCMP1102)	Bio-Rad Biolistics PDS-1000/He biolistics system	5 × 10^7^	pUC:FCP:ShBle:FCP:eGFP; circular; 1.0	FCP, Endogenous	None	Zeocin (100)	21–49	0.00003 (30 c.f.u. per 10^8^ cells)	S	eGFP	Zeocin resist, fluorescence, PCR, RT–PCR	10.17504/protocols.io.z39f8r6
*Thalassiosira pseudonana* (CCMP1335)	Bacterial conjugation	4 × 10^7^	TpSIl3p-eGFP in pTpPuc3; circular; NA	Endogenous	Endogenous	Nourseothricin (100 in plates, 200 in liquid culture)	~14	~10	T	eGFP	Nourseothricin resistance, colony PCR, fluorescence	http://doi.org/10.17504/protocols.io.nbzdap6; http://doi.org/10.17504/protocols.io.7ghhjt6
*Pseudo-nitzschia multiseries*	Conjugation	1 × 10^5^	Pm_actP_egfp_actT; pPtPUC3	Pm actin; Pt fcpB	None, other than contained in promoter/term	Manual selection of fluorescent cells in LGTA; zeocin (200)	24 h, 7	<0.1%	T	eGFP, shble	Fluorescence, vector targeted PCR on gDNA	http://doi.org/10.17504/protocols.io.4pzgvp6
*Aurantiochytrium limacinum* (ATCC MYA-1381)	Bio-Rad Gene Pulser (165-2076) NEPA21	1 × 10^8^	18GZG 18GeZG plasmid; linear; 1–10	Endogenous GAPDH	None	Zeocin (100)	5–7	44 per μg of DNA	S	eGFP, shble	Zeocin resist., PCR, Southern, fluorescence	http://doi.org/10.17504/protocols.io.h3nb8me
*Nannochloropsis oceanica* (CCMP1779)	Electroporation Genepulser II	1 × 10^9^	pMOD, linear/circular; 0.1–1	CMV	None	None	0.1–1	20 (linear), 1–2 (circular)	T	mTagBFP2	Fluorescence, PCR, RT–PCR	http://doi.org/10.17504/protocols.io.h3nb8me
*Phaeodactylum tricornutum* (CCAP1055/1)	Bacterial conjugation	4 × 10^7^	hCas9-2A-shble PtpBR episome 100 µl *E. coli* OD_600_ = 0.9	FcpF-hCas9 psRNA–sgRNA	Cen6-Arsh4-His3 centromere	Phleomycin (50) Zeocin (100)	10–16	1.25 × 10^−5^ ≈ 500 c.f.u.	S	shble (Cas9) yfp VENUS	Phleoycin resistance, PCR maintained episome, PCR Cas9 target site	http://doi.org/10.17504/protocols.io.4bmgsk6; http://doi.org/10.17504/protocols.io.7gihjue; http://doi.org/10.17504/protocols.io.7gnhjve
**Alveolates**												
*Perkinsus marinus* (ATCC PRA240)	Electroporation LONZA-Nucleofector Glass beads abrasion (425–600 μm)	5–7 × 10^7^	pPmMOE-GFP; linear-circular (1:1); 5	Endogenous	Endogenous	FACS Blasticidin (50–200), puromycin (10-50), bleo (50-200)	Drug: 20–60 FACS: 3	0.01–5	S	GFP, mCherry	Fluorescence sequencing, PCR, western blot	https://www.protocols.io/view/oyster-parasite-perkinsus-marinus-transformation-u-gv9bw96; https://www.protocols.io/view/glass-beads-based-transformation-protocol-for-perk-g36byre; https://www.protocols.io/view/fluorescence-activated-cell-sorting-facs-of-perkin-hh2b38e
*Oxyrrhis marina* (CCMP 1788, CCMP 1795)	Electroporation Gene Pulser Xcell; Chemical (CaCl_2_)	1–5 × 10^6^; 1 × 10^5^	Fluorescently labeled DNA (5–25 µg) or FITC–dextran; mCherry	NA, endogenous hsp90	NA, endogenous hsp90	NA	NA	0.5–5%	T	mCherry	Fluorescence	https://www.protocols.io/view/electroporation-of-oxyrrhis-marina-vcne2ve; https://www.protocols.io/view/transfection-of-alexa488-labelled-dna-into-oxyrrhi-ha8b2hw; https://www.protocols.io/view/electroporation-transformation-of-fitc-dextran-int-3cmgiu6; https://www.protocols.io/view/co-incubation-protocol-for-transforming-heterotrop-hmzb476
*Crypthecodinium cohnii* (CCMP 316)	Electroporation LONZA-Nucleofector		Stained DNA (739 bp); linear; 1	None	None	NA	NA	<0.001	T	Fluorescence	Fluorescence	https://www.protocols.io/view/transfection-of-crypthecodinium-cohnii-using-label-z26f8he
*Amphidinium carterae* (chloroplast) (CCMP1314)	Bio-Rad Biolistics PDS-1000/He biolistics system	2.5 × 10^7^	pAmpAtpBChl; circular; 0.5	Endogenous	Endogenous	Chloramphenicol (20)	3 onward	NA	S	Ab res	RT–PCR, PCR phenotype	http://doi.org/10.17504/protocols.io.4r2gv8e
*Karlodinium veneficum* (CCMP1975)	Electroporation	4 × 10^5^	linear-DinoIII-neo; linear; 2	Endogenous	Endogenous	Kanamycin (150)	7	0.0005	S (3 mon)	NA	RT–PCR, PCR	https://www.protocols.io/view/nucleofector-protocol-for-dinoflagellates-using-lo-qm8du9w
**Discobans (euglenozoans and heteroloboseans)**												
*Bodo saltans* (submitted to ATCC)	Electroporation Nepa21	1–1.5 × 10^7^	Bs-EF1- α C-terminal tagging; linear; 3–5	Endogenous	Endogenous	G418 (5)	7–9		S	GFP	Fluorescence, PCR, RT–PCR	http://doi.org/10.17504/protocols.io.s5jeg4n; http://doi.org/10.17504/protocols.io.7fchjiw
*Diplonema papillatum* (ATCC 50162)	Electroporation Lonza-Nucleofector	5 × 10^7^	p57-V5+Neo^R^; linear; 3	Endogenous	Endogenous	G418 (75)	7–14	~5.5	S	NA	Western blot (resistance marker), RT–PCR	http://doi.org/10.17504/protocols.io.4digs4e
*Naegleria gruberi* (ATCC 30224)	Electroporation Bio-Rad Gene Pulser xCell	5 × 10^6^	pNAEG-HYG; circular; 4	Endogenous	Endogenous	Hygromycin (300) Neo (700)	15–28	80	T	GFP	Western blot (resistance marker), fluorescence,	http://doi.org/10.17504/protocols.io.hpub5nw; http://doi.org/10.17504/protocols.io.7w4hpgw
**Opisthokonts**												
*Abeoforma whisleri* (ATCC PRA-279)	Electroporation Lonza-Nucleofector	3 × 10^5^	Awhis_H2Bvenus+ pUC19; circular; 1–5 + 40 carrier	Endogenous	Endogenous	NA	10–15	1	T	Venus	Fluorescence, RT–PCR	http://doi.org/10.17504/protocols.io.zexf3fn
*Salpingoeca rosetta* (ATCC PRA-390)	Electroporation Lonza-Nucleofector	4 × 10^5^	SrActmCherry-CCTLL +pUC19; circular; 1–10 + 40 carrier	Endogenous	Endogenous	Puromycin (80)	10–12		S	mCherry	Gene expression (Luc, fluorescence)/resistance	http://doi.org/10.17504/protocols.io.h68b9hw

For additional information, see protocols.io links, Supplementary Table 5 and Supplementary Note 1. For contacting laboratories working with particular species, see details given in Supplementary Table 6.

^a^May be stable but overgrown by wild-type strain.

NA, not applicable; NLS, nuclear localization signal.

#### Archaeplastids

Prasinophytes are important marine green algae distributed from polar to tropical regions^23^. They form a sister group to chlorophyte algae, and together, these two groups branch adjacent to land plants, collectively comprising the Viridiplantae, which are part of the Archaeplastida^1,23^ (Fig. 1). Genome sequences are available for the picoprasinophytes (<3 µm cell diameter) tested herein, specifically, *Micromonas commoda*, *M. pusilla*, *Ostreococcus lucimarinus* and *Bathycoccus prasinos*. As part of the EMS initiative, we report on genetic tools for *Bathycoccus*, a scaled, nonmotile genus, and *Micromonas*, a motile, naked genus with larger genomes than *Bathycoccus* and *Ostreococcus*^22^. We also report on genetic tools for *Tetraselmis striata* and *O. lucimarinus*. The latter was transformed based on an adapted homologous recombination system for *O. tauri*^24,25^.

*O. lucimarinus* (RCC802) and *B. prasinos* (RCC4222) were transformed using protocols adapted from *O. tauri*^24,25^. Briefly, using electroporation for transfer of exogenous genes, *O. lucimarinus* was transformed using a DNA fragment encoding the *O. tauri* high-affinity phosphate transporter (*HAPT*) gene fused to a luciferase gene and a kanamycin selection marker (Table 1 and Supplementary Table 3), which resulted in transient luciferase expression 24 h after electroporation (Table 1 and Fig. 3a). After 2 weeks of growth in low-melting agarose plates containing G418 (1 mg ml^−1^), 480 colonies were obtained, picked and grown in artificial seawater with the antibiotic neomycin. Of these, 76 displayed luminescence ≥2.5-fold above background (80 relative luminescence units (RLU)), with widely variable levels (200–31,020 RLU), likely reflecting either variations in the site of integration and/or the number of integrated genes (Fig. 3a, Supplementary Fig. 1 and Methods).

The *O. tauri* construct did not work in *B. prasinos*, while the use of the *B. prasinos* histone *H4* and *HAPT* sequences in an otherwise identical construct and conditions was successful. Although luciferase expression was not detected 24 h after electroporation, 48 G418-resistant colonies were obtained 2 weeks later, 20 being luminescent when grown in liquid medium. Analysis of 14 resistant transformants revealed that the luciferase sequence was integrated into the genome of five luminescent clones, and one nonluminescent clone (Fig. 3b and Methods), suggesting that the chromatin context at integration sites in the latter was not favorable to luciferase expression.

Although transformation methods successful for *Bathycoccus* and *Ostreococcus* failed in *Micromonas*, Lonza nucleofection was successful with *M. commoda* (CCMP2709) (Table 1 and Fig. 3c) using two different codon-optimized plasmids, one encoding the luciferase gene (NanoLuc, Promega) flanked by an exogenous promoter and terminator sequence from the 5′ and 3′ untranslated regions (UTRs) of histone *H3* in *Micromonas polaris* (CCMP2099), and the other encoding an enhanced green fluorescent protein (*eGFP*) gene flanked by endogenous promoter and terminator sequences from ribosomal protein S9 (Supplementary Table 5). Sensitivities to antibiotics were established (Supplementary Table 3). Constructs did not include a selectable marker, as we aimed to introduce and express foreign DNA while developing conditions suitable for transfection that supported robust growth in this cell wall-lacking protist (Table 1). Transformants revealed a significantly higher level of eGFP fluorescence than wild-type cells, with 1.3% of the population showing fluorescence per cell 45-fold higher than both the nontransformed portion of the culture and the wild-type cells (Fig. 3c and Methods). Additionally, the RLU was 1,500-fold higher than controls when using the luciferase-bearing construct, such that multiple experiments with both plasmids confirmed expression of exogenous genes in *M. commoda*.

*T. striata* (KAS-836) was transformed using microprojectile bombardment (Supplementary Fig. 2a). Two selectable marker genes were tested, consisting of a putative promoter and 5′ UTR sequences from the *T. striata* actin gene and either the coding sequences of the *Streptoalloteichus hindustanus* bleomycin gene (conferring resistance to zeocin) or the *Streptomyces hygroscopicus bar* gene (conferring resistance to glufosinate) (Table 1, Supplementary Fig. 2a and Methods). The terminator sequence was obtained from the *T. striata* glyceraldehyde-3-phosphate dehydrogenase gene. Linearized plasmids were coated on gold particles and introduced into *T. striata* cells by using the PDS-1000/He Particle Delivery System (Bio-Rad). Transformants were successfully selected on half-strength f/2 at 50% salinity agar plates containing either 150 μg ml^−1^ zeocin or 150 μg ml^−1^ glufosinate.

#### *Haptophytes* (incertae sedis)

Haptophytes are a group of photosynthetic protists that are abundant in marine environments and include the principal calcifying lineage, the coccolithophores. Genome sequences are available for *Emiliania huxleyi*^6^ and *Chrysochromulina tobin*^26^, and there is one report of nuclear transformation of a calcifying coccolithophore species^27^ but transformation of *E. huxleyi*, the most prominent coccolithophore, has not been achieved yet^27^. Here, as part of the EMS initiative, a stable nuclear transformation system was developed for *Isochrysis galbana*, a species that lacks coccoliths, but represents an important feedstock for shellfish aquaculture^28^.

*I. galbana* (CCMP1323) was transformed by biolistic bombardment with the pIgNAT vector, which contains nourseothricin (NTC) *N*-acetyltransferase (*NAT*), (for nourseothricin resistance) driven by the promoter and terminator of *Hsp70* from *E. huxleyi* (CCMP1516). Twenty-four hours after bombardment, cells were transferred to liquid f/2 medium at 50% salinity containing 80 µg ml^−1^ NTC and left to grow for 2–3 weeks to select for transformants (Table 1). The presence of *NAT* in NTC-resistant cells was verified by PCR and PCR with reverse transcription (RT–PCR) (Fig. 4a, Supplementary Fig. 2b and Methods) and the sequence was verified. To confirm NTC resistance was a stable phenotype, cells were subcultured every 2–4 weeks at progressively higher NTC concentrations (up to 150 µg ml^−1^) in the above-mentioned media. Cells remained resistant to NTC for approximately 6 months, as confirmed by PCR screening to identify the presence of the *NAT* gene.

#### Rhizarians

Rhizarians include diverse nonphotosynthetic protists, as well as the photosynthetic chlorarachniophytes that acquired a plastid via secondary endosymbiosis of a green alga^4^. Uniquely, they represent an intermediate stage of the endosymbiotic process, since their plastids still harbor a relict nucleus (nucleomorph). Here, we report on an advanced transformation protocol for the chlorarachniophyte *Amorphochlora (Lotharella) amoebiformis* for which low-efficiency transient transformation has previously been achieved using particle bombardment^14^.

*A. amoebiformis* (CCMP2058) cells were resuspended in 100 µl of Gene Pulse Electroporation Buffer (Bio-Rad) with 20–50 µg of the reporter plasmid encoding eGFP-RubisCO fusion protein under the control of the native *rbcS1* promoter and subjected to electroporation (Table 1). Cells were immediately transferred to fresh ESM medium and incubated for 24 h. Transformation efficiency was estimated by the fraction of cells expressing eGFP, resulting in 0.03–0.1% efficiency, as enumerated by microscopy, showing an efficiency up to 1,000-fold higher than in the previous study^14^ (Table 1). Stable transformants were generated by manual isolation using a micropipette, and a transformed line has maintained eGFP fluorescence for at least 10 months without antibiotic selection (Figs. 2 and 4b and Methods).

#### Stramenopiles

Stramenopiles are a diverse lineage harboring important photoautotrophic, mixotrophic (combining photosynthetic and phagotrophic nutrition) and heterotrophic taxa. As the most studied class in this lineage, diatoms (Bacillariophyceae) were early targets for the development of reverse genetics tool^11,29^. Diatoms are estimated to contribute approximately 20% of annual carbon fixation^30^ and, like several other algal lineages, are used in bioengineering applications and biofuels^31^. Although other cold-adapted eukaryotes have, to our knowledge, yet to be transformed, here we present a protocol for the Antarctic diatom *Fragilariopsis cylindrus*^32^. A transformation protocol has also been developed herein for *Pseudo-nitzschia multiseries*, a toxin-producing diatom^33^. Here we also present work for nondiatom stramenopiles, including a transformation protocol for the eustigmatophyte *Nannochloropsis oceanica*, and an alternative protocol for the labyrinthulomycete *Aurantiochytrium limacinum*^20^, both of which are used for biotechnological applications. Furthermore, we report on advances for CRISPR/Cas-driven gene knockouts in *Phaeodactylum tricornutum*^8,13^ and a more efficient bacterial conjugation system for *Thalassiosira pseudonana*^13^.

Microparticle bombardment was used on *F. cylindrus* (CCMP1102) that was grown, processed and maintained at 4 °C in 24 h light. Exponential phase cells were harvested onto a 1.2 µm membrane filter that was then placed on an 1.5% agar Aquil plate for bombardment with beads coated with a plasmid containing zeocin resistance and *eGFP*, both controlled by an endogenous fucoxanthin chlorophyll *a/c* binding protein (FCP) promoter and terminator (Table 1, Supplementary Table 3 and Methods)^34^. Transformation was performed using 0.7 µm tungsten particles and the biolistic particle delivery system PDS-1000/He (Bio-Rad). Rupture disks for 1,350 and 1,550 pounds per square inch (psi) gave the highest colony numbers with efficiencies of 20.7 colony forming units (c.f.u.) per 10^8^ cells and 30 c.f.u. per 10^8^ cells, respectively. Following bombardment, the filter was turned upside down and left to recover for 24 h on the plate, then cells were rinsed from the plate/filter and spread across five 0.8% agar Aquil plates with 100 µg ml^−1^ zeocin. Colonies appeared 3–5 weeks later. PCR on genomic DNA showed that 100 and 60% of colonies screened positive for the bleomycin gene (*ShBle*) for zeocin resistance and the gene encoding eGFP, respectively. As confirmed by fluorescence-activated cell sorting (FACS) and microscopy, eGFP was localized to the cytosol and was distinguishable from plastid autofluorescence (Fig. 2). Additional confirmation by PCR and RT–PCR (Fig. 4c) revealed that the *ShBle* and *eGFP* genes were present in the genomes of transformants after multiple transfers (>10) 2 years later, indicating long-term stability.

Bacterial conjugation methods were improved in *T. pseudonana* (CCMP1335) using the silaffin precursor *TpSil3p* (Table 1 and Methods) as the target gene. *TpSil3p* was fused to *eGFP* flanked by an FCP promoter and terminator, cloned into a pTpPuc3 episomal backbone and transformed into mobilization plasmid-containing EPI300 *E. coli* cells (Lucigen). The donor cells were grown in super optimal broth with catabolite repression (SOC) medium at 37 °C until OD_600_ of 0.3–0.4, centrifuged and resuspended in 267 μl SOC medium. Next, 200 μl of donor cells were mixed with *T. pseudonana* cells, cocultured on predried 1% agar plates, dark incubated at 30 °C for 90 min, then at 18 °C in constant light for 4 h, followed by selection in 0.25% agar plates containing 100 µg ml^−1^ NTC. Colonies were observed after 2 weeks, inoculated into 300 μl L1 medium and supplemented with 200 µg ml^−1^ NTC to reduce the number of false positives. Positive transformants were identified by colony PCR screening (Supplementary Fig. 3) and epifluorescence microscopy (Fig. 2).

The diatom *P. multiseries* (15093C) and other members of this genus form buoyant linear chains with overlapping cell tips during active growth, and were unconducive to punctate colony formation on agar, where their growth is generally poor. To address this challenge, a low-gelation-temperature agarose seawater medium (LGTA) was developed to facilitate growth, antibiotic selection and cell recovery. *P. multiseries* exhibited growth inhibition at relatively low concentrations under NTC, formaldehyde and zeocin (Supplementary Table 3). Biolistic transformation of two other *P.* species had been demonstrated at low efficiency^35^. To complement this approach and explore potentially higher efficiency methods for transformation with diatom episomal plasmids, we modified the existing conjugation-based method^13^. The published conjugation protocol was modified to enhance *P. multiseries* postconjugation viability by reducing SOC content. An episomal version of the Pm_actP_egfp_actT expression cassette was transfected into *E. coli* EPI300+pTAMOB and used for conjugation (Table 1 and Methods). After 48 h in L1 medium, cells were plated in LGTA and eGFP-positive cells were observed 7 d later (Fig. 2). PCR revealed the presence of plasmids in all eGFP-positive colonies (Supplementary Fig. 4). Similarly, conjugation with the episome pPtPUC3 (bleomycin selection marker)-containing bacterial donors was followed under zeocin selection (200 μg ml^−1^). After 7 d, only viable cells (based on bright chlorophyll fluorescence) contained the episome, as confirmed by PCR. Propagation of transformants after the first medium transfer (under selection) has so far been unsuccessful.

Stable transformation of *A. limacinum* (ATCC MYA-1381) was achieved by knock-in of a resistance cassette composed of *ShBle* driven by 1.3 kb promoter and 1.0 kb terminator regions of the endogenous glyceraldehyde-3-phosphate dehydrogenase gene carried in a pUC19-based plasmid (18GZG) along with the native 18S ribosomal RNA gene, and by knock-in of a similar construct containing a *eGFP:ShBle* fusion (Supplementary Fig. 5). Approximately 1 × 10^8^ cells were electroporated, adapting the electroporation protocol used for *Schizochytrium*^36^. The highest transformation efficiency was achieved using 1 µg of linearized 18GZG plasmid with two pulses, resulting in a time constant of ~5 ms (Table 1 and Methods). Expression of the fusion protein was confirmed by both the zeocin-resistance phenotype and the detection of eGFP (Fig. 2). Six 18GZG transformants derived from uncut and linearized plasmids were examined in detail. All maintained antibiotic resistance throughout 13 serial transfers, first in selective, then subsequently in nonselective media and then again in selective medium. Integration of the plasmid into the genome was confirmed by PCR as well as by Southern blots using a digoxigenin-labeled *ShBle* gene probe, showing that four transformants had integrations by single homologous recombination, while in two transformants additional copies of the antibiotic resistance cassette were integrated by nonhomologous recombination elsewhere in the genome (Supplementary Fig. 5).

Electroporation of *N. oceanica* (CCMP1779) was optimized based on observation of cells treated with fluorescein-conjugated 2,000 kDa dextran and subsequent survival (Table 1 and Methods). A sorbitol concentration of 800 mM and electroporation at between 5 and 9 kV cm^−1^ resulted in highest cell recovery. These conditions were used during introduction of plasmids containing the gene for the blue fluorescent reporter mTagBFP2 under the control of cytomegalovirus (*CMV*), the cauliflower mosaic virus *35S*, or the *VCP1* promoter previously described from *Nannochloropsis* sp.^37^. Transient expression of blue fluorescence (compared to cells electroporated simultaneously under the same conditions without plasmid) appeared within 2 h, lasted for at least 24 h and disappeared by 48 h in subsets of cells electroporated with *mTagBFP2* under the control of *CMV* (Supplementary Fig. 6). The transient transformation was more effective when a linearized plasmid was used compared to a circular plasmid (Table 1). *VCP1* did not induce blue fluorescence with a circular plasmid, while *35S* gave inconsistent results with either circularized or linearized plasmids.

For *P. tricornutum* (CCAP1055/1), we adapted the CRISPR/Cas9 system^8^ for multiplexed targeted mutagenesis. Bacterial conjugation^13^ was used to deliver an episome that contained a Cas9 cassette and two single-guide RNA (sgRNA) expression cassettes designed to excise a 38 basepair-long domain from the coding region of a nuclear-encoded, chloroplastic glutamate synthase (*Phatr3_J24739*) and introduce an in-frame stop codon after strand ligation (Table 1 and Methods). The GoldenGate assembly was used to clone two expression cassettes carrying sgRNAs into a *P. tricornutum* episome that contained a *Cas9–2A-ShBle* expression cassette and the centromeric region CenArsHis (Supplementary Fig. 7). After their addition to a *P. tricornutum* culture, plates were incubated in a growth chamber under standard growth conditions for 2 d and transformed *P. tricornutum* colonies began to appear after 2 weeks. Only colonies maintaining *Cas9–2A-ShBle* sequence on the delivered episome were able to grow on selection plates because *Cas9* and *ShBle* were transcriptionally fused by the 2A peptide^38^ (Supplementary Fig. 7). Gel electrophoresis migration and sequencing of the genomic target loci confirmed the 38 bp-long excision and premature stop codon (Fig. 4d).

#### Alveolates

This species-rich and diverse group comprises ciliates, apicomplexans and dinoflagellates (Fig. 1). As a link between apicomplexan parasites and dinoflagellate algae, perkinsids are key for understanding the evolution of parasitism, and also have potential biomedical applications^17^. Techniques currently exist for transformation of only a small number of ciliates, perkinsids and apicomplexans^39^. Here, we present a transformation protocol for *Karlodinium veneficum* (CCMP1975), a phagotrophic mixotroph that produces fish-killing karlotoxins^40^. Experiments were also performed on *Oxyrrhis marina* (CCMP 1788/CCMP 1795), a basal-branching phagotroph that lacks photosynthetic plastids and *Crypthecodinium cohnii* (CCMP 316), a heterotroph used in food supplements. For both of these taxa, evidence of DNA delivery was achieved (Table 1, Supplementary Results, Supplementary Fig. 15 and Methods), a goal recently achieved for *C. cohnii* using electroporation^19^. Additionally, we report on improved transformation systems for *Perkinsus marinus* (PRA240) and *Amphidinium carterae* (CCMP1314) chloroplast, published recently as part of the EMS initiative^15^.

*K. veneficum* (CCMP1975) was transformed based on electroporation and cloning the selectable marker gene aminoglycoside 3′-phosphotransferase (*nptII/neo*; note that *nptII/neo* is used synonymously with amino 3′-glycosyl phosphotransferase gene conferring resistance to kanamycin, neomycin, paromomycin, ribostamycin, butirosin and gentamicin B) into the backbone of the dinoflagellate-specific expression vector DinoIII-neo^41^, which confers resistance to neomycin and kanamycin (Table 1). In brief, DinoIII-neo was linearized and electroporated using the Nucleofector optimization pulse codes, buffer SF/Solution I (Lonza), and 2 μg μl^−1^ of linearized DinoIII-neo. Electroporated cells were selected under 150 μg ml^−1^ kanamycin 3 d postelectroporation. Fresh seawater with kanamycin was added every 2 weeks to the cultures and new subcultures were inoculated monthly. After 3 months, DNA and RNA were isolated from the resistant cultures as previously reported^42^ and cDNA was synthesized using random hexamers. Out of 16 transformations, two cell lines (CA-137, DS-138) showed stable growth under kanamycin selection. CA-137 developed dense cultures after 3 months, and the resistance gene was detected in both DNA and RNA by nested PCR and RT–PCR, respectively (Fig. 4e, Supplementary Fig. 8 and Methods).

We improved the transformation protocol^16,17^ of *P. marinus*, a pathogen of marine mollusks, fish and amphibians^43^ (Supplementary Table 5). We coexpressed two genes and efficiently selected transient and stable transformants using FACS (Table 1, Figs. 2 and 4f, Supplementary Fig. 9 and Methods). In addition, we established the integration profile of ectopic DNA once introduced into the *P. marinus* genome. We did not see evidence of integration through homologous recombination and observed a propensity for plasmid fragmentation and integration within transposable elements sites. An optimized alternative protocol for transformation using glass bead abrasion was also developed. Two versions of the previously published *Moe* gene promoter^16^ were tested. Whereas the 1.0 kb promoter version induced expression after 2 or 3 d, the truncated version (0.5 kb) took 7 d for expression to be detected. Resistance genes to zeocin, blasticidin and puromycin have all been shown to confer resistance to transformed *P. marinus*; however, selection regimes are still relatively slow and inefficient, indicating further room for improvement^17^.

We also report a vector for the transformation of the *A. carterae* chloroplast, a photosynthetic dinoflagellate. *A. carterae*, like other dinoflagellates with a peridinin-containing chloroplast, contains a fragmented chloroplast genome made up of multiple plasmid-like minicircles^40^. The previous transformation protocols made use of this to introduce two vectors based on the psbA minicircle^15^. Here, we show that other minicircles are also suitable for use as vectors. We created an artificial minicircle, using the atpB minicircle as a backbone, but replacing the *atpB* gene with a codon-optimized chloramphenicol acetyltransferase (Table 1 and Methods). This circular vector was introduced by biolistics to *A. carterae* (Supplementary Fig. 10a). Following selection with chloramphenicol, we were able to detect transcription of the chloramphenicol acetyltransferase gene via RT–PCR (Fig. 4g). This result suggests that all 20 or so minicircles in the dinoflagellate chloroplast genome would be suitable for use as artificial minicircles, thus providing a large pool of potential vectors.

#### Discobans

This diverse group, recently split into Discoba and Metamonada^44^, includes heterotrophs, photoautotrophs and predatory mixotrophs, as well as parasites. Discobans include parasitic kinetoplastids with clinical significance, such as *Trypanosoma brucei*, *T. cruzi* and *Leishmania* spp., for which efficient transformation protocols are available^45^. However, such protocols are missing for aquatic species. Here, we describe available transformation protocols for the kinetoplastid *Bodo saltans* and the heterolobosean *Naegleria gruberi*. The former was isolated from a lake, but identical 18S rRNA gene sequences have been reported from marine environments^46^. The latter is a freshwater protist that represents a model organism for closely related marine heterolobosean amoebas. Furthermore, we provide advanced methods that build on previous EMS results^18^ for the diplonemid *Diplonema papillatum*.

*B. saltans* (ATCC 30904) was transformed with a plasmid containing a cassette designed to fuse an endogenous *EF1-α* gene with *eGFP* for C-terminal tagging. This cassette includes downstream of *eGFP*, a *B. saltans* tubulin intergenic region followed by the selectable marker *nptII/neo* gene, conferring resistance to neomycin. *EF1-α* genes exist in tandem repeats. The homologous regions that flank the cassette were chosen as targets for inducing homology-directed repair; however, they target only one copy of the gene. As transcription in *B. saltans* is polycistronic^46^, insertion of the tubulin intergenic region into the plasmid is essential for polyadenylation of the *EF1-α/GFP* fusion and *trans*-splicing of the *nptII/neo* gene (Supplementary Table 5). Selection of transfected cells began with 2 µg ml^−1^ of neomycin added 24 h after electroporation, and this concentration was gradually increased over 2 weeks to 5 µg ml^−1^ (Table 1 and Methods). Cells were washed and subcultured into fresh selection medium every 4 d, and neomycin-resistant cells emerged 7–9 d postelectroporation. The eGFP signal was detected 2 d postelectroporation, albeit with low intensity. This may be due to the inefficient translation of *eGFP* since it has not been codon-optimized for *B. saltans* (Fig. 2). Genotyping analysis 9 months posttransfection confirmed the presence of the *nptII/neo* gene and at least partial plasmid sequence (Fig. 4h and Supplementary Fig. 10b). However, plasmid integration into the *B. saltans* genome through homologous recombination is still unconfirmed. This suggests either off-target plasmid integration or episomal maintenance.

For *N. gruberi* (ATCC 30224) two plasmids were designed. The first one carried the hygromycin B resistance gene (*hph*) with an actin promoter and terminator, along with an HA-tagged *eGFP* driven by the ubiquitin promoter and terminator. The second plasmid carried the *nptII/neo* gene instead. For each individual circular plasmid, 4 μg was electroporated (Table 1 and Methods). About 48 h after electroporation, dead cells were removed from the suspension and viable cells were washed with PBS. Afterward, 300 μg ml^−1^ of hygromycin B or 700 μg ml^−1^ of neomycin was added to the fresh media. One to 4 weeks later, resistant clones were recovered and expression of eGFP and/or hygromycin was confirmed by western blotting (Supplementary Fig. 11). Expression of eGFP was observed by epifluorescence microscopy (Fig. 2 and Supplementary Fig. 11) with ~80% of transformants maintaining hygromycin B or neomycin resistance in addition to expressing eGFP.

*D. papillatum* (ATCC 50162) was transformed by electroporation using 3 μg of a *SwaI*-linearized fragment (cut from p57-V5+NeoR plasmid) containing the V5-tagged *nptII/neo* gene flanked by partial regulatory sequences derived from the hexokinase gene of the kinetoplastid *Blastocrithidia* (strain p57) (Table 1 and Methods) using a published protocol^18^. About 18 h after electroporation, 75 μg ml^−1^ G418 was added to the medium and after 2 weeks, seven neomycin-resistant clones were recovered. Transcription of *nptII/neo* was verified in four clones by RT–PCR (Supplementary Fig. 12) and the expression of the tagged nptII/neo protein was confirmed in two clones by western blotting using the α-V5 antibody (Fig. 4i).

#### Opisthokonts

The opisthokont clade Holozoa includes animals and their closest unicellular relatives choanoflagellates, filastereans, ichthyosporeans and corallochytreans. The establishment of genetic tools in nonmetazoan holozoans promises to help illuminate the cellular and genetic foundations of animal multicellularity^47^. Genomic and transcriptomic data are available for multiple representatives characterized by diverse cell morphologies, some of which can even form multicellular structures^46^. Until recently, only transient transformations had been achieved for some opistokonts such as the filasterean *Capsaspora owczarzaki*^48^, the ichthyosporean *Creolimax fragrantissima*^49^ and the choanoflagellate *Salpingoeca rosetta*^21^. Through the EMS initiative, we report on evidence for transient transformation of the ichthyosporean *Abeoforma whisleri*, isolated from the digestive tract of mussels, and review a recently published stable transformation protocol for *S. rosetta* achieved by using the selectable puromycin *N*-acetyl-transferase gene (Fig. 2)^22^.

All *A. whisleri* life stages are highly sensitive to a variety of methods for transformation. However, we developed a 4D-nucleofection-based protocol using 16-well strips, wherein PBS-washed cells were resuspended in 20 μl of buffer P3 (Lonza) containing 40 μg of carrier plasmid (empty pUC19) and 1–5 μg of the reporter plasmid (*A. whisleri* *H2B* fused to mVenus fluorescent protein, *mVFP*) (Table 1 and Methods), and subjected to code EN-138 (Lonza). Immediately after the pulse, cells were recovered by adding 80 μl of marine broth (Gibco) before plating in 12-well culture plates previously filled with 1 ml marine broth. After 24 h, ~1% of the culture was transformed based on the fraction of cells expressing mVFP in the nucleus (Figs. 2 and 4j).

### Microbial eukaryotes in natural planktonic communities

Model organisms are typically selected based on criteria such as relative ease of isolation and asexual cultivation in the laboratory; however, these attributes may not correlate with the capacity for uptake and expression of the exogenous DNA. We explored whether natural marine planktonic pico- and nanoeukaryote communities would take up DNA in a culture-independent setting. Microbial plankton from natural seawater was concentrated and electroporated with plasmids containing *mTagBFP2* under the control of CMV or 35S promoters (Supplementary Results and Methods). In most trials, blue fluorescent cells were rare if detected at all (compared to control samples). However, in one natural community tested, a photosynthetic picoeukaryote population exhibited up to 50% of cells with transient expression of blue fluorescence when the CMV promoter was used (Supplementary Fig. 13). This suggests it might be possible to selectively culture eukaryotic microorganisms based on capacity to express exogenous DNA.

## Discussion

The collaborative effort by the EMS initiative facilitated identification and optimization of the steps required to create new protist model systems, which culminated in the synthetic transformation roadmap (Fig. 5). Our genetic manipulation systems for aquatic (largely marine) protists will enable deeper insights into their cell biology, with potentially valuable outcomes for aquatic sciences, evolutionary studies, nanotechnology, biotechnology, medicine and pharmacology. Successes and failures with selectable markers, transformation conditions and reporters were qualitatively compared across species (Supplementary Tables 3 and 4, Table 1, Figs. 2–4 and  Methods).

For some of the selected species, the first step was to identify cultivation conditions for robust growth in the laboratory to either generate high cell densities or large culture volumes for obtaining sufficient biomass required for a variety of molecular biology experiments. Unlike established microbial model species, cultivation of marine protists can be challenging, especially under axenic conditions or for predatory taxa that require cocultivation with their prey. Nevertheless, 13 out of 35 species were rendered axenic before the development of transformation protocols. For the remaining species, we were unable to remove bacteria and therefore had to make sure that transformation signals were coming from the targeted protist rather than contaminants (Supplementary Table 2). Subsequent steps included the identification of suitable antibiotics and their corresponding selectable markers (Table 1 and Supplementary Table 3), conditions for introducing exogenous DNA (Table 1 and Supplementary Table 4) and selection of promoter and terminator sequences for designing transformation vectors (Table 1, Methods, Supplementary Table 5 and Supplementary Notes 1).

As exemplified in the model systems provided herein (Table 1 and Figs. 2–4), a variety of methods were used to test whether exogenous DNA was integrated into the genome or maintained as a plasmid, and whether the introduced genes were expressed. Approaches to show the former included inverse PCR, Southern blotting and whole genome sequencing, whereas approaches to demonstrate the latter included various combinations of PCR, RT–PCR, western blotting, epifluorescence microscopy, FACS, antibody-based methods and/or growth assays in the presence of antibiotics to confirm transcription and translation of introduced selection and reporter genes (for example, *eGFP*, *YFP*, *mCherry*). For fluorescent markers, it was first ensured that the wild-type, or manipulated controls cells, had no signals conflicting with the marker (Figs. 2 and 3c), an important step because photosynthetic protists contain chlorophyll and other autofluorescent pigments. Overall transformation outcomes for each species were parsed into three groups according to the level of success or lack thereof (A, first transformation protocol for a given species; B, advanced protocol based on previous work and C, published protocol based on the EMS initiative) and are discussed according to their phylogenetic position (Fig. 1).

Our studies did not result in a universally applicable protocol because transformability and a range of other key conditions varied greatly across taxa and approaches, such as intrinsic features of the genome and differences in cellular structure and morphology. In general, electroporation proved to be the most common method for introducing exogenous DNA stably into cells. This approach was used for naked cells and protoplasts, yet frequently also worked, albeit with lower efficiency, on cells protected by cell walls. Linearized plasmids were most effective for delivery, and 5′ and 3′ UTR-containing promotors of highly expressed endogenous genes provided the strongest expression of selective reporters and markers. If successful, teams usually continued with fluorescence-based methods. Furthermore, large amounts of carrier DNA usually facilitated successful initial transformations (for example, *M. commoda*, *A. whisleri*) or improved existing protocols (*S. rosetta*^21^). We also provide the contact details of all coauthors who are assigned to particular species (Supplementary Table 6).

Some lineages were difficult to transform, especially dinoflagellates and coccolithophores. Here, even if DNA appeared to be delivered (Supplementary Table 5), expression of the transformed genes could not be confirmed. Examples include the dinoflagellates *C. cohnii*, *Symbiodinium microadriaticum* and the coccolithophore *E. huxleyi*. Thus, at least these three species need concerted future efforts.

The combination of results presented herein together with previously published protocols from the EMS initiative^50^ significantly expands the segment of extant eukaryotic diversity amenable to reverse genetics approaches. Out of the 39 microbial eukaryotes selected for the initiative, exogenous DNA was delivered and expressed in more than 50% of them. The transformation systems enable us to shed light on the function of species-specific genes, which likely reflect key adaptations to specific niches in dynamic ocean habitats.

## Methods

### Studied species and used transformation methods

For the full list of vector sequences and maps see Supplementary Notes 1 and for detailed description of Figs. 3 and 4 see Supplementary Note 2. Antibiotic concentrations effective for selection of transformants can be found in Supplementary Table 3, the details of the transformation methods applied to this study in Supplementary Table 4 and contact details for individual laboratories in Supplementary Table 6. Full list of protists (including details of culture collection) and links to the complete step-by-step transformation protocols and published vector sequences are listed in Supplementary Table 5. The protocols.io links listed in Table 1 and Supplementary Table 5 are summarized in Supplementary Tables 7 and 8.

### Reporting Summary

Further information on research design is available in the Nature Research Reporting Summary linked to this article.

## Online content

Any methods, additional references, Nature Research reporting summaries, source data, extended data, supplementary information, acknowledgements, peer review information; details of author contributions and competing interests; and statements of data and code availability are available at 10.1038/s41592-020-0796-x.

## Supplementary information

Supplementary Information Supplementary Figs. 1–15, Results and Notes 1 and 2.

Reporting Summary

Supplementary Tables 1, 2, 4 and 5.

Supplementary Tables 3, 6, 7 and 8.

## Data Availability

The data that support the findings of this study are available from the corresponding authors as well as the other authors upon request (for the contacts see Supplementary Table 6). Source data for Figs. 3 and 4 and Supplementary Figs. 9b,c, 11a and 12b,c are available online.

## Source data

Source Data Fig. 3a–c Source Data Fig. 4a–c,e–j Source Data Supplementary Figures
