# Publisher Correction: Genetic tool development in marine protists: emerging model organisms for experimental cell biology

**DOI:** 10.1038/s41592-020-0828-6

**Published:** 2020-04-15

**Authors:** Drahomíra Faktorová, R. Ellen R. Nisbet, José A. Fernández Robledo, Elena Casacuberta, Lisa Sudek, Andrew E. Allen, Manuel Ares, Cristina Aresté, Cecilia Balestreri, Adrian C. Barbrook, Patrick Beardslee, Sara Bender, David S. Booth, François-Yves Bouget, Chris Bowler, Susana A. Breglia, Colin Brownlee, Gertraud Burger, Heriberto Cerutti, Rachele Cesaroni, Miguel A. Chiurillo, Thomas Clemente, Duncan B. Coles, Jackie L. Collier, Elizabeth C. Cooney, Kathryn Coyne, Roberto Docampo, Christopher L. Dupont, Virginia Edgcomb, Elin Einarsson, Pía A. Elustondo, Fernan Federici, Veronica Freire-Beneitez, Nastasia J. Freyria, Kodai Fukuda, Paulo A. García, Peter R. Girguis, Fatma Gomaa, Sebastian G. Gornik, Jian Guo, Vladimír Hampl, Yutaka Hanawa, Esteban R. Haro-Contreras, Elisabeth Hehenberger, Andrea Highfield, Yoshihisa Hirakawa, Amanda Hopes, Christopher J. Howe, Ian Hu, Jorge Ibañez, Nicholas A. T. Irwin, Yuu Ishii, Natalia Ewa Janowicz, Adam C. Jones, Ambar Kachale, Konomi Fujimura-Kamada, Binnypreet Kaur, Jonathan Z. Kaye, Eleanna Kazana, Patrick J. Keeling, Nicole King, Lawrence A. Klobutcher, Noelia Lander, Imen Lassadi, Zhuhong Li, Senjie Lin, Jean-Claude Lozano, Fulei Luan, Shinichiro Maruyama, Tamara Matute, Cristina Miceli, Jun Minagawa, Mark Moosburner, Sebastián R. Najle, Deepak Nanjappa, Isabel C. Nimmo, Luke Noble, Anna M. G. Novák Vanclová, Mariusz Nowacki, Isaac Nuñez, Arnab Pain, Angela Piersanti, Sandra Pucciarelli, Jan Pyrih, Joshua S. Rest, Mariana Rius, Deborah Robertson, Albane Ruaud, Iñaki Ruiz-Trillo, Monika A. Sigg, Pamela A. Silver, Claudio H. Slamovits, G. Jason Smith, Brittany N. Sprecher, Rowena Stern, Estienne C. Swart, Anastasios D. Tsaousis, Lev Tsypin, Aaron Turkewitz, Jernej Turnšek, Matus Valach, Valérie Vergé, Peter von Dassow, Tobias von der Haar, Ross F. Waller, Lu Wang, Xiaoxue Wen, Glen Wheeler, April Woods, Huan Zhang, Thomas Mock, Alexandra Z. Worden, Julius Lukeš

**Affiliations:** 1 Institute of Parasitology, Biology Centre, Czech Academy of Sciences and Faculty of Sciences, University of South Bohemia, České Budějovice, Czech Republic; 2 Department of Biochemistry, University of Cambridge, Cambridge, UK; 3 Bigelow Laboratory for Ocean Sciences, East Boothbay, ME USA; 4 Institut de Biologia Evolutiva, CSIC-Universitat Pompeu Fabra, Barcelona, Spain; 5 Monterey Bay Aquarium Research Institute, Moss Landing, CA USA; 6 Integrative Oceanography Division, Scripps Institution of Oceanography, University of California, San Diego, CA USA; 7 Microbial and Environmental Genomics, J. Craig Venter Institute, La Jolla, CA USA; 8 Molecular, Cell and Developmental Biology, University of California, Santa Cruz, CA USA; 9 The Marine Biological Association, Plymouth and School of Ocean and Earth Sciences, University of Southampton, Southampton, UK; 10 School of Biological Sciences, University of Nebraska, Lincoln, NE USA; 11 Gordon and Betty Moore Foundation, Palo Alto, CA USA; 12 Department of Molecular and Cell Biology, University of California, Berkeley, CA USA; 13 Sorbonne Université, CNRS UMR7621, Observatoire Océanologique, Banyuls sur Mer, France; 14 Institut de Biologie de l’Ecole Normale Supérieure (IBENS), Ecole Normale Supérieure, CNRS, INSERM, Université PSL, Paris, France; 15 Centre for Comparative Genomics and Evolutionary Bioinformatics, Dalhousie University, Halifax, Nova Scotia Canada; 16 Department of Biochemistry and Robert-Cedergren Centre for Bioinformatics and Genomics, Université de Montréal, Montreal, Quebec Canada; 17 Institute of Cell Biology, University of Bern, Bern, Switzerland; 18 Center for Tropical and Emerging Global Diseases, University of Georgia, Athens, GA USA; 19 School of Marine and Atmospheric Sciences, Stony Brook University, Stony Brook, NY USA; 20 Department of Botany, University of British Columbia, Vancouver, British Columbia Canada; 21 University of Delaware College of Earth, Ocean and Environment, Lewes, DE USA; 22 Woods Hole Oceanographic Institution, Woods Hole, MA USA; 23 Facultad Ciencias Biológicas, Pontificia Universidad Católica de Chile, Fondo de Desarrollo de Areas Prioritarias, Center for Genome Regulation and Millennium Institute for Integrative Biology (iBio), Santiago de Chile, Chile; 24 School of Biosciences, University of Kent, Canterbury, Kent, UK; 25 Laboratory of Molecular and Evolutionary Parasitology, University of Kent, Kent, UK; 26 Graduate School of Life and Environmental Sciences, University of Tsukuba, Ibaraki, Japan; 27 Department of Mechanical Engineering, Massachusetts Institute of Technology, Boston, MA USA; 28 Department of Organismic and Evolutionary Biology, Harvard University, Cambridge, MA USA; 29 Centre for Organismal Studies, University of Heidelberg, Heidelberg, Germany; 30 Department of Parasitology, Faculty of Science, Charles University, BIOCEV, Vestec, Czech Republic; 31 Faculty of Life and Environmental Sciences, University of Tsukuba, Ibaraki, Japan; 32 School of Environmental Sciences, University of East Anglia, Norwich, UK; 33 Graduate School of Life Sciences, Tohoku University, Sendai, Miyagi Japan; 34 Division of Environmental Photobiology, National Institute for Basic Biology, Okazaki, Aichi Japan; 35 Department of Marine Sciences, University of Connecticut, Groton, CT USA; 36 School of Biosciences and Veterinary Medicine, University of Camerino, Camerino, Italy; 37 Department of Basic Biology, School of Life Science, Graduate University for Advanced Studies, Okazaki, Aichi Japan; 38 Instituto de Biología Molecular y Celular, CONICET, and Facultad de Ciencias Bioquímicas y Farmacéuticas, Universidad Nacional de Rosario, Rosario, Argentina; 39 Center for Genomics and Systems Biology, New York University, New York, NY USA; 40 Biological and Environmental Science and Engineering Division, King Abdullah University of Science and Technology, Thuwal, Saudi Arabia; 41 Center for Zoonosis Control, Global Institution for Collaborative Research and Education, Hokkaido University, Sapporo, Japan; 42 Department of Ecology and Evolution, Stony Brook University, Stony Brook, NY USA; 43 Lasry Center for Biosciences, Clark University, Worcester, MA USA; 44 Departament de Genètica Microbiologia i Estadıśtica, Universitat de Barcelona, Barcelona, Spain; 45 Catalan Institution for Research and Advanced Studies, Barcelona, Spain; 46 Department of Systems Biology, Harvard Medical School, Boston, MA USA; 47 Wyss Institute for Biologically Inspired Engineering, Harvard University, Boston, MA USA; 48 Department of Environmental Biotechnology, Moss Landing Marine Laboratories, Moss Landing, CA USA; 49 Department of Molecular Genetics and Cell Biology, University of Chicago, Chicago, IL USA; 50 Department of Biology, California Institute of Technology, Pasadena, CA USA; 51 Instituto Milenio de Oceanografia de Chile, Concepción, Chile; 52 Institute of Oceanography, Minjiang University, Fuzhou, China; 53 Ocean EcoSystems Biology Unit, Marine Ecology Division, Helmholtz Centre for Ocean Research, Kiel, Germany; 54 Present Address: School of Biosciences, University of Nottingham, Sutton Bonington, UK; 55 Present Address: AGADA Biosciences Inc., Halifax, Nova Scotia Canada; 56 Present Address: Institute de Biologie de l’ENS, Département de biologie, École Normale Supérieure, CNRS, INSERM, Paris, France; 57 Present Address: Max Planck Institute for Developmental Biology, Tübingen, Germany

**Keywords:** Genetic models, Molecular biology

Correction to: *Nature Methods* 10.1038/s41592-020-0796-x, published online 6 April 2020.

This paper was originally published under standard Springer Nature copyright (© The Author(s), under exclusive licence to Springer Nature America, Inc.). It is now available as an open-access paper under a Creative Commons Attribution 4.0 International license. The error has been corrected in the print, PDF and HTML versions of the article.

