# Supplementary material for: Genetic tool development in marine protists: emerging model organisms for experimental cell biology
Source: Nat Methods. 2020 Apr 6;17(5):481–94. doi: 10.1038/s41592-020-0796-x (PMC7200600; doi:10.1038/s41592-020-0796-x)
Supplement: Supplementary file 2 — Reporting Summary [file 41592_2020_796_MOESM2_ESM.pdf]

nature research

Corresponding author(s): Drahomira Faktorova,Thomas Mock, Alexandra Worden, Julius Lukes,

Last updated by author(s): 09/01/2020

Reporting Summary

Nature Research wishes to improve the reproducibility of the work that we publish. This form provides structure for consistency and transparency in reporting. For further information on Nature Research policies, see Authors & Referees and the Editorial Policy Checklist.

Statistics

For all statistical analyses, confirm that the following items are present in the figure legend, table legend, main text, or Methods section.

n/a	Confirmed	
☐	☒	The exact sample size (*n*) for each experimental group/condition, given as a discrete number and unit of measurement
☐	☒	A statement on whether measurements were taken from distinct samples or whether the same sample was measured repeatedly
☐	☒	The statistical test(s) used AND whether they are one- or two-sided<br>*Only common tests should be described solely by name; describe more complex techniques in the Methods section.*
☒	☐	A description of all covariates tested
☒	☐	A description of any assumptions or corrections, such as tests of normality and adjustment for multiple comparisons
☐	☒	A full description of the statistical parameters including central tendency (e.g. means) or other basic estimates (e.g. regression coefficient) AND variation (e.g. standard deviation) or associated estimates of uncertainty (e.g. confidence intervals)
☐	☒	For null hypothesis testing, the test statistic (e.g. *F*, *t*, *r*) with confidence intervals, effect sizes, degrees of freedom and *P* value noted<br>*Give P values as exact values whenever suitable.*
☒	☐	For Bayesian analysis, information on the choice of priors and Markov chain Monte Carlo settings
☒	☐	For hierarchical and complex designs, identification of the appropriate level for tests and full reporting of outcomes
☒	☐	Estimates of effect sizes (e.g. Cohen's *d*, Pearson's *r*), indicating how they were calculated

Our web collection on statistics for biologists contains articles on many of the points above.

Software and code

Policy information about availability of computer code

Data collection	FACS softwares noted in our methods section are FloJo v.10.6.1, WinList 3D v7.0 (Verity Software House) and BD FACS Sortware v1.2.0.142.
Data analysis	FACS softwares noted in our methods section that were used for data analysis after collection on the FACS instrument are FloJo v.10.6.1 and WinList 3D v7.0 (Verity Software House).

For manuscripts utilizing custom algorithms or software that are central to the research but not yet described in published literature, software must be made available to editors/reviewers. We strongly encourage code deposition in a community repository (e.g. GitHub). See the Nature Research guidelines for submitting code & software for further information.

Data

Policy information about availability of data

All manuscripts must include a data availability statement. This statement should provide the following information, where applicable:

- Accession codes, unique identifiers, or web links for publicly available datasets
- A list of figures that have associated raw data
- A description of any restrictions on data availability

The data that support the findings of this study are available from the corresponding authors as well as the other authors upon request (for the contacts see Suppl. Table 5).

Field-specific reporting

Please select the one below that is the best fit for your research. If you are not sure, read the appropriate sections before making your selection.

☒ Life sciences ☐ Behavioural & social sciences ☐ Ecological, evolutionary & environmental sciences

For a reference copy of the document with all sections, see nature.com/documents/nr-reporting-summary-flat.pdf

Life sciences study design

All studies must disclose on these points even when the disclosure is negative.

Sample size	No sample size calculation was performed, rather standard practices in the field of genetics were used which involved replicating experiments the sample sizes and replication are the same or more than in most publications presenting developments in genetic techniques.
Data exclusions	No data was excluded from the analyses.
Replication	For each experiment, all attempts at replication were successful.
Randomization	The experiments were designed to determine whether a specific protocol rendered transformation in cells. Since clonal cell lines and the same starting material were used under pulse conditions/plasmids and negative controls and because these are not survey experiments randomization is not an appropriate aspect of the design
Blinding	Blinding was not appropriate to our study as all measurement were performed using the same clonal cell lines and analyzed with identical methodologies (within each experiment). Our experiments did not involve testing placebos nor were they what is termed randomized controlled trials (RCT) as appropriate in e.g. drug clinical trials.

Reporting for specific materials, systems and methods

We require information from authors about some types of materials, experimental systems and methods used in many studies. Here, indicate whether each material, system or method listed is relevant to your study. If you are not sure if a list item applies to your research, read the appropriate section before selecting a response.

Materials & experimental systems

n/a	Involved in the study	
☐	☒	Antibodies
☐	☒	Eukaryotic cell lines
☒	☐	Palaeontology
☒	☐	Animals and other organisms
☒	☐	Human research participants
☒	☐	Clinical data

Methods

n/a	Involved in the study	
☒	☐	ChIP-seq
☐	☒	Flow cytometry
☒	☐	MRI-based neuroimaging

Antibodies

Antibodies used

Commercial antibodies:
Anti-V5 tag Monoclonal Antibody (2F11F7)
Supplier: Invitrogen
Catalog number: 37-7500
LOT number: 1468908A
Dilution: 1:1,000

Monoclonal Anti-α-Tubulin
clone DM1A produced in mouse, ascites fluid
Supplier: Sigma Aldrich
Catalog Number: T9026
Lot #: N/A
Dilution: 1:2,000

Anti-GFP Living Colors® A.v. Monoclonal Antibody
Supplier: Takara
Cat. #: 632380
Clone name: JL-8
Lot #: N/A
Dilution: 1:1,000

Anti-GFP polyclonal antibody, Invitrogen
Cat.no. A11122
Clone name N/A
Lot no. 1828014
Dilution 1:1000

Anti-Histone H3 antibody, Abcam
Cat.no. ab1791
Clone name N/A (polyclonal)
Lot no. N/A
Dilution 1:1000

Anti-mCherry antibody, Abcam
Cat.no. ab167453
Clone name N/A (polyclonal)
Lot no. GR3213077-5
Dilution 1:1000

HYGROMYCIN PHOSPHOTRANSFERASE ANTIBODY (10-1421) (anti-hygromycin antibody, Fitzgerald Industries)
Host Mouse
Clone M1709Hy4
Isotype IgG1
Purity > 95% pure
Form & Buffer
Supplied in 50mM NaCl, 10 mM Gly-HCl, 0.05% NaN_3
Dilution: 1:2000

H9658- Monoclonal Anti-HA antibody produced in mouse (Sigma)
clone HA-7
Mouse IgG1 by RID
Protein by Biuret
WB-Cell Line/Tissue Extract
Dilution: 1:10,000

Anti-Mouse IgG (whole molecule)–Peroxidase antibody produced in rabbit
Supplier: Sigma Aldrich
Catalog Number: A9044
Lot #: N/A
Dilution: 1:2,000

Anti-Rabbit IgG (whole molecule)–Peroxidase antibody produced in goat
Supplier: Sigma Aldrich
Catalog Number: A6154
Lot #: N/A
Dilution: 1:2,000

Anti-Mouse IgG, HRP-Linked Whole Ab Sheep (secondary antibody)
Supplier: GE Healthcare
Cat. #: NA931-100UL
Clone name: N/A
Lot #: 380748
Dilution: 1:10,000

Goat Anti-rabbit HRP, ImmunoReagents Inc. (secondary antibody)
Cat. no. GtxRb-003-DHRPX
Clone name N/A
Lot no. N/A
Dilution 1:10000

Validation

Validation of anti-V5 tag Monoclonal Antibody (2F11F7): The manufacturer states that the antibody works on a wide range of species (human, bovine, amphibian and mouse cells or tissues as well as with yeast or fungi). https://www.thermofisher.com/antibody/product/V5-Tag-Antibody-clone-2F11F7-Monoclonal/37-7500
We tested it on D. papillatum and T. brucei.

Validation of Monoclonal Anti-α-Tubulin: Manufacturer´s validation: Monoclonal Anti-α-Tubulin is immunospecific for tubulin as determined by indirect immunofluorescent staining and immunoblotting procedures. The manufacturer states that the antibody works on a wide range of species (human, bovine, amphibian and mouse cells or tissues as well as with yeast or fungi). https://www.sigmaaldrich.com/catalog/product/sigma/t9026?lang=en&region=CZ
We tested it on D. papillatum and T. brucei.

Validation of Anti-GFP Living Colors® A.v. Monoclonal Antibody: Manufacturer's validation: The quality and performance of Living Colors A.v. Monoclonal Antibody (JL-8) was tested by Western blot analysis using lysate made from a HEK 293 cell line stably expressing AcGFP1.

Validation of Anti-GFP polyclonal antibody (Invitrogen): The manufacturer states that the antibody works on a wide range of

species, we tested it on T. gondii and Perkinsus in our lab.

Validation of Anti-Histone H3 antibody (Abcam): validated to work with a wide range of species according to the manufacturer, including other alveolate species such as T. gondii and dinoflagellates as tested by our lab.

Validation of Anti-mCherry antibody (Abcam): The manufacturer states this antibody to be species independent. This antibody works on alveolate organisms P. falciparum and T. gondii.

Validation of HYGROMYCIN PHOSPHOTRANSFERASE ANTIBODY (10-1421) (anti-hygromycin antibody, Fitzgerald Industries): Recombinant hygromycin phosphotransderase from E.coli

Validation of H9658- Monoclonal Anti-HA antibody produced in mouse (Sigma):
1. The quality was tested by western blot analysis in E.coli expressing HA-Tag with tiger 1:40,000 dilution
2. In whole extract of human HEK-293T cells overexpressing N-terminal HA tagged fusion protein was separated on SDS-PAGE and probed with Monoclonal Anti-HA Clone: HA7 (Cat. No. H9658).

For each primary antibody, these antibodies have been used in several independent experiments with consistent results.

Eukaryotic cell lines

Policy information about cell lines

Cell line source(s)

Protist culture collections: Ostreococcus - RCC802; Bathycoccus - RCC4222; Micromonas - CCMP2709 and CCMP 1545; Tetraselmis -KAS-836; Pyramimonas - SCCAP K-0007; Isochrysis - CCMP1323; Emiliania - CCMP1516; Amorphochlora - CCMP2058; Bigelowiella - CCMP 2755; Fragilariopsis - CCMP1102; Thalassiosira - CCMP1335; Seminavis - DCG 0498 and DCG 0514; Pseudo-nitzschia - 15091C3; Heterosigma - CCMP 2393; Aurantiochytrium - ATCC MYA-1381; Nannochloropsis - CCMP 1779; Phaeodactylum - CCAP1055/1; Chromera - CCMP 2878; Perkinsus - ATCC PRA240; Oxyrrhis - CCMP1788 and CCMP1795; Fugacium - CCMP 2468; Alexandrium - CCMP BF-5; Breviolum - NIES-4271; Crypthecodinium - CCMP316; Amphidinium - CCMP1314; Karlodinium - CCMP1975; Diplonema - ATCC50162; Eutreptiella - SCCAP K-0333; Naegleria - ATCC30224; Pirum - ATCC PR-280; Sphaeroforma - ATCC PRA-297; Abeoforma - ATCC PRA-279; Salpingoeca - ATCC PRA-390.

Authentication

Genome sequencing, sequencing of phylogenetic marker genes (e.g. 18S)

Mycoplasma contamination

The cell lines were not tested for mycoplasma contamination.

Commonly misidentified lines
(See ICLAC register)

No commonly misidentified cell lines were used.

Flow Cytometry

Plots

Confirm that:

☒ The axis labels state the marker and fluorochrome used (e.g. CD4-FITC).

☒ The axis scales are clearly visible. Include numbers along axes only for bottom left plot of group (a 'group' is an analysis of identical markers).

☒ All plots are contour plots with outliers or pseudocolor plots.

☒ A numerical value for number of cells or percentage (with statistics) is provided.

Methodology

Sample preparation

Sample preparation is fully described in the Supplementary Materials. For experiments on the stramenopile (Nannochloropsis) electroporation occurred with or without fluorescein dextran and with or without a plasmid containing the mTagBFP2 gene under different promoters. No other stains were added after electroporation prior to cytometric analysis. For experiments with the prasinophyte (Micromonas) no stains were used at any point and experiments were performed with an eGFP plasmid with promotors from Micromonas, on cells that were either electroporated or not pulsed.

Instrument

BD InFlux cell sorter

Software

BD FACS™ Software for data collection
FlowJo for data analysis of stramenopiles, WinList for analysis of prasinophytes and WinList for all display panels. (please see above for version information).

Cell population abundance

For experiments demonstrating transfection of Nannochloropsis and Micromonas in pure culture cell population abundance was determined using an in-line flow meter on the sample line and by weighing the samples before and after data collection. This information on volume run was combined with counts acquired during each run to determine the abundance per ml for each population observed (non-transfected, transfected).
Because of the small sample volumes and low abundance of target cells in samples of natural communities, it was not possible to re-test purity of sorted samples. Instead, immediately before sorting of the sample, the sorting efficiency was confirmed to >95% with 3 um UR calibration beads. With the same InFlux instrument and sorting protocols, we have previously determined that

sorted pico-cyanobacterial populations from natural samples are >99% pure, by re-running sorted samples using trigger based on the FSC channel and with voltage and threshold set to be able to detect even non-fluorescent particles with approx. 2x lower FSC than Prochlorococcus.

Gating strategy

For Micromonas: The trigger channel was Forward Scatter (FSC), to allow detection of pigmented cells (both Micromonas and Nannochloropsis are algae with natural chlorophyll fluorescence), dead cells, and cell detritus. No gates were applied in the data collection mode, only for post analysis. All cells (with plasmid in treatments either pulsed, or not pulsed) were visualized in a two parameter histogram of FSC vs Chlorophyll (692/40 nm bp). For means, the population of non-detrital particles (living cells) was gated into a second two parameter histogram of FALS vs GFP (520/35 nm bp), as well as GFP vs Chlorophyll. From there cells with eGFP fluorescence were analyzed as were cells with baseline eGFP fluorescence and the geometric mean of these fluorescences were compared, as were cell abundance in each population.
For Nannochloropsis: The trigger channel was Forward Scatter (FSC), to allow detection of pigmented cells, dead cells, and cell detritus. A first gate was drawn based on FSC and Side Scatter to include particles with the optical scattering characteristics (related approximately to size and cell complexity) of Nannochloropsis cells. Living Nannochloropsis cells maintain high red fluorescence from chlorophyll, so a second gate was drawn based on chlorophyll fluorescence (692 nm, excited by the 488 nm laser) and FSC. In samples electroporated either with (treatment) or without plasmid DNA (sham controls), the proportion of celllike
particles (based on FSC and SSC) which had chlorophyll fluorescence remained high (>95%) during the first two hours but had dropped to 72-85% at 24 hours, while in non-electroporated samples the proportion remained >95% for the first two hours. In this way, blue fluorescence related to mTagBFP2 was assessed only on cells which maintained the optical characteristics of healthy Nannochloropsis.
For natural samples: The trigger channel was FSC, with voltage and threshold set to include all particles which displayed high yellow autofluorescence (at 580 nm) and high red autofluorescence (at 692 nm), which correspond to Synechococcus-like cells with yellow fluorescent phycoerythrin. In control (not electroporated) and sham-control (electroporated without plasmid), gates were drawn based on red fluorescence (due to chlorophyll, 692 nm excited by the 640 nm laser) and FSC as shown in Supplementary Fig. 6. Spherical fluorescent calibration particles (3 um Spherotech UR) were used as guides for setting FSC parameters to define smaller picoplancton versus cells that represent larger picoplancton or nanoplancton. Three clear phytoplankton "populations" (groups of particles with tightly similar optical characteristics) were visually identified and gates were drawn around them. The gate with lowest FSC was composed of 100% or nearly 100% cells exhibiting high yellow fluorescence due to phycoerythrin, so were assigned as "Cyanobacteria". The other two populations were therefore assumed to correspond to a type of small picophytoplankton (with FSC and pulse width well below that of 3 um calibration spheres) and larger picophytoplankton. These three populations together showed a clear relationship between chlorophyll fluorescence and FSC. Cells/particles with chlorophyll fluorescence below this relationship did not form clear groups and are assumed to represent non-phytoplankton, some of which can exhibit low red fluorescence due to phytoplankton prey in food vacuoles. For each time point, the gate to define blue fluorescent (putative BFP-expressing) cells was drawn to exclude over 95% of cells in sham-controls which were electroporated with dextran green (to track electroporation) but without plasmid DNA. This gate was drawn diagonally on 460 nm (blue fluoresence) versus 530 nm (green fluorescence) to exclude any signal that could come from bleedover
of the green fluorescence of fluorescein dextran.

☒ Tick this box to confirm that a figure exemplifying the gating strategy is provided in the Supplementary Information.
