# Supplementary material for: Genetic tool development in marine protists: emerging model organisms for experimental cell biology
Source: Nat Methods. 2020 Apr 6;17(5):481–94. doi: 10.1038/s41592-020-0796-x (PMC7200600; doi:10.1038/s41592-020-0796-x)
Supplement: Supplementary file 3 — Supplementary Tables 1, 2, 4 and 5. [file 41592_2020_796_MOESM3_ESM.pdf]

Suppl. Table 1. Overview of protists for which transformation systems have been developed prior to the EMS initiative.

Phylum	Species	Transformation method	Construct (promoter)	Selectable marker; selecting agent	Reporter	Reference
**Alveolates**	*Symbiodinium microadriaticum*	Si carbide whiskers (SiCaW)	pMT Npt/GUS (nos, CaMV35S); pMT Hpt/GUS *(Agrobacterium* *[Agro]* p1'2')	*nptII, hpt*; kanamycin, hygromycin*, G418	Beta-glucuronidase (GUS)	ten Lohuis & Miller (1998)
	Symbiodinium spp.	Agitation with Glass beads	*pCB302-gfp-AtRACK1C*, *pCAMBIA-FABD2-gfp* (CaMV35S)	*bar, hpt; Basta*, hygromycin	Green fluorescent protein (GFP)	Ortiz-Matamoros et al. (2015a)
		Glass beads and co-incubation with *Agro*	pCB302-*gfp-AtRACK1C*, pCB302-*gfp-MBD*, pCB302-*gfp-FABD2* (CaMV35S)	*bar*; Basta	Green fluorescent protein (GFP)	Ortiz-Matamoros et al. (2015b)
	Amphidinium carterae	SiCaW	pMT Npt/GUS (nos, CaMV35S); pMT Hpt/GUS (*Agro* p1'2')	*nptII, hpt*; hygromycin*, G418, kanamycin	GUS	ten Lohuis & Miller (1998)
**Stramenopiles**	*Phaeodactylum tricornutum*	Biolistics	(*fcp* [fucoxanthin chlorophyll-a or -c binding protein])	*ShBle* (phleomycin/zeocin)	Luciferase (LUC)	Falciatore et al. (1999)
		Biolistics	pfcpA (fcp [fucoxanthin chlorophyll-a or -c binding protein])	*ShBle* (phleomycin/zeocin)	Chloramphenicol acetyltransferase (CAT)	Apt et al. (1996)
		Electroporation	pHY11-*cat* (native NR promoter)	*cat*; chloramphenicol	CAT	Niu et al. (2012)
		Conjugation	p0521S/CEN6-ARSH4-HIS3/pfcpA	*shble*	Cyan fluorescence protein (CFP), GFP, Yellow fluorescence protein (YFP)	Karas et al. (2015)
	Cyclotella criptica	Biolistics	plasmids with nptII (diatom acetyl-CoA carboxylase promoter)	*nptII;* G418	Expression of NPTII	Dunahay et al. (1995)

	Navicular saprophila	Biolistics	plasmids with nptII (diatom acetyl-CoA carboxylase promoter)	nptII; G419	Expression of NPTII	Dunahay et al. (1995)
	Cylindrotheca fusiformis	Biolistics	pHUPtag-*fcp* (fucoxanthin chlorophyll-a or -c binding protein)	*ble/frue*, *ble*/HUPtag; zeocin, kanamycin, hygromycin B		Fischer et al. (1999)
	Conticribra (Thalassiosira) weissflogii	Biolistics	(*fcp* [fucoxanthin chlorophyll-a or -c binding protein])		Luciferase (LUC)	Falciatore et al. (1999)
	Pseudo-nitzschia multistriata, P. arenysensis	Bioballistic	Histone H4	*Shble* , zeocin	Growth on selective medium	Sabatino et al. 2015
	Thalassiosira pseudonana	Biolistics	pTpNR/GFP (nitrate inducible NR promoter)	*Nat* (nourseothricin)	EGFP	Poulsen & Chesley (2006)
	Thalassosira pseudonana	Conjugation	pTpExpPEPCK-YFP (LHCF9 promoter_	*Nat* (nourseothricin)	Yellow fluorescent protein (YFP)	Karas et al. (2015)
	Nannochloropsis sp.	Electroporation	*pVCP1*	*shble*		Kilian et al. (2011)
	Nannochloropsis gaditana	Electroporation	pTUB/pHSP/pUEP/	*shble*		Radakovits et al. (2013)
	Nannochloropsis oceanica	Electroporation	pCMV-EM7	*sheble*		Osorio et al. (2019)
**Archaeplastids**	*Chlamydomonas reinhardtii*	Glass beads	pMN24 (containing native fragments)	*nr;* nitrate	Growth on selective medium (nitrate)	Kindle (1990)
		Electroporation	pJD67 carrying ARG7	*arg7*	Growth on corn starch	Shimogawara et al. (1998)
		Si carbide whiskers	pMN24 based on pUC19 (containing native NR)	*nr*, nitrate	Growth on selective medium (nitrate)	Dunahay (1993)
		Biolistics	pU12.6 (containing ASL)	argininosuccinate lyase (ASL); AS	Growth on selective medium	Debuchy et al. (1989)

	Biolistics	*p*UC19	OEE1, oxygen-evolving enhancer protein 1	Growth on selective medium (-acetate)	Mayfield & Kindle (1990)
	*Agrobacterium tumefaciens***	T-DNA	*hpt*; hygromycin	GUS, GFP	Kumar et al. (2004)
Chlorella ellipsoidea	Biolistics	*p*DO432		LUC	Jarvis & Brown (1991)
	Electroporation	(Ubil-Ω)		GUS	Chen et al. (2001)
Chlorella saccharophila	Electroporation	PBI221 (CaMVS35)		GUS	Maruyama et al. (1994)
Chlorella vulgaris	Protoplast transformation		*kanr*; G418	Expression of human growth hormone (hGH)	Hawkins & Nakamura (1999)
	Electroporation	pMD18-Ap*cat* (NR promoter)	*cat*; chloramphenicol		Niu et al. (2011)
Haematococcus pluvialis	Biolistics	*p*SV40-*LacZ* (SV40)		β-galactosidase (LacZ)	Teng et al. (2002)
Ostreococcus tauri	Electroporation	*Potluc*<br>*Potox*	KanMX, G418<br>Nat1, cloNat	LUC	Corellou et al. [2009]
	Electroporation	PCR product including 1kbp of ferritin homologous sequence	KanMx, G418	LUC<br>(Knock in)	Lozano et al. [2014]
Dunaliella salina	Electroporation	PBI221, pUGUS, pUΩGUS, P35UΩGUS (CaMVS35, Ubil-Ω)		GUS	Geng et al. (2003)
	Electroporation		*ble*; Zeocin		Sun et al. (2005)***
	Biolistics	(CaMV35S promoter)	*bar*; Basta	GUS	Tan et al. (2005)
	Biolistics	*p*DM307 (native CA [carbonic anhydrase] promoter)	*bar*; Basta	Nitric oxide synthase (NOS)	Lü et al. (2005)

*found to be most effective; ** shown to work better than glass beads; *** some introduced DNA stayed as episomal plasmid DNA

References for Suppl. Table 1:

ten Lohuis. M. R. & Miller, D. J. Genetic transformation of dinoflagellates (*Amphidinium* and *Symbiodinium*): expression of GUS in microalgae using heterologous promoter constructs. *Plant J*. **13**, 427-435. (1998).

Ortiz-Matamoros, M. F., Villanueva, M. A., & Islas-Flores, T. Transient transformation of cultured photosynthetic dinoflagellates (*Symbiodinium* spp.) with plant-targeted vectors. *Cienc. Mar.* **41**, 21-32. (2015a).

Ortiz-Matamoros, M. F., et al. Heterologous DNA Uptake in Cultured *Symbiodinium* spp. Aided by *Agrobacterium tumefaciens*. *PLoS ONE* **10**, e0132693. (2015b).
Falciatore, A., Casotti, R., Leblanc, C., Abrescia, C. & Bowler, C. Transformation of nonselectable reporter genes in marine diatoms. *Mar. Biotechnol.* **1**, 239-251 (1999).

Apt, K. E., Kroth-Pancic, P. G. & Grossman, A. R. Stable nuclear transformation of the diatom *Phaeodactylum tricornutum*. *Mol. Gen. Genet.* **252**, 572-579 (1996).

Niu, Y.-F., et al. Transformation of diatom *Phaeodactylum tricornutum* by electroporation and establishment of inducible selection marker. *BioTechniques* **52**, 1-3 (2012).

Karas, B. J. et al. Designer diatom episomes delivered by bacterial conjugation. *Nat. Commun.* **6**, 6925 (2015).

Dunahay, T., Jarvis, E. & Roessler, P. Genetic transformation of the diatoms *Cyclotella cryptica* and *Navicula saprophita*. *J. Phycol.* **31**, 1004**–**1012 (1995).

Fischer, H., Robl, I., Sumper, M. & Kröger, N. Targeting and covalent modification of cell wall and membrane proteins heterologously expressed in the diatom *Cylindrotheca fusiformis. J. Phycol.* **35**, 113-120 (1999).

Falciatore, A., Casotti, R., Leblanc, C., Abrescia, C. & Bowler, C. Transformation of nonselectable reporter genes in marine diatoms. *Mar. Biotechnol.* **1**, 239-251 (1999).

Sabatino, V., Russo M. T., Patil S., d'Ippolito G., Fontana A., and Ferrante M. I. Establishment of genetic transformation in the sexually reproducing diatoms *Pseudo-nitzschia multistriata* and *Pseudo-nitzschia arenysensis* and inheritance of the transgene. *Marine Biotechnology* **17**: 452-462 (2015).

Poulsen, N., Chesley, P. M. & Kröger, N. Molecular genetic manipulation of the diatom *Thalassiosira pseudonada* (Bacillariophyceae). *J. Phycol.* **42**, 1059–1065 (2006).

Kilian, O,, et al. High-efficiency homologous recombination in the oil-producing alga *Nannochloropsis* sp. *Proc. Natl. Acad. Sci. USA* **108**, 21265–21269 (2011)

Radakovits, R. et al. Draft genome sequence and genetic transformation of the oleaginous alga *Nannochloropsis gaditana*. *Nature Communications* **3**, 686 (2013).

Osorio, H., et al. High-efficiency nuclear transformation of the microalgae *Nannochloropsis oceanica* using Tn5 Transposome for the generation of altered lipid accumulation phenotypes. *Biotechnol. Biofuels* **12**, 134 (2019).

Kindle, K. L. High-frequency nuclear transformation of *Chlamydomonas reinhardtii. Proc. Natl. Acad. Sci. USA* **87**, 1228-1232 (1990).

Shimogawara, K., Fujiwara, S., Grossman, A. and Usuda, H. High-efficiency transformation of *Chlamydomonas reinhardtii* by electroporation. *Genetics*, **148**, 1821-1828 (1998).

Dunahay, T. G. Transformation of *Chlamydomonas reinhardtii* with silicon carbide whiskers. *BioTechniques* **15**, 452-460 (1993).

Debuchy, R., Purton, S. and Rochaix, J.D. The argininosuccinate lyase gene of *Chlamydomonas reinhardtii*: an important tool for nuclear transformation and for correlating the genetic and molecular maps of the ARG7 locus. *The EMBO journal*, **8**, 2803-2809 (1989).

Mayfield, S. P., Kindle, K. L. Stable nuclear transformation of *Chlamydomonas reinhardtii* by using a *C. reinhardtii* gene as the selectable marker. *Proc. Natl. Acad. Sci. USA* **87**, 2087-2091 (1990).

Kumar, S. C., Misqitta, R. W., Reddy, V. S., Rao, J. B. & Rajam, M. V. Genetic transformation of the green alga *Chlamydomonas reinhardtii* by *Agrobacterium tumefaciens*. *Plant Sci*. **166**, 731-738 (2004).

Jarvis, E.E. and Brown, L.M. Transient expression of firefly luciferase in protoplasts of the green alga *Chlorella ellipsoidea*. *Current genetics*, **19**, 317-321. (1991).

Chen, Y., Wang, Y., Sun, Y., Zhang, L. and Li, W. Highly efficient expression of rabbit neutrophil peptide-1 gene in *Chlorella ellipsoidea* cells. *Curr.t Genet.*, **39,** 365-370 (2001).

Maruyama, M., et al. Introduction of foreign DNA into *Chlorella saccharophila* by electroporation. *Biotechnol. Techn.* **8**, 821-826 (1994).

Hawkins, R.L. and Nakamura, M. Expression of human growth hormone by the eukaryotic alga, *Chlorella*. *Curr. Microbiol.*, **38**, 335-341 (1999).

Niu, Y. F., et al. A new inducible expression system in a transformed green alga, *Chlorella vulgaris*. *Genet. Mol. Res.* **10**, 3427-3434 (2011).

Teng, C., et al. Transient expression of lacZ in bombarded unicellular green alga *Haematococcus pluvialis*. *J. Appl. Phycol.*, **14,** 497-500 (2002).

Corellou et al. Clocks in the green lineage: comparative functional analysis of the circadian architecture of the picoeukaryote *Ostreococcus*. Plant Cell, **21,** 3436-3449 (2009).

Lozano et al. Efficient gene targeting and removal of foreign DNA by homologous recombination in the picoeukaryote *Ostreococcus*. Plant J. **78**, 1073-1083. (2014).

Geng, D., Wang, Y., Wang, P., Li, W. & Sun, Y. Stable expression of hepatitis B surface antigen gene in *Dunaliella salina* (Chlorophyta). *J. Appl. Phycol.* **15**, 451-456 (2003).

Sun, Y., et al. Expression of foreign genes in *Dunaliella* by electroporation. *Mol. Biotechnol*. **30**, 185-192 (2005).

Tan, C., Quin, S., Zhang, Q., Jiang, P. & Zhao, F. Establishment of a micro-particle bombardment transformation system for *Dunaliella salina*. *J. Microbiol*. **43**, 361-365 (2005).

Lü, Y. M., et al. Cloning and functional analyses of promoters of two carbonic anhydrase genes from *Dunaliella salina*. *Acta Genet. Sin.* **31**, 1157-1166 (2004).

Suppl. Table 2. Physiological and ecological properties of protists selected for this study.

Species	Lifestyle (auto/hetero/mixo)	Axenic culture	Habitat	Cell wall	Cell structure	Life cycle	Planktonic
Archaeplastids							
Ostreococcus lucimarinus	photoautotroph	no	marine	no visible cell wall, just thin membrane	small (~1 μm); atypical prasinophyte (no flagella, no scales)	asexual by binary fission, possible sexual cycle	yes
Bathycoccus prasinos	photoautotroph	no	marine	organic scales	small (~2-3 μm); cells covered by scales of unknown organic material. no flagellum.	asexual by binary fission, possible sexual cycle	yes
Micromonas commoda	photoautotroph	yes (Rendered axenic using antibiotics for Worden et al. Science 2009. Tested here at start and end of each experiment using DAPI and test medium.)	marine	no visible cell wall, just thin membrane	small (~1-2 μm); single flagellum, no scales; phototactic (positive)	asexual by binary fission, possible sexual cycle	yes
Micromonas pusilla	photoautotroph	yes (Rendered axenic using antibiotics for Worden et al. Science 2009. Tested here at start and end of each experiment using DAPI and test medium.)	marine	no visible cell wall, just thin membrane	small (~1-2 μm); single flagellum; phototactic (positive); Encodes most of peptidoglycan pathway from cyanobacterial endosymbiont that formed plastid	asexual by binary fission, possible sexual cycle	yes
Tetraselmis striata	photoautotroph	no	marine coastal; most free-living but some animal symbionts	complex polysaccharide scales fuse to form a wall; cell anchored to wall by microtublues in four places	3-25 μm, two pairs of flagella with complex scales etc covering, one large chloroplast; accumulates HUFAs	flagellated stage, vegetative no-motile stage (usually dominant), cyst	yes
Pyramimonas parkeae	photoautotroph	not tested	marine	wall-less	20 x 15 μm	four flagella	yes
Haptophytans							
Isochrysis galbana	photoautotrophic	no	marine	thin layer of organic scales, non-calcified	5-6μm, two flagella, 2 chloroplasts, stigma, oil droplets	Asexual by binary fission, cyst	yes

Emiliana huxleyi	photoautotrophic	no	marine, broadly distributed	calcium carbonate plates (coccoliths)	~5 µm, no flagella	diploid calcified stage, motile non-calcified haploid stage	yes
Rhizarians							
Amorphochlora (Lotharella) amoebiformis	photomixotrophic (eat bacteria)	no	marine	none	Amoeboid cell (8-15 µm) with many filopodia.	asexual by binary fission	no
Bigelowiella natans	photomixotrophic (eat bacteria)	not tested	marine, broadly distributed	vegetative cells usually naked; this cell wall only in 'cysts' in old cultures	Amoeboid, thin filopodia (thread-like pseudopodia; sometimes reticulopodia); or coccoid cells with multilayered cell wall; or uniflagellated zoospores. store b-1,3 glucan (not starch). Mitochondria with tubular cristae. Nucleomorph genome ~400 kb (3 linear chromosomes), coding 17 plastid genes and ~350 housekeeping genes with lots of small (~20 bp) introns. Secondary endosymbiosis between cercozoan host and green algal symbiont	some species have all three cell types, many are missing one or another; which form is the main vegetative stage differs among species. Chlorarachnion may have amoeboid gamete!	yes
Stramenopiles							
Fragilariopsis cylindrus	photoautotrophic	not tested	polar oceans and sea ice; ice edge blooms	silica	~4 µm, pennate		yes
Thalassiosira pseudonana	photoautotrophic	yes (Bacterial growth checked in LB plates)	oceanic, coastal temperate, possible bacterial symbiont	silica and organics (long-chain polyamines, chitin, proteins)	~5-10 µm	size reduction–restitution cycle (SRRC): asexual by binary fission accompanied by cell size reduction, cell size restored in a sexual cycle	yes
Seminavis robusta	photoautotrophic	not tested, but maintained with a cocktail of Penicillin, Ampicillin, Gentamycin, and Streptomycin	benthic	silica	~10x50 µm; pennate	Size reduction-restitution cycle; sexual cycle with two known mating types	no
Pseudo-nitzscha multiseries	photoautotrophic	no	HAB-forming; common during coastal upwelling; significant	silica	~5x100 µm; pennate, chain-forming	asexual by binary fission accompanied by cell size reduction, cell	yes

			player in local food web; can be toxic; neretic to open ocean			size restored in a sexual cycle	
Heterosigma akashiwo	photoautotrophic	no	diverse; some form HAB	naked	~50-100 µm, heterokont flagella		yes
Aurantiochytrium limacinum	osmoheterotrophic; stores PUFAS	yes (checked by 16S PCR, by microscopy, also, neither genomic nor transcriptomic sequencing have suggested the presence of any other organism)	marine (plant detritus)	sulfated polysaccharide scales	4 - 20 µm; grow attached by ectoplasmic net elements or in suspension	probably asexual reproduction by zooospores	mero[1]
Caecitellus sp.	phagoheterotrophic	not tested		none?	<5 µm; gliding motility, raptorial feeding		yes
Nannochloropsis oceanica	photoautotroph	not tested (bought as axenic, but not retested)	marine waters worldwide	Smooth cell wall, composed of principally cellulose and algaenan, with papilla	<5 µm ovoid cells, non-motile	asexual reproduction; may be ameiotic	yes
Phaeodactylum tricornutum	photoautotrophic	yes (Provasoli antibiotic treatment, DAPI staining)	brackish and marine waters worldwide	oval morphotype silicified, other morphotypes very lightly silicified, sulfated a-mannan decorated with glucuronic residues	polymorphic (exists as oval, fusiform, triradiate, cruciform morphotype), ~5x30 µm (fusiform), ~10 µm ovoiate gliding motility (oval), can form chains	asexual by binary fission, possible sexual cycle	yes (oval type benthic)
Alveolates							
Euplotes crassus	phagoheterotrophic	no	marine	rigid pellicle	~50-100 µm; hypotrichous ciliate	asexual by binary fission and sexual by conjugation or autogamy	yes
Euplotes focardi	phagoheterotrophic	no	marine f=antarctic	rigid pellicle	~50-100 µm; hypotrichous ciliate	asexual by binary fission and sexual by conjugation	yes
Chromera velia	photoautotroph?	not tested	coral reef coral-associated, possibly non-facultative symbiont	thick cell wall	7.0 × 7.6 µm, coccoid stage with no flagella	sex not observed, forms flagellate stages	mero[1]
Perkinsus marinus	osmoheterotroph, obligate parasite	yes (The ATCC repository sells it as axenic)	parasite of marine molluscs	Yes	2-10 µm; suggested apicoplast	direct cycle, zoospores and	Both as trophozoite and

						trophozoites, sex not observed	flagellated stage
Oxyrrhis marina	heterotrophic (phagotrophic; omnivorous)	no	plankton; global coastally except polar; can form blooms	naked (no theca) except has scales	20-30 μm		yes
Hematodinium sp.	parasite	yes	crustacean hemolymph	naked at trophont stage	Cultured as asexual multinucleated trophonts (up to 50 μm)	Complex asexual stages, non-flagellate; mononucleated sexual zoospores	Only at zoospore stage
Fugacium (Symbiodinium) kawagutii	photoautotrophic symbionts (maybe some phagotrophy?)	no (Grown under 100 μg/ml Ampicillin, 50 μg /ml Kanamycin and 50 μg/ml Streptomycin)	intra- or intercellular symbionts of marine invertebrates	naked swimming cell but cellulose wall in nonmotile phase	~10 μm	motile (free-swimming) and non-motile (in host) phases; only the latter grows and divides	
Alexandrium catenella	photoautotroph	no (Grown under 100 μg/ml Ampicillin, 50 μg /ml Kanamycin and 50 μg/ml Streptomycin)	HAB-forming phytoplankton; produce saxitoxin (PSP)	cellulose plates	~25 μm, usually chains of 2, 4, 8	asexual reproduction by binary fission; sexual reproduction to form a cyst	yes
Breviolum (Symbiodinium) sp.	photoautotrophic symbionts (maybe some phagotrophy?)	not tested	intra- or intercellular symbionts of marine invertebrates	naked swimming cell but cellulose wall in nonmotile phase	~10 μm	motile (free-swimming) and non-motile (in host) phases; only the latter grows and divides	
Crypthecodinium cohnii	heterotrophic (glucose, acetate, propionic acid); may predate via peduncle	yes (The NCMA repository sells it as axenic; the samples are routinely tested for bacterial growth in marine liquid bacterial medium)	brackish, littoral, neritic; often around macrophytes like Fucus. temperate and tropical	very thin cellulose	~15-20 μm; produce carotenoids in the light	stores starch during log phase, DHA mainly in cysts?; swimming cells and cysts.	
Amphidinium carterae	photoautotroph	no (wild-type is not axenic, but can be grown axenically)	HAB-forming phytoplankton	naked?	~25 μm, solitary	Asexual by binary fission	yes
Karlodinium veneficum	mixotroph (predatory)	no	HAB-forming, toxic, marine, planktonic	naked	~10 μm		yes

		(Grown under 400 µg/ml Ampicillin)					
Discobans							
Bodo saltans	phagoheterotrophic	no (the cultures which carried bacterial populations from the original isolation were inoculated with bacteria (Klebsiella pseudomonas) as a food source	broadly distributed	none?	*B. saltans* is 4-5 µm with two flagellae		no? *B. saltans* attaches to surfaces by tip of long (posterior) flagellum
Diplonema papillatum	phagoheterotrophic	yes (checked by microscopy, 100µg/ml of chloramphenicol added in the media)	free-living planktonic?	no pellica	~16 µm, two flagella of equal lenth, two subapical openings		yes
Eutreptiella gymnastica	photoautotrophic	not tested	neritic, cosmopolitan	flexible pellicle	15-20 µm, two flagella, reddish eyespot, paramylon granules in cytoplasm, vigorous metaboly often observed	can form a cyst with layered cell wall	yes
Naegleria gruberi	phagoheterotrophic	yes (Axenic culture was tested microscopically along with DAPI straining).	wet soil and freshwater	naked	amoeboflagellate; the amoeba lacks microtubule cytoskeleton, flagellate has elaborate one including the flagella; de novo synthesis of basal body during transformation from former to latter	apparently the genome revealed two distinct haplotypes	no
Opisthokonts							
Pirum gemmata	osmoheterotroph; stores glycogen	yes (Sold axenic by ATCC)	peanut worm (*Phascolosoma agassizii*) gut contents, BC, 2004	composition unknown but fibrous and woven; contain membrane-bound tubular extensions of the cytoplasm with tubules.	vegetative cells ~50-150 µm; large central vacuole with cytoplasm mostly pressed against periphery; multinucleate with nuclei 2 to 4 µm; sporulation happens in half an hour; endospores ~5 µm and 'weakly amoeboid'	walled cells divide internally to produce lots of endospores, which are released through parental cell wall in spurts	no
Sphaeroforma arctica	phagoheterotrophic	yes (axenic growth checked in agar plates)	invertebrate symbiont; isolated from Gammarus; arctic	carbohydrate, mostly N-acetyl-glucosamine (chitin?)	simple round cells, lots of DHA and EPA in cell membranes but not accumulated to high levels in lipid bodies	very simple growth from 5-7 µm cells for 48 hr to 35-40 µm, then	no

						releasing ~120 new cells	
Abeoforma whisleri	osmoheterotroph; stores glycogen	yes (axenic growth checked in agar plates)	mussel (*Mytilus* sp.) gut contents, BC, 2007	have both cell wall and extracellular matrix; wall sometimes very thick; composition unknown but fibrous and woven; contain membrane-bound tubular extensions of the cytoplasm with tubules.	vegetative cells mostly spherical ~50 µm but some plasmodial; endospores, plasmodia, and hyphae-like structures observed; say dispersal amoebae 'function in post reproductive dispersal' and are uninucleate	walled spherical cells, plasmodia, amoebae; asexual reproduction by dispersal amoebae, endospores, binary fission and budding.	no
Salpingoeca rosetta	phagoheterotrophic	no	free-living planktonic and benthic thecate	a proteinaceous and polysaccharide matrix	cell body 3-10 µm; single apical flagellum surrounded by a collar of 30-40 actin-filled microvilli	asexual by longitudinal fission; sexual reproduction triggered by nutrient deprivation and a secreted chondroitin lyase from *Vibrio* bacteria; dynamic life history includes unicellular and multicellular forms	yes

[1] Meroplanktonic organisms spend only a portion of their lives as plankton.

Suppl. Table 4. Transformation methods applied in this study.
A. Electroporation, B. Biolistics, C. Microinjection, D. Chemical transformation, E. Conjugation and F. Glass bead abrasion conditions.

A. Electroporation

Species	Electroporation devices (type of transformation)	Used program/setting	Survival rate
**Archaeplastids**			
Ostreococcus lucimarinus	Gene Pulser Xcell	1200V, 25µF	30-50%
Bathycoccus prasinos	Gene Pulser Xcell	1500V, 25µF	50%
Micromonas commoda	LONZA®	SF Buffer with pulse EH-100, EO-100, EN-138 and EW-113	10-11%
Micromonas pusilla	BioRad Gene Pulser	1000V, 10µF, 400Ω<br>800V, 25µF,400Ω<br>600V, 50µF,400Ω	n/a
Pyramimonas parkeae	Gene Pulser Xcell	1500V, 25µF	n/a
	Gene Pulser Xcell	300V, 500µF	n/a
	Gene Pulser Xcell	2500V, 25µF	n/a
	Gene Pulser Xcell	310V, 960µF	n/a
	Gene Pulser Xcell	420V, 960µF	n/a
	Gene Pulser Xcell	300V, 200µF	n/a
	Gene Pulser Xcell	100V, 100µF	n/a
	Gene Pulser Xcell	100V, 300µF	n/a
	Amaxa Nucleofector II	preset program X-001	n/a
	Amaxa Nucleofector II	preset program T-020	n/a
	Amaxa Nucleofector II	preset program T-023	n/a
	Amaxa Nucleofector II	preset program U-035	n/a
**Rhizarians (Chlorarachniophytes)**			
Amorphochlora (Lotharella) amoebiformis	Gene Pulser Xcell	120 V, 25 ms square wave, 0.2 cm cuvette	20-30%
Bigelowiella natans	Amaxa Nucleofector II	preset program X-001	n/a
	Amaxa Nucleofector II	preset program T-020	n/a
	Amaxa Nucleofector II	preset program T-023	n/a
	Amaxa Nucleofector II	preset program U-035	n/a
	Gene Pulser Xcell	7× poring pulse: 300V, 5ms; 5× transfer pulse: 10V, 50ms	n/a
	Gene Pulser Xcell	7× poring pulse: 250V, 5ms; 5× transfer pulse: 10V, 50ms	n/a
	Gene Pulser Xcell	7× poring pulse: 350V, 5ms; 5× transfer pulse: 10V, 50ms	n/a
**Stramenopiles (Diatoms, Bacillariophytes, Raphidophytes)**			

Seminavis robusta	BTX ECM 2001	Single pulse conditions: 5 V AC for 5 sec, 300 V pulse for 5, 2.5, 0.5, or 0.1 msec.<br>5 pulse conditions: 5 V AC for 5 sec, 300 V pulse for 5, 2, 1, or 0.1 msec<br>10 pulse conditions: 5 V AC for 5 sec, 300 or 500 V pulse for 0.1 or 0.05 msec (only for 500 V)	n/a
	NEPA21	Poring pulse: 150, 200, 225, 250, 275, or 300 V, 10% decay rate; 5 ms length, 50 ms interval<br>Transfer pulse: 8 V, 40% decay rate, 50 ms length, 50 ms interval, alternating polarity	n/a
Heterosigma akashiwo	Gene Pulser Xcell	50/75 V, 25 μF and ∞Ω	80%
Aurantiochytrium limacinum	Gene Pulser (BIO-RAD)	2 pulses: 450V, 25 μF, 1000 Ω (~5 ms)	2.64%
	NEPA	2 pulses: 250 V, 4 ms	2.48%
	NEPA	2 pulses: 275 V, 8 ms	4.13%
	NEPA	2 pulses: 300 V, 12 ms	7.27%
Nannochloropsis oceanica	Gene Pulser II	1800V, 50 μF, 20 ms	n/a
	Gene Pulser II	1000V, 50 μF, 20 ms	n/a
**Alveolates**			
Euplotes crassus	BioRad Gene Pulser	200V, 25μF, 100Ω	50-60%
Chromera velia	Amaxa Nucleofector II	preset programs X-001	n/a
	Amaxa Nucleofector II	preset program T-020	n/a
	Amaxa Nucleofector II	preset program T-023	n/a
	Gene Pulser Xcell	1500V, 25μF	n/a
	Gene Pulser Xcell	300V, 500μF	n/a
	Gene Pulser Xcell	2500V, 25μF	n/a
	Gene Pulser Xcell	310V, 960μF	n/a
Perkinsus marinus	Amaxa Nucleofector II	preset program D-023	n/a
Oxyrrhis marina	BioRad's MicroPulser	DIC	n/a
	BioRad's MicroPulser	DIC -2 shocks	n/a
	BioRad's MicroPulser	SHS	n/a
	BioRad's MicroPulser	SC2	n/a
Hematodinium sp.	Amaxa Nucleofector II	D-023 and X-001	<1%
Fugacium kawagutii	BioRad's MicroPulser	DIC	n/a
	BioRad's MicroPulser	DIC -2 shocks	n/a
	BioRad's MicroPulser	SHS	n/a

	BioRad's MicroPulser	SC2	n/a
Alexandrium catenella	BioRad's MicroPulser	DIC	n/a
	BioRad's MicroPulser	DIC -2 shocks	n/a
	BioRad's MicroPulser	SHS	n/a
	BioRad's MicroPulser	SC2	n/a
Breviolum (Symbiodinium) sp.	NePA21 Electro-kinetic transfection system	Poring pulse: 300V, 10ms<br>Transfer pulse: 8V, 50ms	n/a
Crypthecodinium cohnii	Amaxa Nucleofector II	preset program D-023	79%
	Amaxa Nucleofector II	preset program A-020	85%
	Amaxa Nucleofector II	preset program T-020	67%
	Amaxa Nucleofector II	preset program T-030	53%
	Amaxa Nucleofector II	preset program X-001	62%
	Amaxa Nucleofector II	preset program X-001	71%
	Amaxa Nucleofector II	preset program L-029	77%
	Amaxa Nucleofector II	preset program X-003	n/a
	Amaxa Nucleofector II	preset program X-005	n/a
	Amaxa Nucleofector II	preset program X-033	n/a
	Amaxa Nucleofector II	preset program Y-003	n/a
	Amaxa Nucleofector II	preset program Y-005	n/a
	Amaxa Nucleofector II	preset program Y-033	n/a
	Amaxa Nucleofector II	preset program Z-007	n/a
	Amaxa Nucleofector II	preset program Z-023	n/a
	Amaxa Nucleofector II	preset program Z032	n/a
	Amaxa Nucleofector II	preset program BAC1	n/a
	Amaxa Nucleofector II	preset program BAC2	n/a
	Amaxa Nucleofector II	preset program BAC3	n/a
	Amaxa Nucleofector II	preset program BAC4	n/a
	Amaxa Nucleofector II	preset program BAC5	n/a
	Amaxa Nucleofector II	preset program BAC6	n/a
	Amaxa Nucleofector II	preset program BAC7	n/a
	Amaxa Nucleofector II	preset program L-029	n/a
	Amaxa Nucleofector II	preset program L-029	n/a
	Microfluidics	Straight 313 V	n/a
	Microfluidics	Straight 625 V	n/a
	Microfluidics	Straight 938 V	n/a
	Microfluidics	Straight 1250V	n/a
	Microfluidics	Straight 1563 V	n/a
	Microfluidics	Divergent 333 V	n/a
	Microfluidics	Divergent 667 V	n/a
	Microfluidics	Divergent 1000 V	n/a
	Microfluidics	Divergent 1333 V	n/a
	Microfluidics	Divergent 1667 V	n/a

	Microfluidics	Divergent 5000 V	n/a
	Lipofectamine		90-100%
Amphidinium carterae	Amaxa Nucleofector 4D	X-100, D-023, L-029 and EH 100.	n/a
	NEPA electroporator	Poring pulse: 150V, length: 5 ms, interval 50 ms, number 7<br>Transfer pulse: 8V, length 50 ms, interval 50 ms, number 5	n/a
	NEPA electroporator	Poring pulse: 200V, length: 5 ms, interval 50 ms, number 7<br>Transfer pulse: 8V, length 50 ms, interval 50 ms, number 5	n/a
	NEPA electroporator	Poring pulse: 225V, length: 5 ms, interval 50 ms, number 7<br>Transfer pulse: 8V, length 50 ms, interval 50 ms, number 5	n/a
	NEPA electroporator	Poring pulse: 250V, length: 5 ms, interval 50 ms, number 7<br>Transfer pulse: 8V, length 50 ms, interval 50 ms, number 5	n/a
	NEPA electroporator	Poring pulse: 275V, length: 5 ms, interval 50 ms, number 7<br>Transfer pulse: 8V, length 50 ms, interval 50 ms, number 5	n/a
	NEPA electroporator	Poring pulse: 300V, length: 5 ms, interval 50 ms, number 1<br>Transfer pulse: 8V, length 50 ms, interval 50 ms, number 5	n/a
	NEPA electroporator	Poring pulse: 300V, length: 5 ms, interval 50 ms, number 4<br>Transfer pulse: 8V, length 50 ms, interval 50 ms, number 5	n/a
	NEPA electroporator	Poring pulse: 300V, length: 5 ms, interval 50 ms, number 7<br>Transfer pulse: 8V, length 50 ms, interval 50 ms, number 5	n/a
	NEPA electroporator	Poring pulse: 300V, length: 5 ms, interval 50 ms, number 9<br>Transfer pulse: 8V, length 50 ms, interval 50 ms, number 5	n/a
Karlodinium veneficum	BioRad's MicroPulser	DIC	n/a
	BioRad's MicroPulser	DIC -2 shocks	n/a
	BioRad's MicroPulser	SHS	n/a
	BioRad's MicroPulser	SC2	n/a
**Discobans (Euglenozoans and Heteroloboseans)**			
Bodo saltans	NePA21 Electro-kinetic transfection system	Poring pulse: 250V, 25ms<br>Transfer pulse: 60V, 99ms	30-50%
Diplonema papillatum	BTX	1600V, 25W, 50mF	10 %
	Amaxa Nucleofector II	preset program X-001	80-90 %
	Amaxa Nucleofector II	preset program X-014	40-50 %

Eutreptiella gymnastica	Gene Pulser Xcell	1500V, 25µF	n/a
	Gene Pulser Xcell	300V, 500µF	n/a
	Gene Pulser Xcell	2500V, 25µF	n/a
	Gene Pulser Xcell	310V, 960µF	n/a
	Gene Pulser Xcell	420V, 960µF	n/a
	Gene Pulser Xcell	300V, 200µF	n/a
	Gene Pulser Xcell	100V, 100µF	n/a
	Gene Pulser Xcell	100V, 300µF	n/a
	Gene Pulser Xcell	200V, 100µF	n/a
	Gene Pulser Xcell	200V, 300µF	n/a
	Gene Pulser Xcell	350V, 1000µF	n/a
	Gene Pulser Xcell	7× poring pulse: 300V, 5ms; 5× transfer pulse: 10V, 50ms	n/a
	Gene Pulser Xcell	7× poring pulse: 250V, 5ms; 5× transfer pulse: 10V, 50ms	n/a
	Amaxa Nucleofector II	preset program X-001	n/a
	Amaxa Nucleofector II	preset program T-020	n/a
	Amaxa Nucleofector II	preset program T-023	n/a
Naegleria gruberi	BioRad Gene Pulser xCell	175V, 500µF, 400Ω	10-20 %
	Amaxa Nucleofector II	preset program X-29	40-50%
**Opisthokonts**			
Sphaeroforma arctica	Neon	1000-2500V, 10-40 ms, 1-3 pulses	n/a (not successful)
	Lipofectamina		n/a (not successful)
	LONZA®	16 preset codes P3/P4/P5 buffer	n/a (not successful)
Abeoforma whisleri	Neon (invitrogen)	1300V, 25ms, pulse	60%
	LONZA®	preset program EN-138 P3 buffer	70%
	CaCl+Glycerol		n/a (not successful)
	Lipofectamine		100% (not successful)
Salpingoeca rosetta	LONZA®	SF Buffer with pulse CM156	50%

B. Biolistics

Species	Biolistics device	Settings	Survival rate
**Archaeplastids**			
Tetraselmis striata	Bio-Rad Biolistic PDS-1000/He Particle Delivery System	0.6µm AuNPs, rupture disc 1550 or 2000 psi, 6cm gap	n/a
Pyramimonas parkeae	PDS-1000/He	0.6 or 1µm AuNPs, rupture disc 1350 psi, 6cm gap	n/a

**Haptophytes**			
Isochrysis galbana	PDS-1000/He	0.7μm Tungsten beads rupture disc 1350 psi, 6cm gap	n/a
**Rhizarians (Chlorarachniophytes)**			
Amorphochlora (Lotharella) amoebiformis	PDS-1000/He	1 μm AuNPs, rupture disc 450 psi, 4cm gap	n/a
Bigelowiella natans	PDS-1000/He	0.6μm AuNPs, rupture disc 1350 psi, 6cm gap	n/a
**Stramenopiles (Diatoms, Bacillariophytes, Raphidophytes)**			
Fragilariopsis cylindrus	PDS-1000/He	0.7μm Tungsten beads, rupture disc 1550 psi, 6cm gap	n/a
Seminavis robusta	PDS-1000/He	0.55 μm AuNPs or 1.1 μm WNPs, 1550 psi, 3 μg/mL DNA non-linearized and linearized (but not CIP-treated), at 3, 6, 9, and 12 cm gap distances	n/a
**Alveolates**			
Euplotes crassus	Bio-Rad Biolistic PDS-1000/He Particle Delivery System	0.6 μm or 1.6 μm AuNPs, rupture disk 1550 psi, helium pressure 1750 psi, vacuum 26 inches Hg, gap distance ⅜ inches, in 10 mM HEPES pH 7.4	80-90%
Chromera velia	PDS-1000/He	0.6μm AuNPs, rupture disc 1350 psi, 6cm gap	n/a
Hematodinium sp.	Bio-Rad Biolistic PDS-1000/He Particle Delivery System	rupture disk 1550 psi<br>550 nm diameter gold particles	10%
*Fugacium kawagutii**	Bio-Rad Biolistic PDS-1000/He Particle Delivery System	0.7 or 1.1 μm Tungsten; rupture disc 450, 650, 900, 1100, 1350, 1550 psi; vacuum 28 inches Hg; 7.5cm gap	n/a
Alexandrium catenella*	Bio-Rad Biolistic PDS-1000/He Particle Delivery System	0.7 or 1.1 μm Tungsten; rupture disc 450, 650, 900, 1100, 1350, 1550 psi; vacuum 28 inches Hg; 7.5cm gap	n/a
Crypthecodiniu m cohnii	Bio-Rad Biolistic PDS-1000/He Particle Delivery System	rupture disk 1550 psi<br>550 nm diameter gold particles	70-80%
Amphidium carterae	Bio-Rad Biolistics PDS-1000/He	rupture disk 1550 psi<br>550 nm diameter gold particles	n.d.
**Discobans (Euglenozoans and Heteroloboseans)**			
Eutreptiella gymnastica	PDS-1000/He	0.6 or 1μm AuNPs, rupture disc 1350 psi, 6cm gap	n/a

*This part of work was assisted by Kaidian Zhang from Xiamen University, China.

C. Microinjection

Species	Microinjection device	Used setting	Survival rate
**Alveolates**			
Euplotes crassus	Eppendorf InjectMan NI 2	With Eppendorf Femtotips Microinjection Capillary Tip	2-10%

D. Chemical transformation

Species	Transfection reagent	Used setting	Survival rate
**Archaeplastids**			
Pyramimonas parkeae	Lipofectamine® 3000 Transfection Reagent (Invitrogen)	DNA–Lipofectamine complex prepared according to the supplier.	n/a
**Rhizarians (Chlorarachniophytes)**			
Bigelowiella natans	Lipofectamine® 3000 Transfection Reagent (Invitrogen)	DNA–Lipofectamine complex prepared according to the supplier.	n/a
**Alveolates**			
Euplotes crassus	Lipofectamine® 2000 or Lipofectamine® 3000 Transfection Reagent (Invitrogen)	DNA–Lipofectamine complex prepared according to the supplier.	100%
	Effectene Transfection Reagent (QIAGEN)	DNA–Effectene complex prepared according to the supplier with a double amount of DNA	10-20%
	FuGENE HD Transfection Reagent (Promega)	DNA–FuGENE complex prepared according to the supplier.	50-60%
**Discobans (Euglenozoans and Heteroloboseans)**			
Eutreptiella gymnastica	Lipofectamine® 3000 Transfection Reagent (Invitrogen)	DNA–Lipofectamine complex prepared according to the supplier.	n/a

E. Conjugation

Species	Coincugation (species co-incubated)	Survival rate	Efficiency
**Stramenopiles (Diatoms, Bacillariophytes, Raphidophytes)**			
Thalassiosira pseudonana	*E. coli* EPI300	n/a	~10%
Heterosigma akashiwo	Agrobacterium	10-15%	n/a
**Alveolates**			
Oxyrrhis marina	*E. coli*	100%	1-5%
Karlodinium veneficum	*E. coli*	100%	n/a

Alexandrium catenella	*E. coli*	100%	n/a

F. Glass bead abrasion

Species	Co-incubation (species co-incubated)	Survival rate	Efficiency
**Stramenopiles (Diatoms, Bacillariophytes, Raphidophytes)**			
Heterosigma akashiwo	n/a	80%	n/a
**Alveolates**			
Perkinsus marinus	n/a	80-90%	0.01%-1%
Hematodinium sp.	n/a	40-50%	0%
Amphidinium carterae	None	No data	0%
	Polyethylene glycol	No data	0%

Suppl. Table 5: List of protists selected for this study including links to their transformation protocols (protocols.io) and vector sequences. For contacting particular laboratories, see Suppl. Table 6. For the vector sequences and maps, see Suppl. Notes 1.

Species	Source of organism/ Strain/Culture collection number	Principal Investigator (PI)	Other Investigators	protocols.io links (including construct maps and their sequences)	Sequences submitted (Accession No.) / published
**Archaeplastids**					
Ostreococcus lucimarinus	RCC802	François-Yves Bouget	Jean-Claude Lozano<br>Valérie Vergé	https://www.protocols.io/view/selection-of-stable-transformants-in-ostreococcus-zj2f4qe<br>https://www.protocols.io/view/transient-luciferase-expression-in-ostreococcus-ot-hcib2ue<br>https://www.protocols.io/view/transient-transformation-of-ostreococcus-species-o-g86bzze<br>http://dx.doi.org/10.17504/protocols.io.g86bzze	*p*HAPT:luc vector (Djouani-Tahri *et al*., 2011) was used as a template for preparation of linear construct
Bathycoccus prasinos	RCC4222	François-Yves Bouget	Jean-Claude Lozano<br>Valérie Vergé	https://www.protocols.io/view/selection-of-stable-transformants-in-ostreococcus-zj2f4qe<br>http://dx.doi.org/10.17504/protocols.io.hcib2ue<br>http://dx.doi.org/10.17504/protocols.io.g86bzze	*p*HAPT:luc vector (Djouani-Tahri *et al*., 2011)was used as a template for preparation of linear construct
Micromonas commoda[1]	CCMP 2709 (genome sequenced, axenic version of RCC299)	Alexandra Z. Worden	Manuel Ares<br>Jian Guo<br>Lisa Sudek	http://dx.doi.org/10.17504/protocols.io.8p9hvr6<br>http://dx.doi.org/10.17504/protocols.io.8p8hvrw	
Micromonas pusilla	CCMP 1545	François-Yves Bouget<br>Alexandra Z. Worden	Manuel Ares<br>Jian Guo<br>Jean-Claude Lozano<br>Lisa Sudek<br>Valérie Vergé	https://www.protocols.io/view/plasmid-dnas-designed-for-expression-in-micromonas-i9wch7e	
Tetraselmis striata	KAS-836	Heriberto Cerutti<br>Thomas Clemente	Patrick Beardslee<br>Fulei Luan<br>Xiaoxue Wen	http://dx.doi.org/10.17504/protocols.io.hjtb4nn	GenBank (KY886895)
Pyramimonas parkeae	SCCAP K-0007	Vladimír Hampl	Natalia Ewa Janowicz<br>Anna M.G. Novák Vanclová	https://www.protocols.io/view/protocols-for-mrna-electroporation-hh4b38w<br>https://www.protocols.io/view/nucleofection-of-pyramimonas-parkeae-chromera-veli-ibucanw<br>https://www.protocols.io/view/biolistic-transformation-experiment-on-eutreptiell-ibvcan6	

**Haptophytes**					
Isochrysis galbana	CCMP 1323	Colin Brownlee	Cecilia Balestreri<br>Andrea Highfield<br>Rowena Stern<br>Glen Wheeler	https://www.protocols.io/view/biolistic-transformation-of-isochrysis-galbana-2pugdnw<br>https://www.protocols.io/view/method-for-electroporation-of-isochrysis-galbana-c-hmab42e	GenBank<br>(MK903009 - pIgNAT construct)<br>(MK903010 -PCR product of transgene)
Emiliania huxleyi	CCMP 1516	Colin Brownlee	Cecilia Balestreri<br>Andrea Highfield<br>Rowena Stern<br>Glen Wheeler	http://dx.doi.org/10.17504/protocols.io.8tzhwp6	
**Rhizarians**					
Amorphochlora (Lotharella) amoebiformis	CCMP 2058	Yoshihisa Hirakawa	Kodai Fukuda	http://dx.doi.org/10.17504/protocols.io.35hgq36	
Bigelowiella natans	CCMP 2755	Vladimír Hampl	Natalia Ewa Janowicz<br>Anna M.G. Novák Vanclová	https://www.protocols.io/view/protocols-for-mrna-electroporation-hh4b38w<br>https://www.protocols.io/view/nucleofection-of-pyramimonas-parkeae-chromera-veli-ibucanw<br>https://www.protocols.io/view/biolistic-transformation-experiment-on-eutreptiell-ibvcan6	
**Stramenopiles**					
Fragilariopsis cylindrus	CCMP 1102	Thomas Mock	Amanda Hopes	http://dx.doi.org/10.17504/protocols.io.z39f8r6<br>https://www.protocols.io/view/biolistic-transformation-of-polar-diatom-fragilari-z39f8r6	
Thalassiosira pseudonana	CCMP 1335	Christopher L. Dupont<br>Pamela Silver	Jernej Turnsek	http://dx.doi.org/10.17504/protocols.io.jfncjme<br>http://dx.doi.org/10.17504/protocols.io.nbzdap6<br>http://dx.doi.org/10.17504/protocols.io.7ghhjt6	
Seminavis robusta	DCG 0498 (D6)<br>DCG 0514 (VM3-4)	Aaron Turkewitz	Luke Noble<br>Matthew Rockman<br>Lev Tsypin	http://dx.doi.org/10.17504/protocols.io.4p8gvrw	
Pseudo-nitzschia multiseries	MLML-EBL culture collection, strain 15091C3<br>Unavailable due to culture collapse (after 3 years of cultivation), but DNA and RNA stocks are available	G. Jason Smith	Deborah Robertson<br>April Woods	http://dx.doi.org/10.17504/protocols.io.7vhhn36	http://dx.doi.org/10.17504/protocols.io.7vhhn36

Heterosigma akashiwo	CCMP 2393	Kathryn Coyne	Pamela Green	http://dx.doi.org/10.17504/protocols.io.4qggvtw<br>http://dx.doi.org/10.17504/protocols.io.4qhgvt6<br>https://www.protocols.io/view/modified-genomic-dna-extraction-method-for-heteros-himb4c6<br>https://www.protocols.io/view/modified-total-rna-extraction-for-heterosigma-akas-hipb4dn<br>https://www.protocols.io/view/efforts-to-transform-heterosigma-akashiwo-using-an-4ytgxwn	
Aurantiochytrium limacinum	ATCC MYA-1381	Jackie Collier	Joshua Rest<br>Mariana Rius	http://dx.doi.org/10.17504/protocols.io.8xyhxpw<br>http://dx.doi.org/10.17504/protocols.io.hg6b3ze<br>http://dx.doi.org/10.17504/protocols.io.pgtdjwn	https://www.addgene.org/Jackie_Collier/<br>(for pUC19_GZG, pUC19_18GZG)
Caecitellus sp.	Unavailable due to culture collapse	Patrick Keeling	Elisabeth Hehenberger<br>Nicholas A. T. Irwin	https://www.protocols.io/view/electroporation-of-caecitellus-sp-with-fitc-dextra-35kgq4w	
Nannochloropsis oceanica	CCMP 1779	Peter von Dassow	Fernan Federichi<br>Isaac Nuñez<br>Tamara Matute<br>Albane Ruaud<br>Jorge Ibañez	http://dx.doi.org/10.17504/protocols.io.7r8hm9w<br>http://dx.doi.org/10.17504/protocols.io.h3nb8me	https://doi.org/10.5281/zenodo.3463694
Phaeodactylum tricornutum	CCAP1055/1	Andrew E. Allen	Mark Moosburner<br>Chris Bowler	http://dx.doi.org/10.17504/protocols.io.4abgsan<br>http://dx.doi.org/10.17504/protocols.io.4acgsaw<br>http://dx.doi.org/10.17504/protocols.io.4bmgsk6<br>http://dx.doi.org/10.17504/protocols.io.7gihjue	http://dx.doi.org/10.17504/protocols.io.7gnhjve
**Alveolates**					
Euplotes crassus	CCAP 1624/31	Cristina Miceli	Rachele Cesaroni<br>Lawrence A. Klobutcher<br>Mariusz Nowacki<br>Angela Piersanti<br>Sandra Pucciarelli<br>Estienne Swart	https://www.protocols.io/view/euplotes-miceli-lab-2a8gahw/protocols	
Euplotes focardii	CCAP 1624/34	Cristina Miceli	Angela Piersanti<br>Sandra Pucciarelli	https://www.protocols.io/view/euplotes-miceli-lab-2a8gahw/protocols	
Chromera velia	CCMP 2878	Vladimír Hampl	Natalia Ewa Janowicz<br>Anna M.G. Novák Vanclová	https://www.protocols.io/view/protocols-for-mrna-electroporation-hh4b38w	

				https://www.protocols.io/view/nucleofection-of-pyramimonas-parkeae-chromera-veli-ibucanw<br>https://www.protocols.io/view/biolistic-transformation-experiment-on-eutreptiell-ibvcan6	
Perkinsus marinus[2]	ATCC PRA240	José A. Fernández Robledo<br>Senjie Lin<br>Ross Waller	Duncan B. Coles<br>Elin Einarsson<br>Nastasia J. Freyria<br>Sebastian Gornik<br>Imen Lassadi<br>Arnab Pain	https://www.protocols.io/view/oyster-parasite-perkinsus-marinus-transformation-u-gv9bw96<br>https://www.protocols.io/view/glass-beads-based-transformation-protocol-for-perk-g36byre<br>https://www.protocols.io/view/fluorescence-activated-cell-sorting-facs-of-perkin-hh2b38e<br>https://www.protocols.io/view/golden-gate-plasmids-used-for-transfection-of-perk-37egrje	Genebank<br>(EF632302, EF632303, KX423758–60)
Oxyrrhis marina	CCMP 1788<br>CCMP 1795	Patrick Keeling<br>Claudio Slamovits<br>Senjie Lin	Elizabeth C. Cooney<br>Nicholas A. T. Irwin<br>Elisabeth Hehenberger<br>Yoshihisa Hirakawa<br>Brittany Sprecher<br>Lu Wang<br>Huan Zhang	https://www.protocols.io/view/transfection-of-alexa488-labelled-dna-into-oxyrrhi-ha8b2hw<br>https://www.protocols.io/view/calcium-phosphate-transfection-of-oxyrrhis-marina-ha4b2gw<br>https://www.protocols.io/view/electroporation-transformation-of-fitc-dextran-int-3cmgiu6<br>https://www.protocols.io/view/Dinoflagellate-transformation-e6bbhan<br>https://www.protocols.io/view/co-incubation-protocol-for-transforming-heterotrop-7pphmmn<br>https://www.protocols.io/view/electroporation-of-oxyrrhis-marina-vcne2ve<br>https://www.protocols.io/view/co-incubation-protocol-for-transforming-heterotrop-hmzb476	
Hematodinium sp.	Submitted to ATCC collection (in the meantime please contact Waller´s lab if interested)	Ross Waller	Sebastian Gornik<br>I lan Hu<br>Imen Lassadi<br>Arnab Pain	https://www.protocols.io/view/plasmid-used-for-transfection-trails-of-hematodini-4nigvce	

Fugacium (Symbiodinium) kawagutii	CCMP 2468	Senjie Lin	Brittany Sprecher<br>Lu Wang<br>Huan Zhang	https://www.protocols.io/view/dinoflagellate-transformation-7prhmm6	
Alexandrium catenella	CCMP BF-5	Senjie Lin	Brittany Sprecher<br>Lu Wang<br>Huan Zhang	https://www.protocols.io/view/dinoflagellate-transformation-7prhmm6<br>https://www.protocols.io/view/nucleofector-protocol-for-dinoflagellates-using-lo-7n8hmhw	
Karlodinium veneficum	CCMP 1975	Senjie Lin	Brittany Sprecher<br>Huan Zhang	https://www.protocols.io/view/dinoflagellate-transformation-7prhmm6<br>https://www.protocols.io/view/nucleofector-protocol-for-dinoflagellates-using-lo-7n8hmhw	
Breviolum (*Symbiodinium*) sp.	NIES-4271	Jun Minagawa	Yuu Ishii<br>Konomi Kamada<br>Shinichiro Maruyama	https://www.protocols.io/view/electroporation-of-fluorescein-into-the-coral-symb-hdcb22w	https://www.protocols.io/view/dna-construct-for-genetic-transformation-of-the-co-7udhns6/document
Crypthecodinium cohnii	CCMP 316	José Fernández Robledo<br>Ross Waller	Duncan B. Coles<br>Nastasia J. Freyria<br>Paulo A. García<br>Imen Lassadi	https://www.protocols.io/view/transfection-of-crypthecodinium-cohnii-using-label-z26f8he	Probe amplified from EF632302
Amphidinium carterae	CCMP 1314	Christopher Howe	Adrain Barbrook<br>Isabel Nimmo<br>Ellen Nisbet	http://dx.doi.org/10.17504/protocols.io.4r2gv8e<br>https://www.protocols.io/view/biolistic-transformation-of-amphidnium-hnmb5c6	
**Discobans (Euglenozoans and Heteroloboseans)**					
Bodo saltans	submitted to ATCC collection	Virginia Edgcomb	Miguel A. Chiurillo<br>Roberto Decampo<br>Fatma Gomaa<br>Noelia Lander<br>Zhuhong Li	http://dx.doi.org/10.17504/protocols.io.s5peg5n<br>http://dx.doi.org/10.17504/protocols.io.s5meg46<br>http://dx.doi.org/10.17504/protocols.io.s5jeg4n<br>http://dx.doi.org/10.17504/protocols.io.sh4eb8w<br>http://dx.doi.org/10.17504/protocols.io.sh6eb9e	http://dx.doi.org/10.17504/protocols.io.7fchjiw<br><br>GenBank (MN608152)
Diplonema papillatum	ATCC 50162	Julius Lukeš	Drahomíra Faktorová<br>Ambar Kachale<br>Binnypreet Kaur<br>Getraud Burger<br>Matus Valach	https://www.protocols.io/groups/julius-lukes<br>https://dx.doi.org/10.17504/protocols.io.4digs4e	GenBank (MN047315)
Eutreptiella gymnastica	SCCAP K-0333	Vladimír Hampl	Natalia Ewa Janowicz<br>Anna M.G. Novák Vanclová	https://www.protocols.io/view/protocols-for-mrna-electroporation-hh4b38w	

				https://www.protocols.io/view/nucleofection-of-pyramimonas-parkeae-chromera-veli-ibucanw<br>https://www.protocols.io/view/biolistic-transformation-experiment-on-eutreptiell-ibvcan6	
Naegleria gruberi	ATCC 30224	Anastasios Tsaousis	Veronica Freire-Beneitez<br>Eleanna Kazana<br>Jan Pyrih<br>Tobias von der Haar	http://dx.doi.org/10.17504/protocols.io.hnhb5b6<br>http://dx.doi.org/10.17504/protocols.io.hpub5nw<br>http://dx.doi.org/10.17504/protocols.io.hpvb5n6	http://dx.doi.org/10.17504/protocols.io.7w4hpgw
**Opisthokonts**					
Pirum gemmata	ATCC PR-280	Elena Casacuberta<br>Iñaki Ruiz-Trillo	Cristina Aresté	http://dx.doi.org/10.17504/protocols.io.z5nf85e	
Sphaeroforma arctica	ATCC PRA-297	Elena Casacuberta<br>Iñaki Ruiz-Trillo	Cristina Aresté<br>Omaya Dudin	http://dx.doi.org/10.17504/protocols.io.z5nf85e<br>http://dx.doi.org/10.17504/protocols.io.z6ef9be	
Abeoforma whisleri	ATCC PRA-279	Elena Casacuberta<br>Iñaki Ruiz-Trillo	Elena Casacuberta<br>Cristina Aresté<br>Sebastián Najle	https://www.protocols.io/view/abeoforma-whisleri-transient-transfection-protocol-zexf3fn<br>http://dx.doi.org/10.17504/protocols.io.zexf3fn	
Salpingoeca rosetta	ATCC PRA-390	Nicole King	David Booth<br>Monika Sigg	http://dx.doi.org/10.17504/protocols.io.h68b9hw	

-American Type Culture Collection (ATCC) (https://www.atcc.org/)
-Culture Collection of Marine Phytoplankton (CCMP) now The Provasoli-Guillard National Center for Marine Algae and Microbiota (NCMA) (https://ncma.bigelow.org/cms/index/index/)
-Kuehnle AgroSystems Inc (KAS) (http://www.kuehnleagro.com/)
-Scandinavian Culture Collection of Algae and Protozoa (SCCAP) (http://www.sccap.dk/dk/soeg/detaljer.asp?Cunr=K-0007)
-Microbial Culture Collection at the National Institute for Environmental Studies (NIES Collection, Tsukuba, JAPAN) (https://mcc.nies.go.jp/)
-Culture collection of the BCCM-DCC (http://bccm.belspo.be/about-us/bccm-dcg)
-Culture collection MLML-EBL (https://www.mlml.calstate.edu/ebl/)

Reference

Djouani-Tahri, el B., Sanchez, F., Lozano, J. C., Bouget, F. Y. (2011). A phosphate-regulated promoter for fine-tuned and reversible overexpression in *Ostreococcus*: application to circadian clock functional analysis. *PLoS One* **6**: e28471.

Details of protocol for particular species:

[1]*Micromonas commoda*: Acclimated mid-exponential M. commoda cells grown in L1 medium at 21 oC were spun at 5000 x g for 10 min, the pellet was resuspended in Buffer SF (Lonza) premixed with carrier DNA (pUC19) and plasmid, and 3 x 107 cells were used per reaction. After applying the EW-113 pulse, 100 µl of ice-cold recovery buffer (10 mM HEPES-KOH, pH 7.5; 530 mM sorbitol; 4.7% [w/v] PEG 8000) was added to each well and incubated for 5 min at room temperature. Each reaction was then transferred into 2 ml L1, placed at 21 °C and light was increased stepwise over 72 h.

[2]*Perkinsus marinus*: In brief, a newly formulated transformation 3R buffer (200 mM Na2HPO4; 70 mM NaH2PO4; 15 mM KCl; 1.5 mM CaCl2; 150 mM HEPES-KOH, pH 7.3) was used to reduce the cost of electroporation. 5 x 107 cells were resuspended in 330 µl of fresh ATCC Medium 1886 and were mixed with 5.0 µg of linearized and circular [1:1] plasmid and 300 µl of glass beads (Sigma) in a 1.5 ml tube, vortexed for 30 s at maximum speed, and cells in 500 µl of culture medium were transferred to 6-well plates in a final volume of 3 ml.

[3]*Bodo saltans*: Square-wave electroporation (Nepa21) was used with a poring pulse of 250V (25 ms) and 5 transfer pulses of 60V (99 ms) in the presence of Cytomix buffer (120 mM KCl; 0.15 mM CaCl2; 10 mM KH2PO4; 2 mM EGTA; 5 mM MgCl2; 25 mM HEPES-KOH, pH 7.6).
