# Supplementary material for: Genetic tool development in marine protists: emerging model organisms for experimental cell biology
Source: Nat Methods. 2020 Apr 6;17(5):481–94. doi: 10.1038/s41592-020-0796-x (PMC7200600; doi:10.1038/s41592-020-0796-x)
Supplement: Supplementary file 6 — Source Data Fig. 4a–c,e–j [file 41592_2020_796_MOESM6_ESM.pdf]

Source data - Fig. 4a - ***Isochrysis galbana***

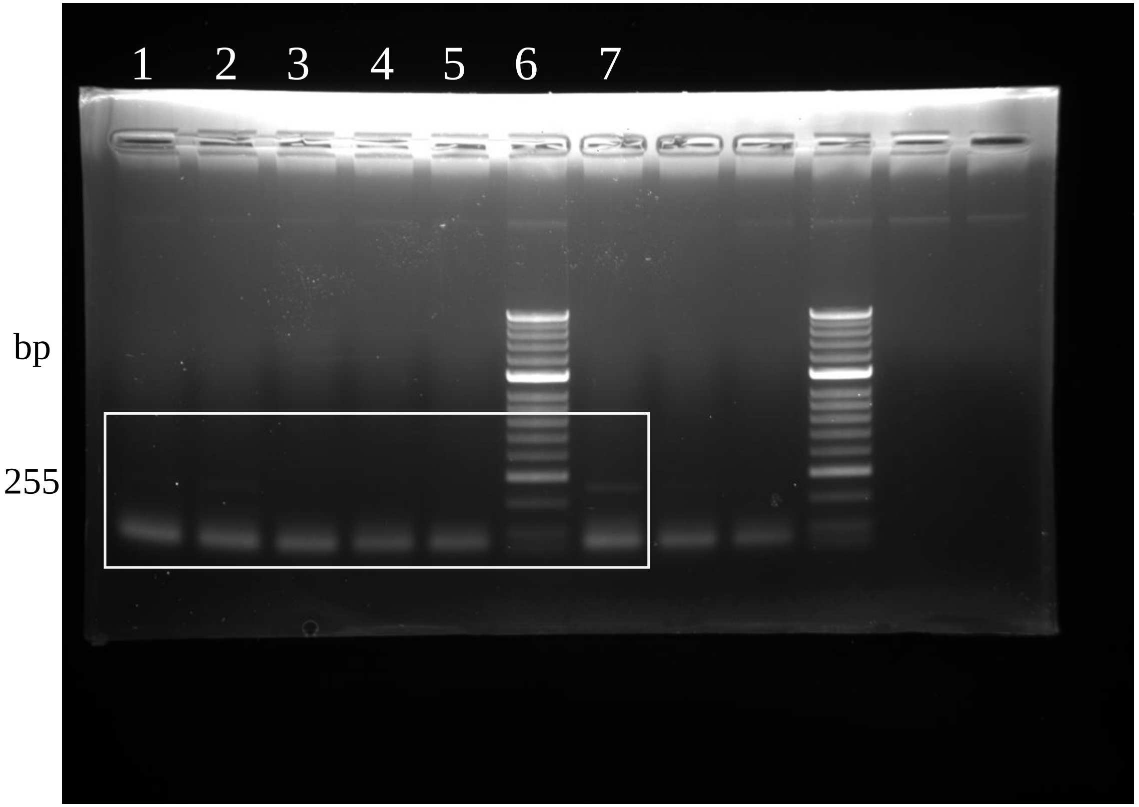

Rectangle shows the part of the gel shown in Fig. 4a. The additional lanes belong to a separate experiment.

Fig. 4 | Various methods were used to demonstrate successful transformation in different species.

(**a**) Expression of nourseothricin N-acetyl transferase (*NAT*) in pIgNAT transformed *I. galbana*, from cells exhibiting resistance to nourseothricin. Expression of the *NAT* transgene in WT (lane 3) and transformants (lanes 1 and 2) of *I. galbana* verified by RT-PCR. RT- negative and no template controls are shown in lanes 4 and 5, respectively, and 50 bp ladder (Bioline, lane 6), positive control PCR of genomic DNA from transformed cells (lane 7). The RT-PCR was performed once on two independently transformed *I. galbana* lines.

Source data - Fig. 4b – ***Amorphochlora amoebiformis***

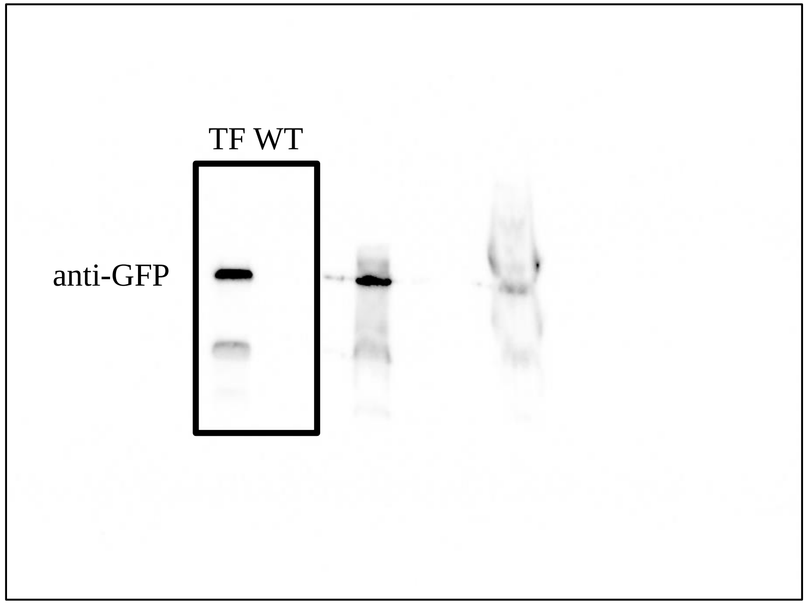

Black rectangle shows the part of the membrane shown in Fig. 4b.

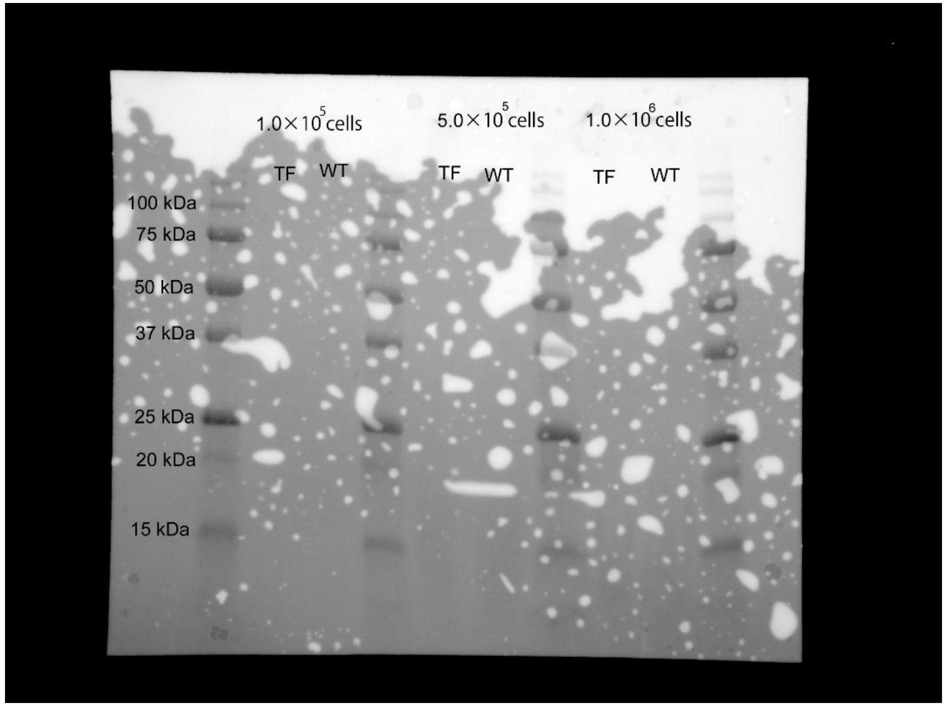

Fig. 4 | Various methods were used to demonstrate successful transformation in different species.

(**b**) Western blot of expressed eGFP in *A. amoebiformis*, with total proteins extracted from transformants (TF) and WT cells using α-GFP monoclonal antibody (Takara; 1:1,000), and an α-mouse horseradish peroxidase (HRP)-coupled antibody (GE Healthcare; 1:10,000). Precision plus protein standard (Biorad, 161-0374) was used. A representative image from two independent experiments is shown.

Source data - Fig. 4c – ***Fragilariopsis cylindrus***

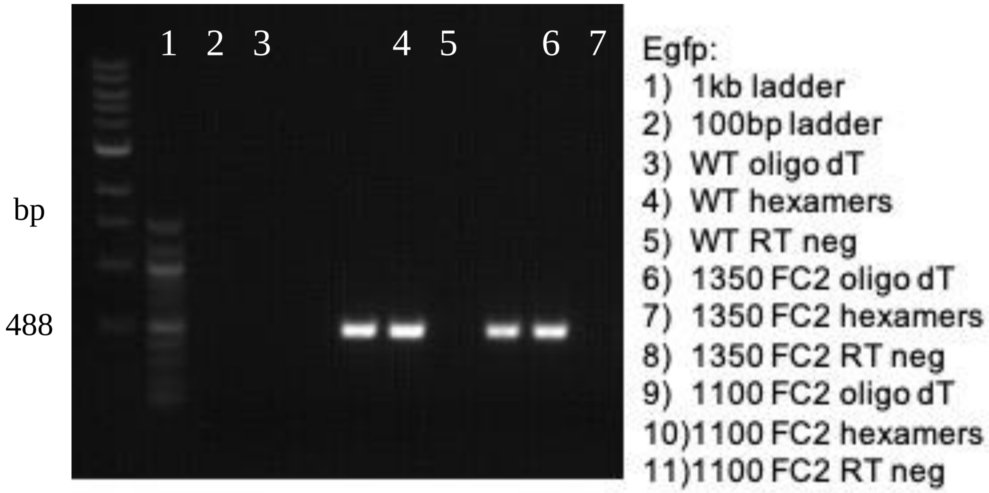

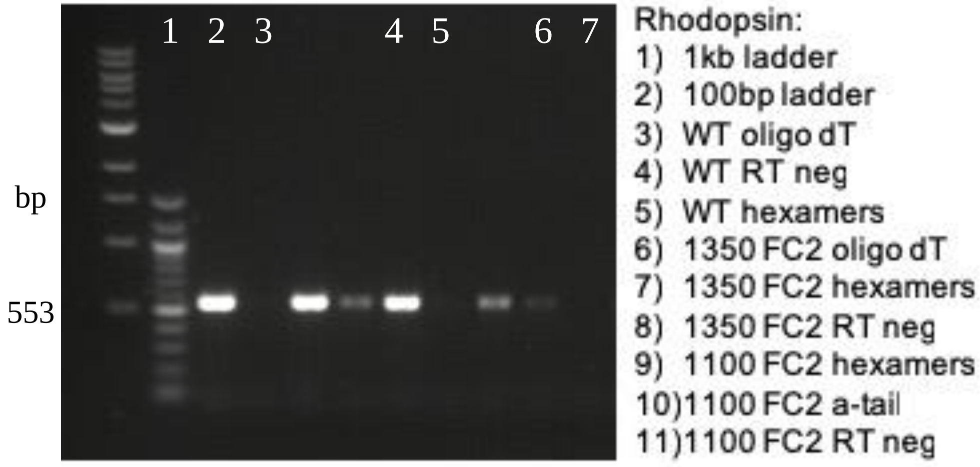

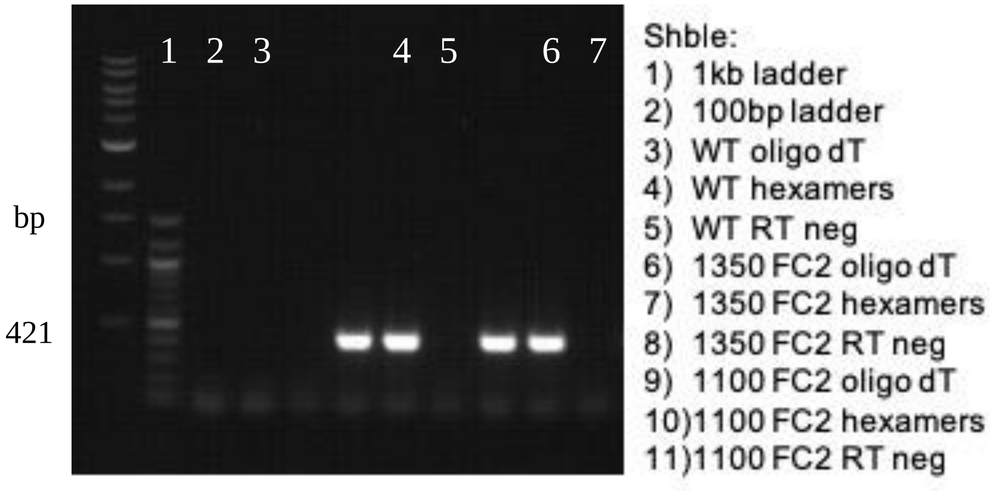

Lanes labelled with numbers are shown in Fig. 4c.

Fig. 4 | Various methods were used to demonstrate successful transformation in different species.

(**c**) Expression of *eGFP*, *ShBle* and *Rhodopsin* (endogenous gene used as control) in WT (lane 2) and transformants (1350 FC2: lane 4, 1100 FC2: lane 6) of *F. cylindrus* verified by RT-PCR. RT- negative controls are shown in lanes 3, 5 and 7. The 100 bp ladder (NEB) is shown in lane 1. RT-PCR was carried out twice on each strain.

Source data - Fig. 4e - ***Karlodinium veneficum***

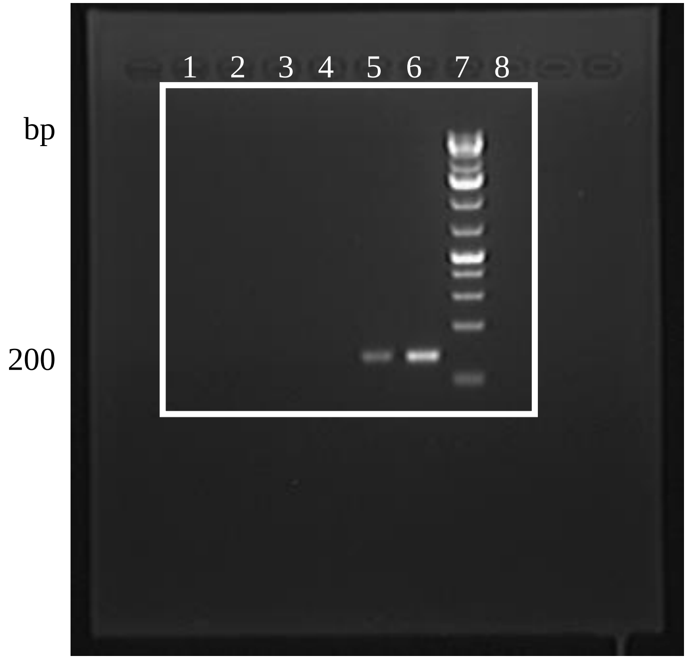

White rectangle shows the part of the gel shown in Fig. 4e.

Fig. 4 | Various methods were used to demonstrate successful transformation in different species.
(**e**) RT-PCR of the *nptII/neo* gene in kanamycin resistant *K. veneficum* transformants. No template negative control (lane 1); RT- negative controls of WT and transformed cells (lanes 2 and 4, respectively); cDNA from WT cells grown without antibiotics (lane 3), cDNA from transformants grown under kanamycin selection (lane 5), PCR positive control using the original vector DNA (DinoIII-*neo*, lane 6), GeneRuler DNA ladder (lane 7). Nothing was loaded in lane 8. cDNA libraries were made with 200 ng of RNA from WT and CA-137 transformant and all PCRs were nested. Three amplification experiments were repeated independently with similar results.

Source data - Fig. 4f – ***Perkinsus marinus***

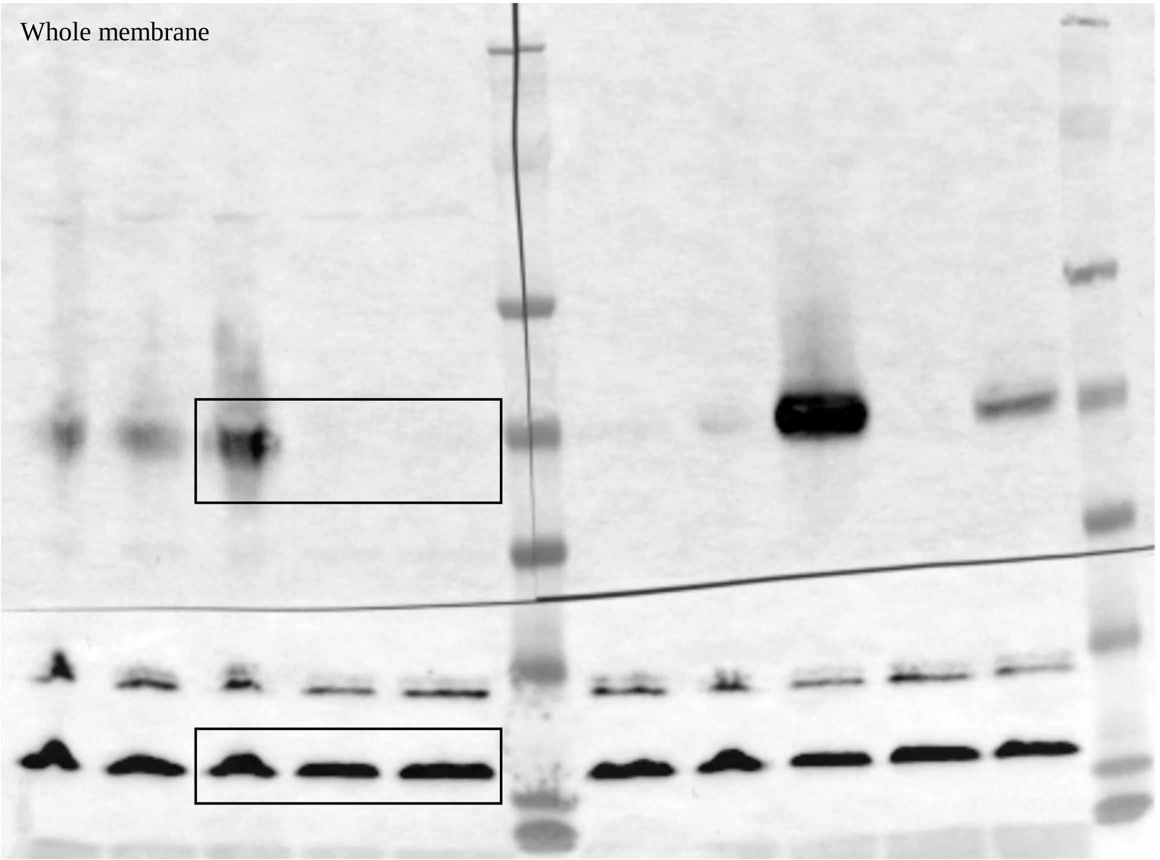

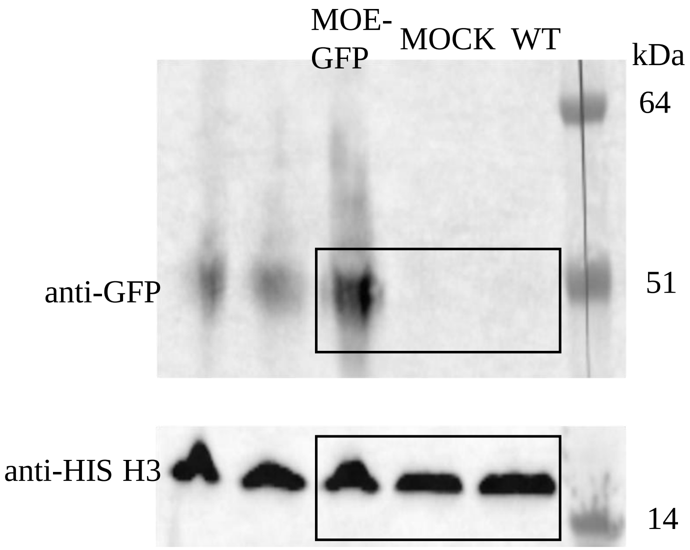

Black rectangles show the part of the membrane depicted in Fig. 4f.

Fig. 4 | Various methods were used to demonstrate successful transformation in different species.

(**f**) Western blot was used to confirm the expression of MOE-GFP (40.3 kDa) in transfected *P. marinus* cells. Mock transfected cells (i.e. electroporated without plasmid) and WT were included as a control. Polyclonal rabbit α-GFP antibody (Invitrogen, 1:1000) and secondary goat α-rabbit coupled to HRP (1:10000) were used for visualization. Histone H3 (polyclonal rabbit, 1:1000, Invitrogen) was used as a loading control. SeeBlue Plus2 Pre-stained protein standard (LC5925, ThermoFisher) was used. The experiment was replicated three times with similar results.

Source data - Fig. 4g - ***Amphidinium carterae***

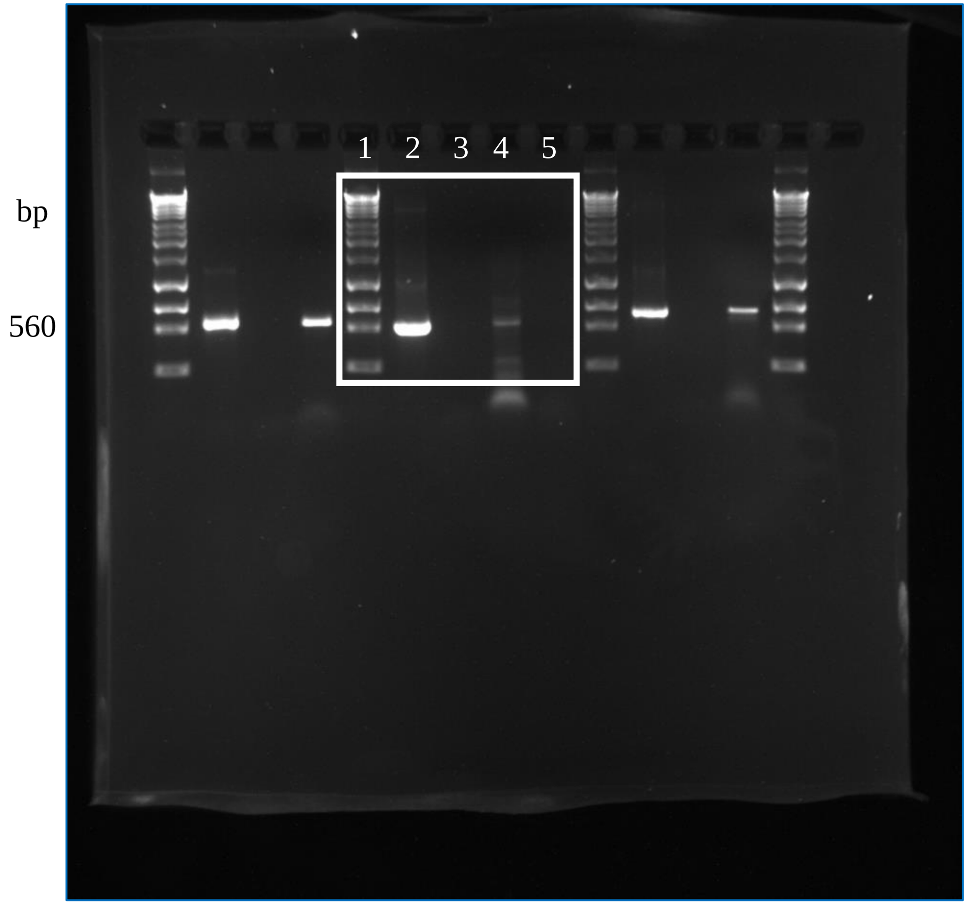

Rectangle shows the part of the gel shown in Fig. 4g.

Fig. 4 | Various methods were used to demonstrate successful transformation in different species.
(**g**) RT-PCR verification of *A. carterae* transformants showing transcription from an artificial minicircle based on atpB. HyperLadder 1 kb plus (Bioline, lane 1), positive control PCR with artificial minicircle template (lane 2), negative control with no template (lane 3), RT-PCR against chloramphenicol acetyltransferase (CAT) transcript from artificial minicircle (RT+, lane 4), RT-PCR against CAT transcript from artificial minicircle (RT- negative control, lane 5). The experiment was replicated twice with similar results.

Source data - Fig. 4h – ***Bodo saltans***

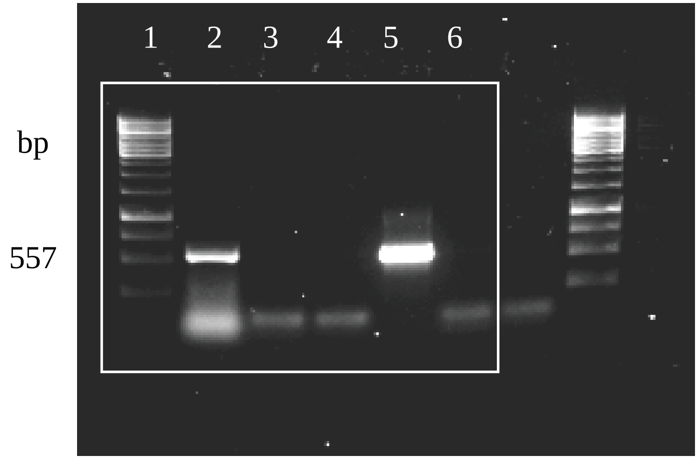

White rectangles show the part of the gel depicted in Fig. 4h.

Fig. 4 | Various methods were used to demonstrate successful transformation in different species.

(**h**) RT-PCR confirming expression of *nptII/neo* gene expression in *B. saltans* transformants. GeneRuler 1 kb DNA Ladder (Thermo Fisher Scientific); lane 1), *nptII/neo* expressed in *B. saltans* cells transformed with EF-1 alpha plasmid (lane 2); RT- negative control from transformed *B. saltans* cells (lane 3), PCR for the EF -1 alpha plasmid on RNA after DNase treatment (to verify absence of DNA, lane 4), PCR positive control using the EF- 1 alpha plasmid DNA (lane 5), negative control with no template (lane 6). NeoF and NeoR primers sequences are listed in Suppl. Fig. 10b. Results were repeated twice independently.

Source data - Fig. 4i – ***Diplonema papillatum***

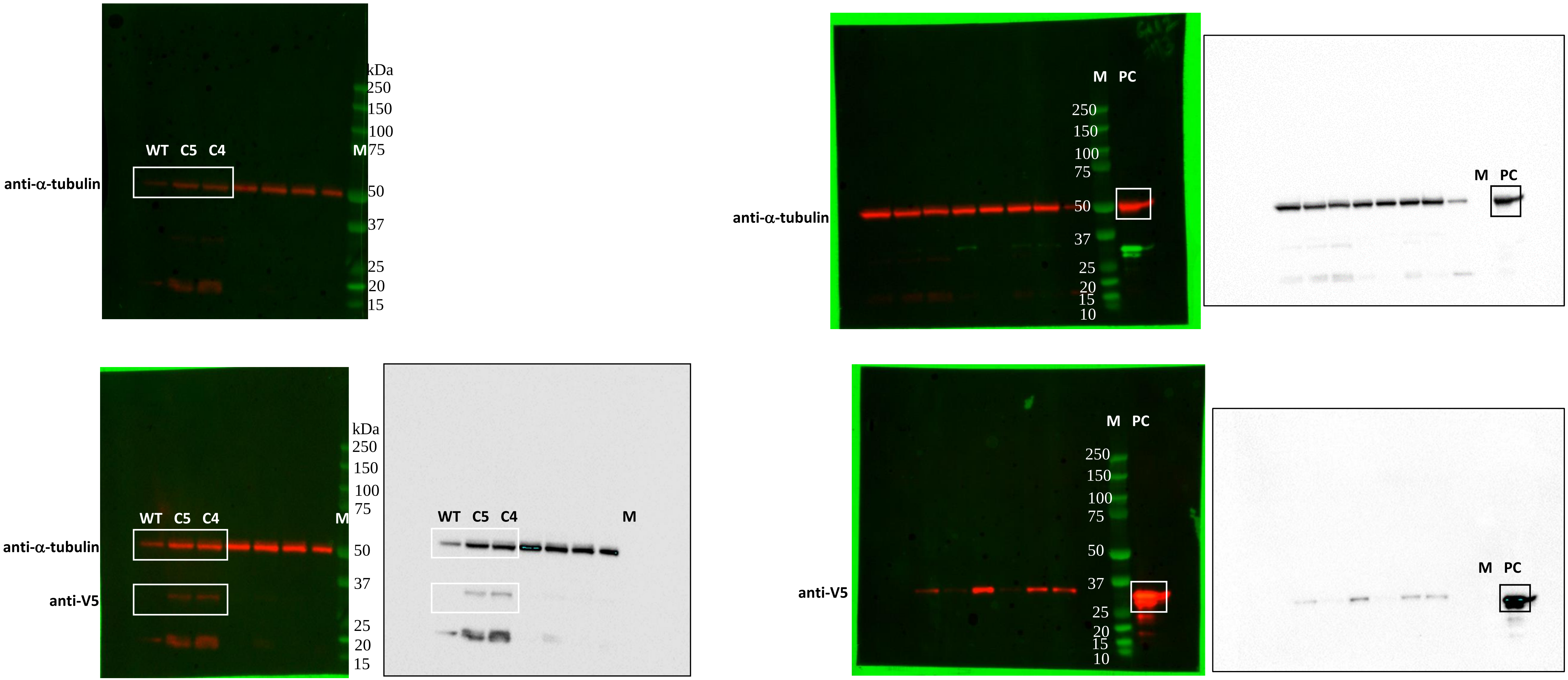

White and black rectangles show the part of the membranes depicted in Fig. 4i. Unlabbeled lines correspond to other samples.

Fig. 4 | Various methods were used to demonstrate successful transformation in different species.

(**i**) Western blot of *D. papillatum* WT and C5 and C4 transformants that express the V5-tagged *nptII/neo* gene. Monoclonal mouse a-V5 antibody (Invitrogen; 1:2,000) and secondary a-mouse HRP-coupled antibody (Sigma; 1:1,000) were used. V5-tagged-mNeonGreen *Trypanosoma brucei* cells served as a positive control and mouse α-alpha-tubulin antibody (Sigma; 1:5,000) was used as a loading control. Precision plus protein standard (Biorad, 161-0374) was used. Results were repeated twice independently.

Source data - Fig. 4j – ***Abeoforma whisleri***

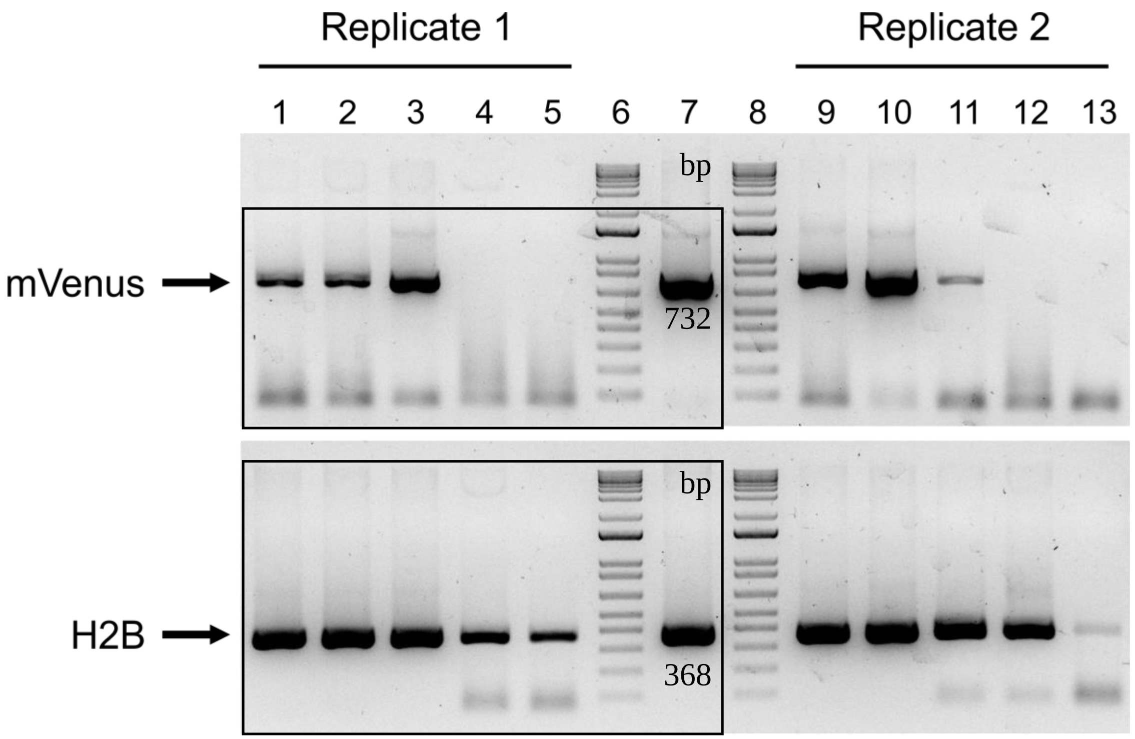

Black rectangles show the part of the gels depicted in Fig. 4j.

Fig. 4 | Various methods were used to demonstrate successful transformation in different species.

(**j**) For top and bottom gels, RT-PCR of *A. whisleri* cells transfected with either 1 mg pAwhi_H2Bvenus vector plus 10 mg carrier DNA (pUC19) (lanes 1-3), carrier DNA only (lane 4) or without DNA (lane 5), and pAwhi_H2Bvenus used as positive control (lane 7). A 1 kb DNA ladder was used (lane 6). Transfection has been repeated more than 30 times and results were repeated twice independently.
