# Supplementary material for: Genetic tool development in marine protists: emerging model organisms for experimental cell biology
Source: Nat Methods. 2020 Apr 6;17(5):481–94. doi: 10.1038/s41592-020-0796-x (PMC7200600; doi:10.1038/s41592-020-0796-x)
Supplement: Supplementary file 7 — Source Data Supplementary Figures [file 41592_2020_796_MOESM7_ESM.pdf]

Source data – Suppl. Fig. 9B – ***Perkinsus marinus***

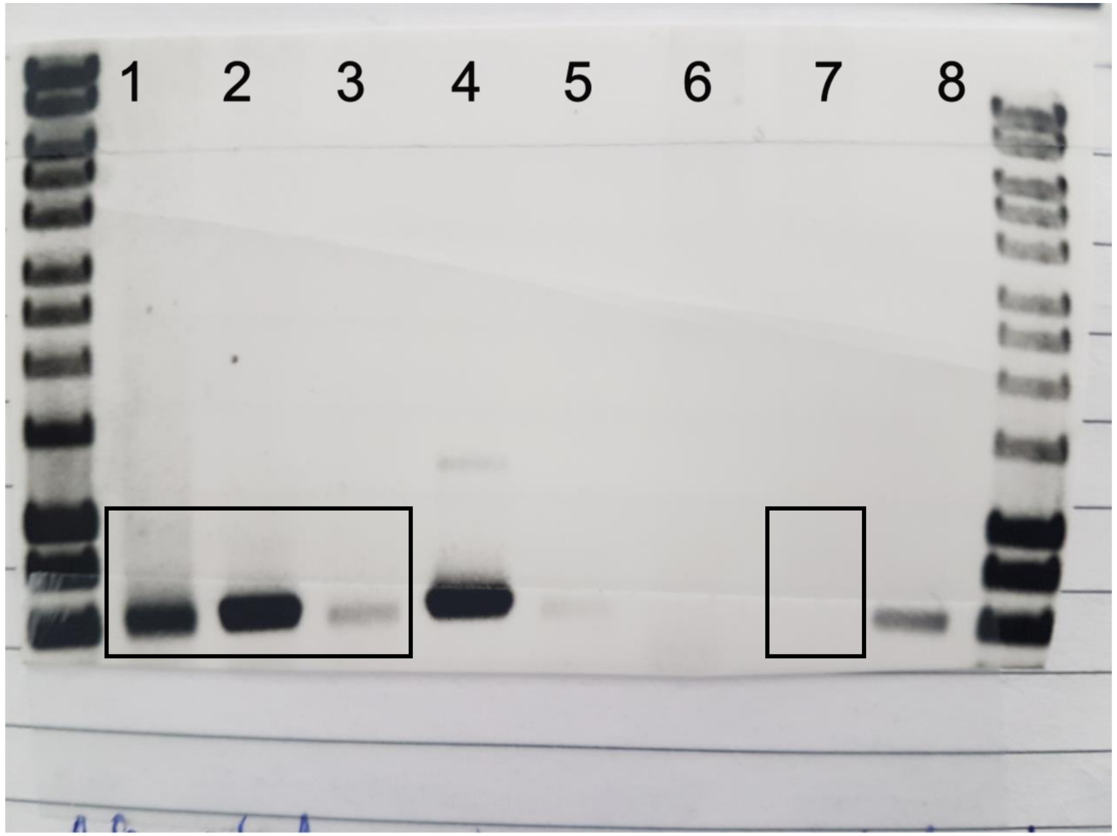

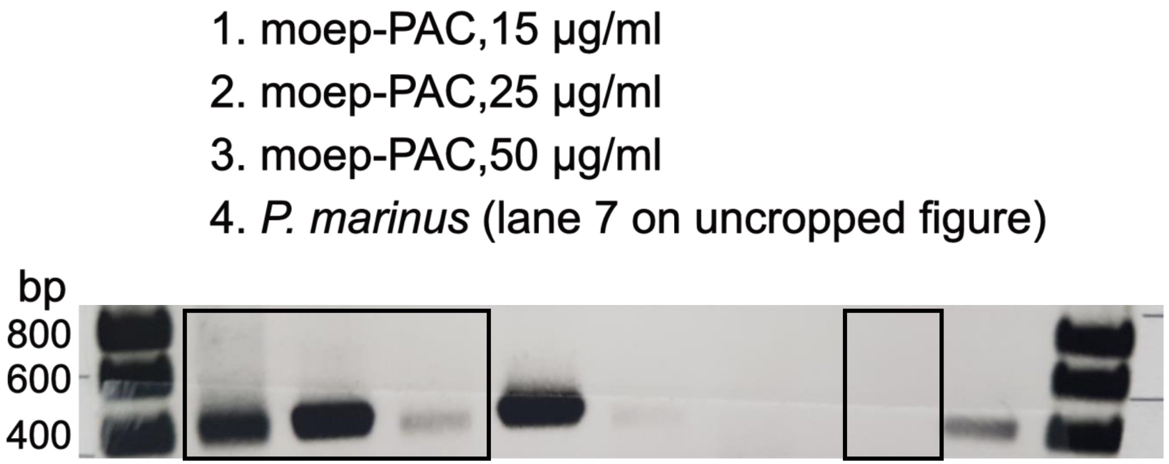

Black rectangles show the part of the gel depicted in Suppl. Fig. 9B.

Suppl. Fig. 9 | Transformation of *Perkinsus marinus*.
B. The puromycin resistance gene (490 bp) can be amplified from gDNA extracted from resistant cell lines that were subjected to different concentrations of puromycin (15, 25 and 50 µg/ml). Non-transformed *P. marinus* was used as a control. HyperLadder 1kb (BIO-33053, Bioline) was used. The experiment was replicated twice with similar results.

Source data – Suppl. Fig. 9C – ***Perkinsus marinus***

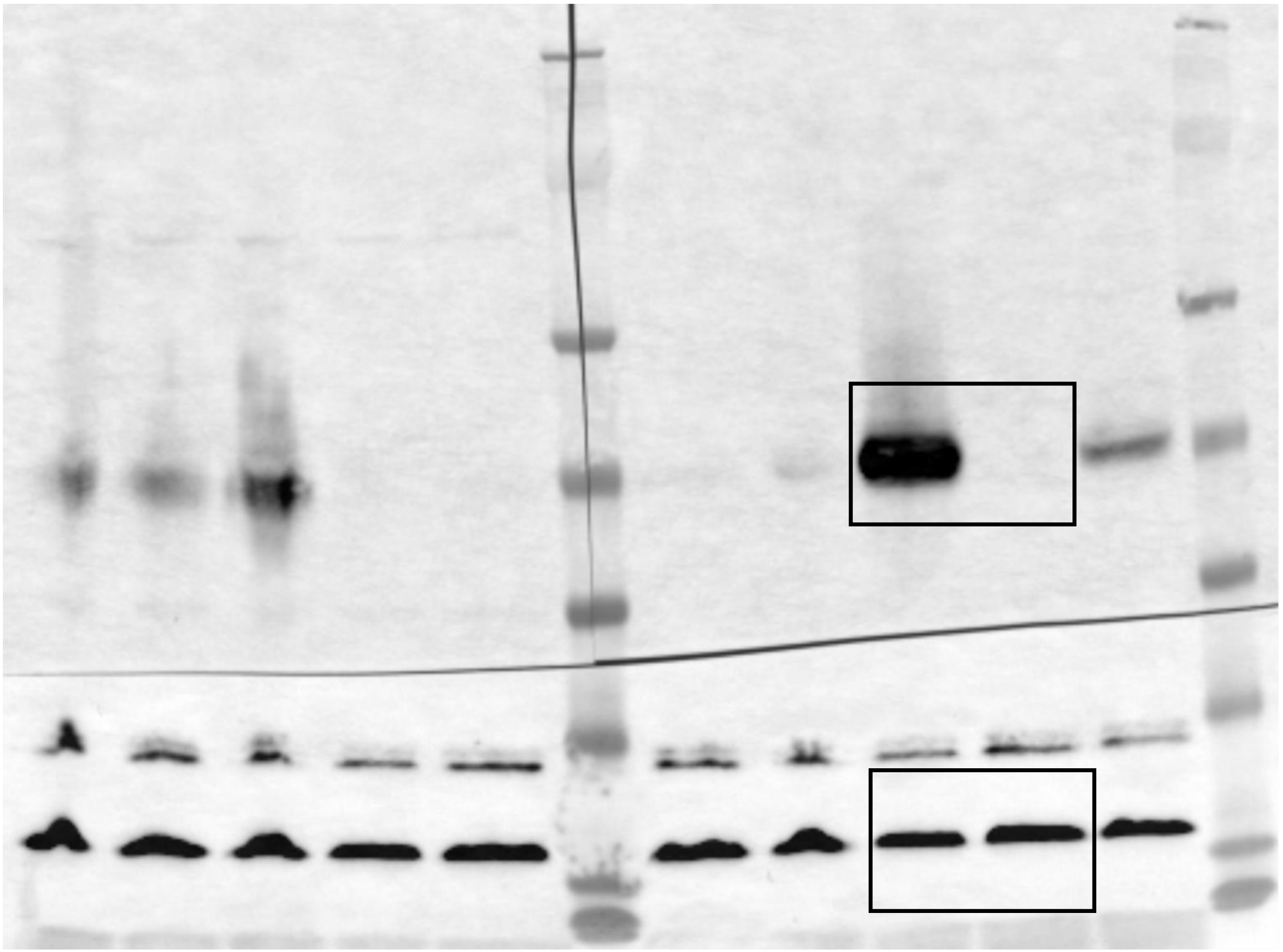

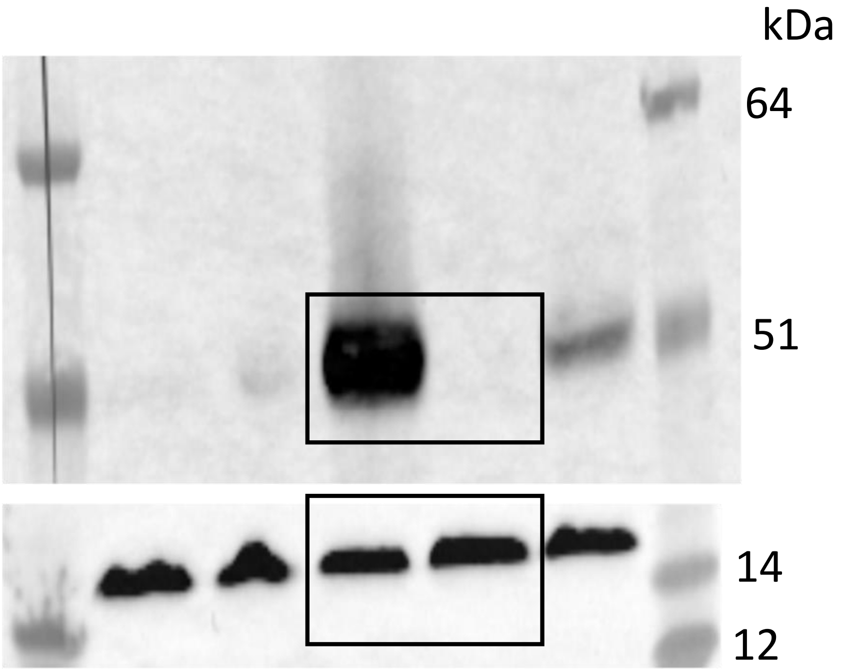

Black rectangles show the part of the membrane depicted in Suppl. Fig. 9C.

Suppl. Fig. 9 | Transformation of *Perkinsus marinus.*
C. Western blot was used to confirm expression of MOE-mCherry (40.4 kDa) in transformed *P. marinus* cells (polyclonal rabbit α-mCherry, 1:1000, Abcam) and Histone H3 (15 kDa) was used as a loading control (polyclonal rabbit α-Histone H3, 1:1000, Invitrogen). SeeBlue Plus2 Pre-stained protein standard (ThermoFisher, LC5925) was used. The experiment was replicated five times with similar results.

Source data – Suppl. Fig. 11A – ***Naegleria gruberi***

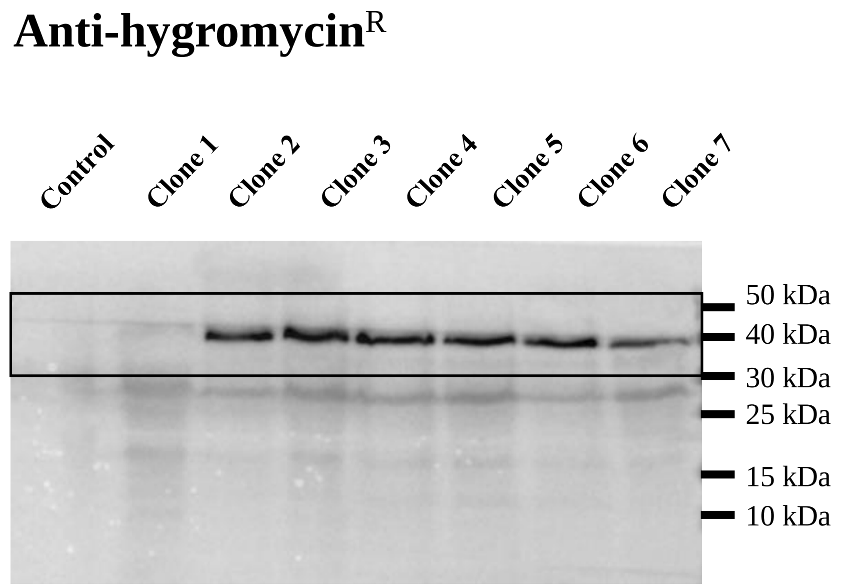

Predicted size of *hph* encoded protein: 38 kDa

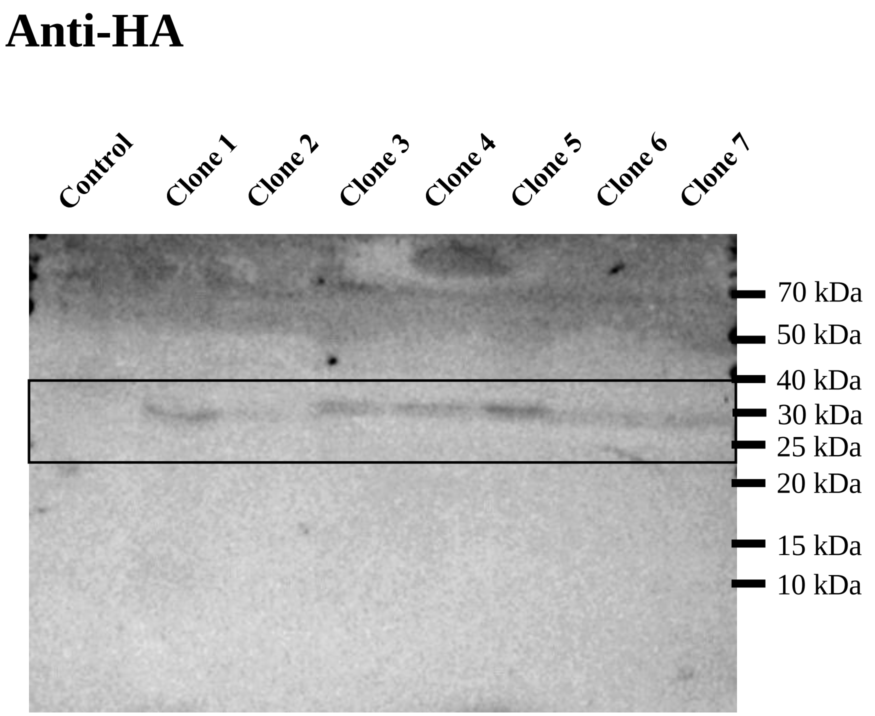

Black rectangles show the part of the membranes depicted in Suppl. Fig. 11A.

Predicted size of eGFP-HA fused protein: 29.5 kDa

Suppl. Fig. 11 | Transformation of *Naegleria gruberi*.
(A) Western blot of transfected clonal cells and the control (non-transfected cells) demonstrating the expression of the HA-tag (anti-HA antibody, Sigma) and hygromycin B resistance gene - *hph* (anti-hygromycin^R antibody, Fitzgerald Industries) in these clones but not in the control. Molecular Marker: PageRuler, Unstained Protein Ladder (Thermo Scientific, Catalog number:26614). Representative results of three independent replicates are shown.

Source data – Suppl. Fig. 12 – ***Diplonema papillatum***

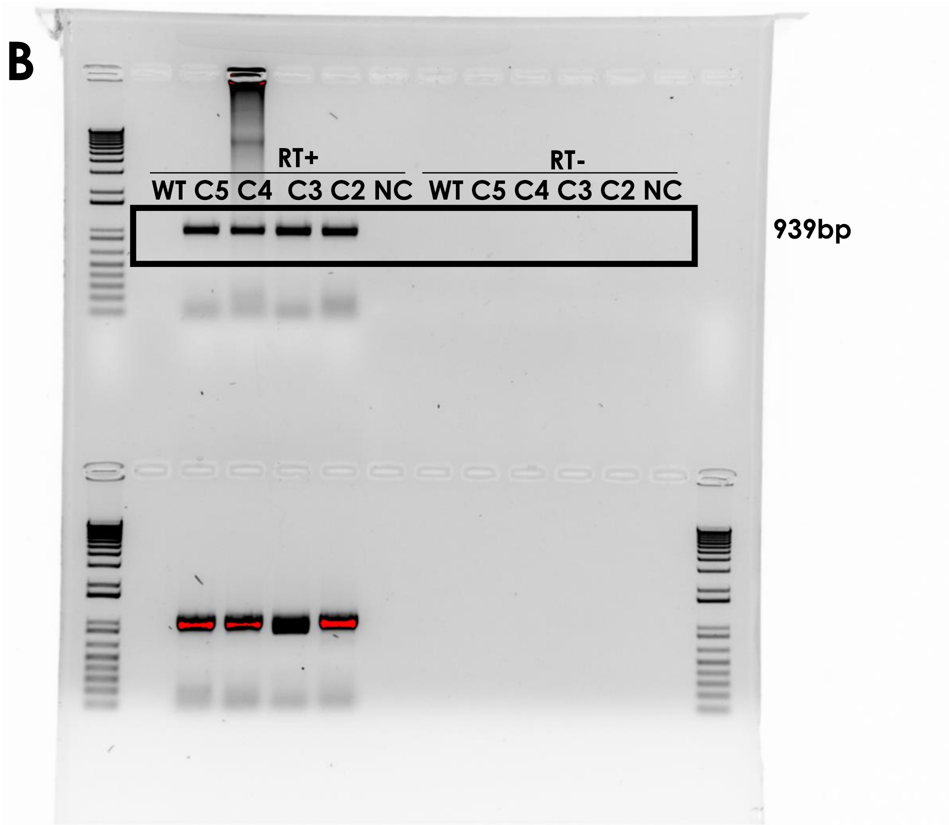

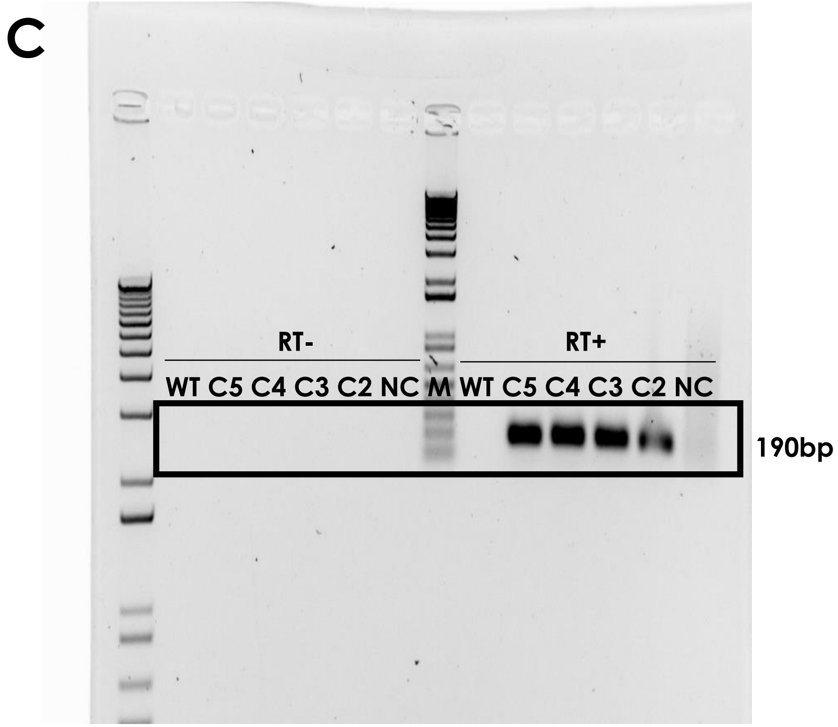

Black rectangles show the part of the gels depicted in Suppl. Fig. 12B and C. Unlabbeled lines correspond to other samples.

Suppl. Fig. 12 | Validation of proper transcription of 3xV5+NeoR heterologous gene in *Diplonema papillatum* by RT-PCR.
(B) RT-PCR amplification of the 3xV5+Neo^R transcript (939 bp-long region) was performed using P1-FW and P1-RV primers. **(C)** RT-PCR amplification of the 3xV5+Neo^R transcript (190 bp-long region) using P2-FW and P2-RV primers. RNA from wild type cells (WT) and transformants A1, A2, A3 and A4 was used as a template for RT-PCR. A sample without cDNA (NC) served as a negative control. Reactions with and without reverse transcriptase are indicated by (RT+) and (RT-). M = 1Kb Plus DNA ladder (Invitrogen, 10381-010) was used. Representative images of two independent experiments are shown.
